# Cytotoxic Acetogenins from the Roots of *Annona purpurea*

**DOI:** 10.3390/ijms20081870

**Published:** 2019-04-16

**Authors:** Gustavo Alejandro Hernández-Fuentes, Aída Nelly García-Argáez, Ana Lilia Peraza Campos, Iván Delgado-Enciso, Roberto Muñiz-Valencia, Francisco Javier Martínez-Martínez, Antonio Toninello, Zeferino Gómez-Sandoval, Juan Pablo Mojica-Sánchez, Lisa Dalla Via, Hortensia Parra-Delgado

**Affiliations:** 1 Facultad de Ciencias Químicas, Universidad de Colima, Carretera Colima-Coquimatlán km 9, 28400 Coquimatlán, Colima, Mexico; gahfuentes@gmail.com (G.A.H.-F.); peraza@ucol.mx (A.L.P.C.); robemuva@yahoo.com (R.M.-V.); fjmartin@ucol.mx (F.J.M.-M.); zgomez@ucol.mx (Z.G.-S.); mojica_sanchez@ucol.mx (J.P.M.-S.); 2 Dipartimento di Scienze del Farmaco, Università degli Studi di Padova, Via F. Marzolo 5, 35131 Padova, Italy; aida_nellyg@hotmail.com; 3 Fondazione per la Biologia e la Medicina della Rigenerazione T.E.S.-Tissue Engineering and Signalling Onlus, Via F. Marzolo, 13, 35131 Padova, Italy; 4 Facultad de Medicina, Universidad de Colima, Av. Universidad 333, Las Víboras, 28040 Colima, Mexico; ivan_delgado_enciso@ucol.mx; 5 Dipartimento di Scienze Biomediche, Università degli Studi di Padova, Via G. Colombo 3, 35121, Padova, Italy; antonio.toninello@unipd.it

**Keywords:** *Annona purpurea*, Annonaceae, acetogenins, annopurpuricins, cytotoxicity, mitochondria

## Abstract

*Annona purpurea*, known in Mexico as “cabeza de negro” or “ilama”, belongs to the Annonaceae family. Its roots are employed in folk medicine in several regions of Mexico. Taking that information into account, a chemical and biological analysis of the components present in the roots of this species was proposed. Our results demonstrated that the dichloromethane (DCM) extract was exclusively constituted by a mixture of five new acetogenins named annopurpuricins A–E (**1**–**5**). These compounds have an aliphatic chain of 37 carbons with a terminal α,β unsaturated γ-lactone. Compounds **1** and **2** belong to the adjacent bis-THF (tetrahydrofuran) α-monohydroxylated type, while compounds **3** and **4** belong to the adjacent bis-THF α,α’-dihydroxylated type; only compound **5** possesses a bis-epoxide system. Complete structure analysis was carried out by spectroscopy and chemical methods. All compounds were evaluated for their antiproliferative activity on three human tumor cell lines (MSTO-211H, HeLa and HepG2). Compounds **1**–**4** inhibited significantly the growth of HeLa and HepG2 cells, showing GI_50_ values in the low/subnanomolar range, while **5** was completely ineffective under the tested conditions. The investigation of the mechanism of action responsible for cytotoxicity revealed for the most interesting compound **1** the ability to block the complex I activity on isolated rat liver mitochondria (RLM).

## 1. Introduction

The family of Annonaceae is widely distributed in tropical areas. In Mexico, 13 genera and 37 species have been reported. Several species of this family have been used in traditional medicine in many countries and they are characterized by the production of alkaloids, terpenes and acetogenins [1,2,3,4,5,6,7,8,9]. The annonaceous acetogenins are naturally occurring metabolites that possess long-chain aliphatic backbone unique structures. The general structure of acetogenins comprises of a terminal α, β-unsaturated γ-lactone and different substituents such as epoxide or tetrahydrofuran (THF) rings along the aliphatic chain [7]. In the acetogenins, these five or six member rings are commonly flanked by hydroxyls, and the presence of double bonds or ketones can be observed [8].

With respect to the biological activities of acetogenins, a wide spectrum of effects has been described, including cytotoxicity on cancer cells and anti-inflammatory activities [10,11,12]. The biological effects of acetogenins are commonly related to their ability to inhibit NADH: ubiquinone oxidoreductase (complex I) of mitochondrial respiratory chain [7], although other mechanisms of action could participate to the overall biological activity [8]. With respect to the interaction between acetogenins and mitochondrial complex I, several studies have been performed by using both natural and synthetic acetogenins, leading to some structure-activity relationships [13,14,15].

*Annona purpurea* Moc & Sessé ex Dunal is a tree reaching 5–10 m, known in Mexico as “cabeza de negro”, “toreta” or “ilama”. Several parts of this species are traditionally used in Mexico in folk medicine. In particular, its bark is used for the treatment of dysentery and edema, and the roots are employed against kidney diseases. Moreover, seeds and leaves have been used to prepare insecticide solutions [5,6].

Previous studies of *A. purpurea* described the isolation of bioactive alkaloids and acetogenins [16,17,18,19,20]. From its leaves, acetogenins like rolliniastatin I, bullatacin, purpureacin, cherimoline and sylvaticin have been identified [16]. Likewise, from seeds purpurediolin, purpurenin, annoglaucin, bullatacin, squamocin (annonin I), motrilin (squamocin C), xylomatenin, annonacin A and purpuracenin have been isolated [19,20]. In addition, aporphine and oxoaporphine alkaloids have been frequently reported in stems and leaves [17,18]. To date, to our knowledge only one study has explored the bioactive components of roots of *A. purpurea* [21]. In this study, only annomontine (0.003% yield), a pyrimidine-β-carboline alkaloid, with anxiolytic effects, was characterized from a total alkaloid extract. Considering the very little chemical and biological information available on *A. purpurea* roots, the purpose of this work was to determine the main components of the roots, as well as their biological effects, against several cancer cells. In detail, the isolation, structure and absolute configuration of five new acetogenins, exclusive components of the DCM (dichloromethane) extract of the roots of *A. purpurea*, are reported. The new acetogenins isolated in the present study were named as annopurpuricin A (**1**), annopurpuricin B (**2**), annopurpuricin C (**3**), annopurpuricin D (**4**) and annopurpuricin E (**5**).

The antiproliferative effect of **1**–**5** was evaluated on three human tumor cell lines: biphasic mesothelioma (MSTO-211H), cervix adenocarcinoma (HeLa) and hepatocellular carcinoma (HepG2). Moreover, the effect of the most interesting annopurpuricin A (**1**) on the bioenergetic parameters of isolated RLM was investigated. Finally, the occurrence of mitochondrial membrane depolarization and apoptosis on whole cells were analyzed by cytofluorimetric studies.

## 2. Results and Discussion

### 2.1. Isolation and Structure Elucidation

The DCM extract of the roots of *A. purpurea* was fractionated by a chromatography column, employing silica gel 60 Å as the stationary phase to give fractions of substituted lactones. Subsequently, the separation by precipitation with different solvent systems afforded pure compounds **1**–**5**.

A close examination of all compounds showed a similar profile in the spectroscopic data (infrared (IR), ultraviolet–visible spectrophotometry (UV), circular dichroism (CD) and nuclear magnetic resonance [NMR (^1^H and ^13^C)]). IR spectra contained absorptions for hydroxyl (3369 cm^−1^) and α, β-unsaturated lactone (1737–1748 cm^−1^) functionalities. UV λmax absorptions were observed between 208–212 nm [methanol (MeOH)]. The positive Kedde reaction as well as the comparison of ^1^H and ^13^C NMR data of the isolated (**1**–**5**) with those reported for known acetogenins, made evident the presence of the terminal α,β-unsaturated γ-lactone (Table 1 and Table 2) [22]. Compounds **1**–**4** showed four signals in the ^13^C NMR spectra corresponding to the THF oxymethines. They were located between δ 82.1 and 83.3 ppm (parts per million) and correlated with the signals at 3.78 to 9.93 ppm in the ^1^H NMR spectra. A careful analysis of the TMSi (trimethylsilyl) derivatives and the mass spectra revealed that **1**–**4** had a bis-THF ring system. Unlike the others, the correlations observed in HSQC (heteronuclear single-quantum correlation), COSY (correlated spectroscopy) and HMBC (heteronuclear multiple bond correlation) experiments confirmed that **5** instead of THF units possess an epoxide system with an olefinic bond separated by two methylenes.

The absolute configuration of C-36 in annopurpuricins A to E (**1**–**5**) were determined according to Gawroński and Wu [23] by circular dichroism and showed a negative n-π* Cotton effect at 237.5 nm (Δε = -2.0981) and a positive π-π* Cotton effect at 211 nm (Δε = 14.7264), clearly indicating an *S* configuration for all compounds.

The presence of a correlation of the proton at 3.81–3.82 ppm (H-4) with the proton at 2.50–2.51 ppm (H-3b) in the COSY experiment as well as a fragment ion at *m/z* 213 (cleavage between C-4/5) obtained by the tri-TMSi derivative, confirmed the position of the hydroxyl group at C-4 in **2** and **4** [24,25].

#### 2.1.1. Annopurpuricin A (**1**)

**Figure 1:**
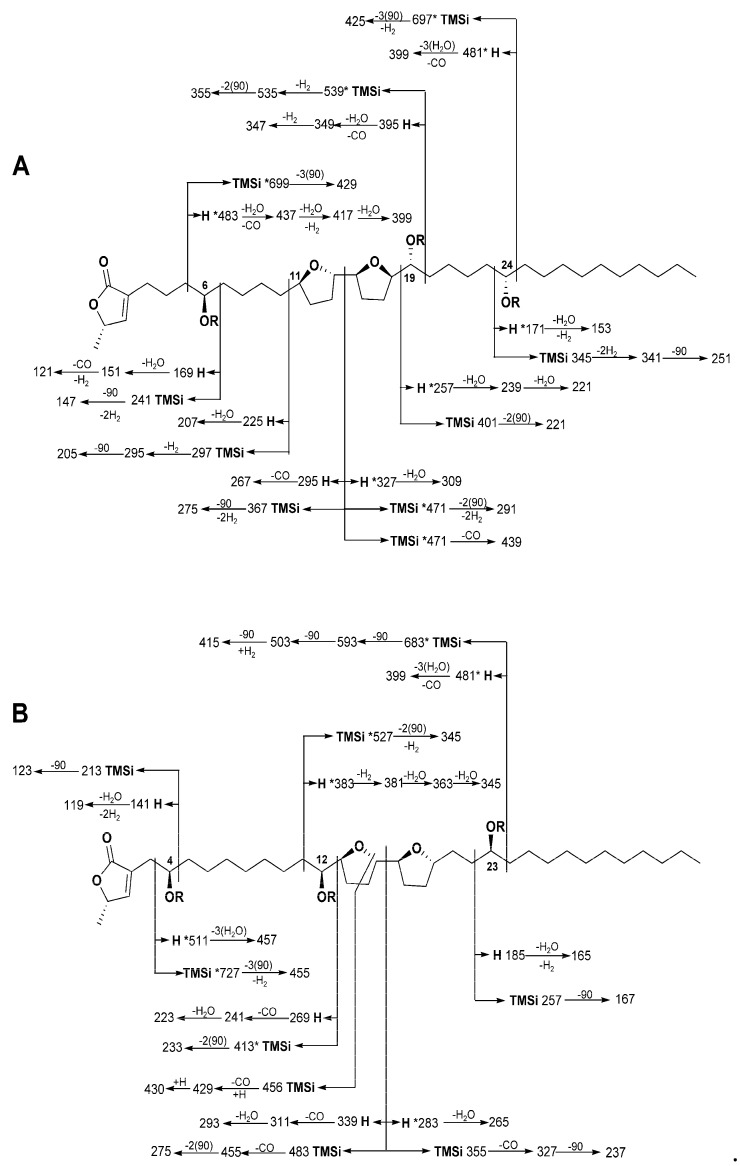
Diagnostic ions in the EI mass spectrum of **1** (**A**) and **2** (**B**). *R* = H (natural compound) and *R* = TMSi [trimethylsilyl derivative (*m/z* 90)]. * Peak not observed.

Compound **1** was obtained as a yellow powder, mp 57–60 °C. The molecular formula was established as C_37_H_66_O_7_ by high-resolution mass spectra (HRMS) using electrospray ionization time-of-flight mass spectrometry (ESI-TOF). The existence of three hydroxyl moieties in the molecule was demonstrated by the detection of three signals at 2.00–2.04 ppm in the ^1^H NMR spectrum of its acetate derivate **1a** (supplementary material). The three hydroxyl moieties appeared in the ^1^H NMR spectrum from δ 3.38 to 3.82 ppm and in the ^13^C NMR at δ 71.4 to 74.1 ppm (Table 1). The position of the hydroxyl groups, as well as the bis-THF unit, was located along the aliphatic chain by the analysis of the fragmentation pattern of the natural compound and its derivative (Figure 1, annopurpuricin A). The first hydroxyl group was situated at C-6 taking into consideration the fragment ions at *m/z* 241 (cleavage between C-6/7) and 429 (*m/z* 699 loses three TMSiO molecules). The second hydroxyl group was located flanking the THF system, according to the COSY and HMBC experiments [δC 74.14 with δH 3.37 (H, C-19)]; as well as the fragment ions at *m/z* 535 (cleavage at C-19/20) and *m/z* 401 (cleavage at C-18/19) in the TMSi mass spectra. The third hydroxyl moiety was placed in C-24 by the presence of the fragment ions at *m/z* 345 and 425, cleavage between C-23/24 and C-24/25 respectively. A COSY experiment using benzene-*d_6_* (C_6_D_6_) as solvent allowed identifying a THF system with only one flanking hydroxyl moiety (3.49/3.85 ppm) (supplementary material). The stereochemistry of the carbinols was determined by employing the methodology of Rieser et al. [26] which includes the generation of the esters of the acid *R* and *S* of Mosher (Table 3). Stereochemistry at C-6, C-19 and C-24 were established as *R, R, S* respectively. The relative stereochemistry around the bis-THF rings was determined as *trans/threo/cis/threo* by a comparison to the literature with the ^1^H and ^13^C NMR data of the compounds rollidecin (C and D), guanaconne and glabracin A [27,28,29].

#### 2.1.2. Annopurpuricin B (**2**)

Compound **2** was isolated as a white amorphous powder. Its molecular formula was established as C_37_H_66_O_7_ by HRMS (ESI-TOF). The signals corresponding to three hydroxyl moieties appeared in the ^1^H NMR spectrum from δH 3.38 to 3.84 ppm and in the ^13^C NMR at δ 70.0 to 74.2 ppm (Table 1).

The position of the hydroxyl moieties and the bis-THF unit were located along the aliphatic chain by the analysis of the fragmentation pattern of the TMSi derivate (Figure 1, annopurpuricin B) as well as an analysis at ^1^H NMR spectra using C_6_D_6_ as solvent (supplementary material). At C-12, a hydroxyl group was placed by the COSY correlation between the protons at 3.37 ppm (H-12) with 3.79 ppm (H-13). The position of the hydroxyl moiety was supported by fragment ions at *m/z* 527 (cleavage at C-11/12) and *m/z* 413 (cleavage at C-12/13). The third hydroxyl group was placed at C-23 by the fragment ions at *m/z* 257 (cleavage between C-22/23), and *m/z* 683 (cleavage between C-23/24, loses three molecules of TMSiO) (Figure 1B) confirmed by the correlation of H-23 with H-22 and H-24 (δ 1.45 and 1.32 ppm) that belong to the aliphatic chain.

The carbinol configurations were determined by a similar method as that used with **1** [26]. The analysis of ^1^H NMR chemical shifts allowed the clear establishment of an *S* stereochemistry of carbinols at C-12 and C-23. The stereochemistry at C-4 was unequivocally assigned as *R*, taking the following arguments into account. First, considering H-35 and H-36 displacements in CDCl_3_, the Δ*δ*(*S*–*R*) values for H-35 and H-36 were −0.25 and −0.05 ppm, respectively, suggesting an unlike relative configuration for C-4/C-36 therefore, if C-36 has the *S* configuration, C-4 must have an *R* configuration [19]. Second, the Δ*δ*(*S*–*R*) values for H-3 and H-5 were -0.06 and +0.02 respectively suggesting once again an *R* stereochemistry. Because of the signal of H-5 in *R* Mosher’s derivative was highly overlapped, additional experiments in C_6_D_6_ were carried out; there, Δ*δ*(*S*–*R*) values for H-3 and H-5 were -0.01 and +0.17 respectively, confirming the proposed stereochemistry (Table 3). As observed by Navrátilová et al. [30] in other carbinols, a comparison of calculations Δ*δ*(*S*–*R*) values using C_6_D_6_ or CDCl_3_ can be useful in the study of stereochemistry. The bis-THF rings stereochemistry was assigned by a comparison with the literature indicating that the relative configurations at the bis-THF system from C-12 to C-20 are *erythro/trans/threo/trans*, similar to guanaconne [27].

#### 2.1.3. Annopurpuricin C (**3**)

**Figure 2:**
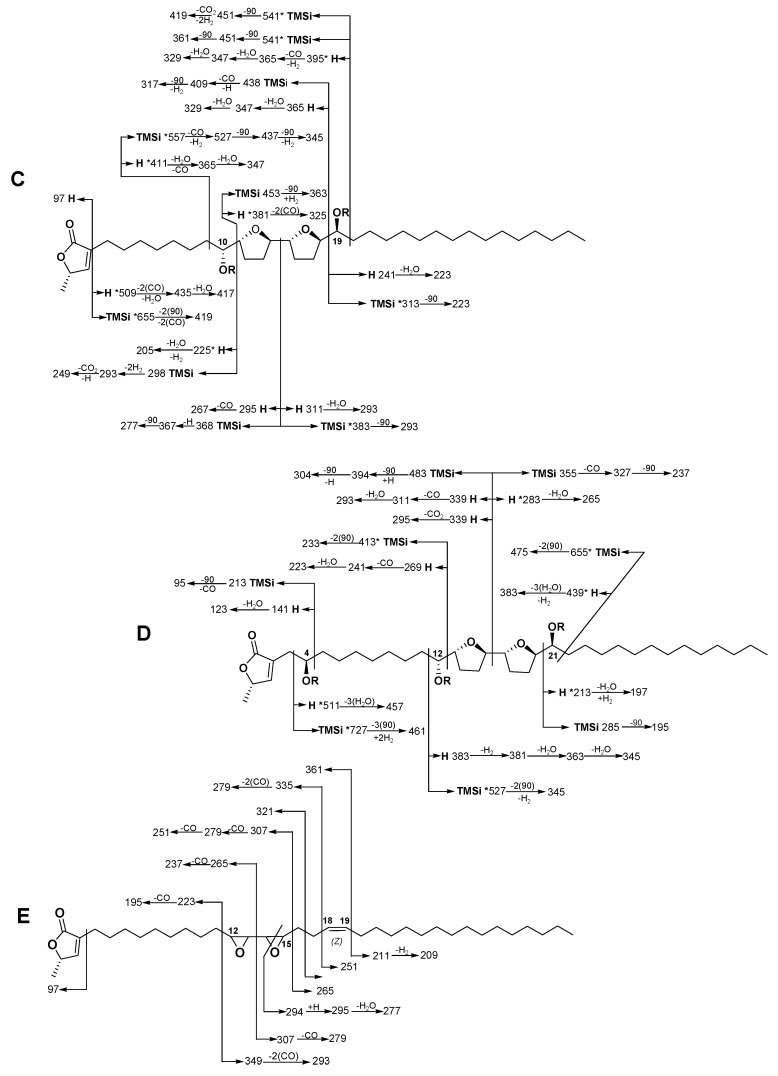
Diagnostic ions in the EI mass spectrum of **3** (**C**), **4** (**D**), **5** (**E**). *R* = H (natural compound) and *R* = TMSi (trimethylsilyl derivative (*m/z* 90)). * Peak not observed.

Compound **3** was isolated as a white powder, mp 69–71 °C. Its molecular formula was established as C_37_H_66_O_6_ by HRMS (ESI-TOF). The ^1^H NMR signals were according to an acetogenin structure (Table 2), as previously discussed, and the presence of two hydroxyls was suggested by the presence of the signals at δ 3.39 and 3.88 ppm, and at δ 71.4 and 74.2 ppm in the ^13^C NMR, which was in agreement with the presence of two signals at δH 2.05 and 2.07 ppm in the ^1^H NMR spectrum of the acetate derivative **3a**. A detailed analysis, using C_6_D_6_ as solvent, (supplementary material) was made to prevent overlapping signals and to establish the presence of a bis-THF system with two flanking hydroxyls. In addition, the position of the system was established by the analysis of the fragmentation pattern (Figure 2, annopurpuricin C) were hydroxyl groups were located at C-10 and C-19, and the bis-THF unit at C-11 to C-18. The stereochemistry of the carbinols was determined by employing the same methods for compounds **1** and **2**. The NMR chemical shifts of the protons close to C-10 and C-19 in the Mosher derivatives are reported in Table 3; for both carbinols, the configurations were established as *R* and *S* respectively [31]. A comparison with the literature of chemical shifts of compound **3** indicated a relative stereochemistry between C-10 to C-19 as *erythro/trans/threo/trans/erythro*, similar to atemotetrolin and espelicin [24,25,32].

#### 2.1.4. Annopurpuricin D (**4**)

Compound **4** was obtained as a white powder, mp 77–78 °C. Its molecular formula was established as C_37_H_66_O_7_ by HRMS (ESI-TOF). The analysis of its acetate derivative (**4a**) allowed the detection of three signals from 2.03 to 2.05 ppm in the ^1^H NMR spectrum, suggesting the presence of three hydroxyl groups (supplementary material). The position of the hydroxyl groups, as well as the bis-THF unit, were located along the aliphatic chain by the analysis of the fragmentation patterns obtained from the mass spectrometry of the underivatized material and the TMSi derivative (Figure 2, annopurpuricin D). The first hydroxyl group was located at C-4 by the observation of a signal at 70 ppm in the NMR and the correlation between the proton at 3.82 ppm with the signal at 2.50 (H-3b) in the COSY experiment [24,25]. The position in C-4 was confirmed by the fragment ions at *m/z* 213 (cleavage between C-4/5) and *m/z* 461 (cleavage between C-3/4). The last two hydroxyl groups were assigned at C-12 and C-21, due to the correlations detected in the COSY and HMBC experiments employing CDCl_3_ as solvent. Further, independent experiments using C_6_D_6_ as solvent were carried out. Additional information was obtained by analyzing the mass spectra of the TMSi derivative (Figure 2, annopurpuricin D). The relative configuration of the THF system was established as *erythro/trans/threo/trans/erythro*, like rollitacin [33,34], purpurediolin and purpurenin [20]. Carbinol configurations in **4** were established as C-4*R*, C-12*R*, C-21*S* (Table 3) by the same methods [26].

#### 2.1.5. Annopurpuricin E (**5**)

Compound **5** was isolated as a white powder, mp 55–56 °C. Its molecular formula was established as C_37_H_64_O_4_ by HRMS (ESI-TOF). The presence of an isolated extra double bond in **5** was suggested by a proton multiplet at δ 5.39 (2H) in the ^1^H NMR spectrum and two carbon resonances at δ 128.2 and 131.4 in the ^13^C NMR spectra [33,35,36,37]. The ^13^C NMR chemical shifts of the α-methylene carbons were δ 23.4 and δ 27.2 ppm indicating a *Z* stereochemistry, (Table 2) [36,37]. The *cis* configuration of the double bond of **5** was also suggested by a comparison of the chemical displacement with those linear acetogenins reported in the literature [33,35,36,37]. The position of the double bond at C-18/19 was separated by two methylens from an epoxide system by the fragment ion at *m/z* 335 (cleavage of C-17/18) (Figure 2C). This system was confirmed by the correlation between δH 2.95 to 2.97 ppm (four protons) and δC 56.9- 57.3 ppm [38]. Its position (C12/15) was determined by the fragment ions at *m/z* 307 and 265 (cleavage of C-13/14) (supplementary material).

In addition to the chemical and spectroscopy analysis, a comparison of experimental and calculated vibrational frequencies (cm^−1^) for all annopurpuricins was carried out (see *Computational details*) [39]. The experimental values were in agreement with those predicted for the proposed structures **1**–**5** (Tables S13, S26, S39, S52, S62 in supplementary material).

### 2.2. Antiproliferative Activity

The ability of annopurpuricins A–E (**1**–**5**) to inhibit cell growth was evaluated by means of an in vitro assay performed on three human tumor cell lines: MSTO-211H (biphasic mesothelioma), HeLa (cervix adenocarcinoma) and HepG2 (liver hepatocellular carcinoma). The results, expressed as GI_50_ values, i.e., the concentration (nM) of compound able to produce 50% cell death with respect to the control culture, are shown in Table 4. Camptothecin, a natural product with well-known antiproliferative activity, was used as the reference compound.

The obtained results clearly indicate a notable antiproliferative activity for acetogenins **1**–**4** showing GI_50_ values ranging from 0.06 to 25.9 nM. In particular, annopurpuricin A (**1**) is the most active compound on both HeLa and HepG2 cells, being 90 and 6.6 times more potent than camptothecin, respectively. Otherwise, compound **5** does not exert any cytotoxicity in the same experimental conditions.

Taking into consideration the acetogenins endowed with antiproliferative activity (**1**–**4**), it is possible to note a quite different sensitivity between the different cell lines, with the highest antiproliferative effect on HeLa cells and the lowest on MSTO-211-H. In particular, the GI_50_ values obtained for HeLa cells are from about 17 (compound **3**) to about 400 (compound **1**) times lower with respect to those of MSTO-211H. With respect to Hep-G2, these cells show an intermediate sensitivity with GI_50_ values ranging from 0.45 to 2.5 nM. By considering the most sensitive HeLa and Hep-G2 cell lines, a general behavior can be noted which highlights acetogenin **1** as the most active, and **3** as the least cytotoxic.

In this connection, taking into consideration the structures depicted in Figure 1 and Figure 2 and the results shown in Table 4, some qualitative structure-activity relationships can be drawn. In particular, the lack of cytotoxicity by acetogenin **5**, could suggest a role for the THF rings, the saturated side chains and the hydroxyl substituents in the biological activity of **1**–**4**. In addition, the relative length of aliphatic chains does not seem determinant for the antiproliferative effect. Indeed, **1** and **3**, as well **2** and **4**, notwithstanding the significant differences in biological activity, are characterized by the same number of carbon atoms in the aliphatic chains. Similarly, the total number of hydroxyl groups does not appear related with cytotoxicity, because the effect exerted by **3** is not significantly different to those of **4** that possesses, along with two hydroxyl groups flanking the THF rings, a further hydroxyl moiety at C-4 position.

**Figure 3:**
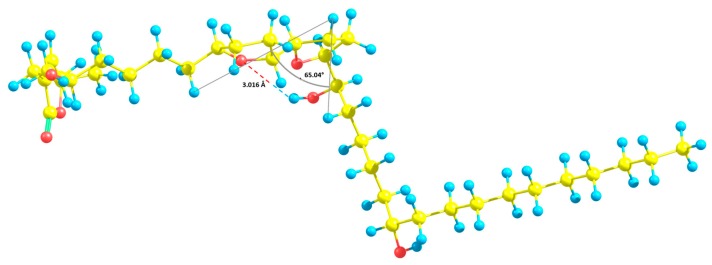
Molecular model of annopurpuricin A (**1**).

Finally, it has to be underlined that annopurpuricins A-D (**1**–**4**) had a relative configuration *trans*/*threo* between both THF rings, similar to bullatacin, an acetogenin characterized by notable antiproliferative activity [10,11]. Moreover, the molecular model of annopurpuricin A (Figure 3) highlighted structural attributes in agreement with a recent theoretical study which correlates very strong O-H stretching intensities, characteristic of hydrogen bonding, with bent *V*-type equilibrium geometry structures [41]. Furthermore, comparisons of the 3D-structures of highly bioactive acetogenins seem to indicate that this special feature could improve the interactions with the target.

The higher cytotoxic effect of **1** with respect to the other isolated acetogenins (**2**–**5**) on both HeLa and HepG2 cells prompts us to investigate the mechanism of action responsible for such an interesting biological profile. In particular, on the basis of previous studies demonstrating the ability of acetogenin derivatives to inhibit mitochondrial complex I [13,15], the effect of **1** on the bioenergetic parameters in isolated RLM was studied.

### 2.3. Effect on Mitochondrial Membrane Potential

To evaluate the bioenergetic parameters on isolated RLM, the most commonly utilized substrate is succinate (succ) in the presence of rotenone (rot). This is because succ energizes mitochondria by its oxidizing enzyme, succinate dehydrogenase, which is a component of the respiratory chain, the so-called complex II. Rot is an inhibitor of NADH dehydrogenase (complex I), and consequently it inhibits the oxidation of all the NAD-dependent substrates, so emphasizing the oxidation of succ.

**Figure 4:**
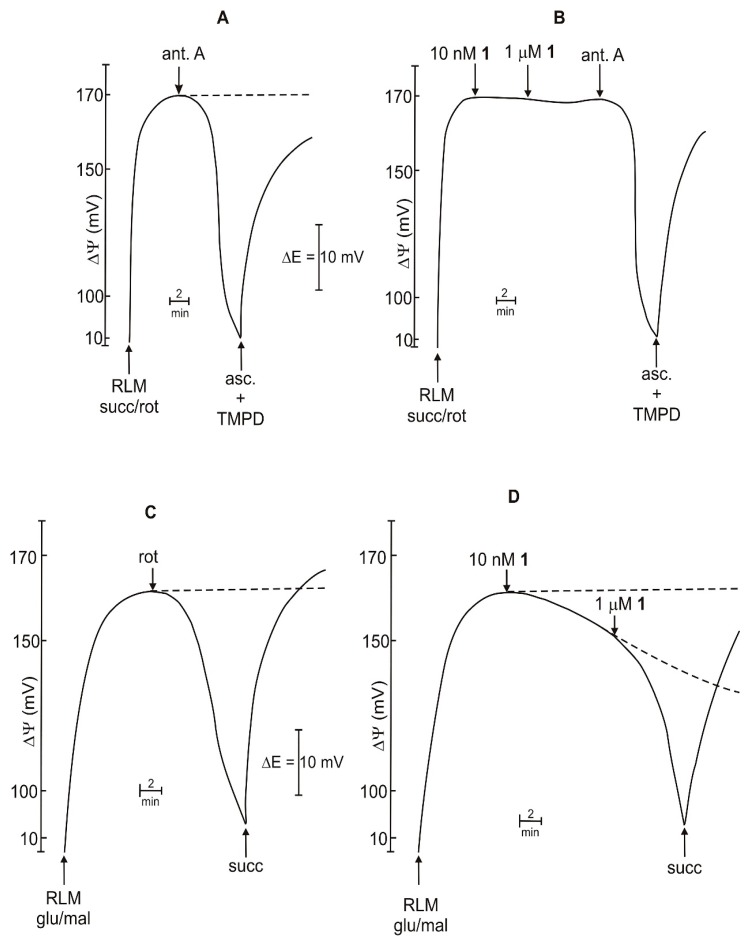
Effects of compound **1** on mitochondrial ΔΨ. (**A**,**B**): RLM (1 mg/mL) are incubated in a medium containing succ (5 mM) and rot (1.25 µM). (**C**,**D**): RLM (1 mg/mL) are incubated in a medium containing glu (5 mM) and mal (1 mM). Test compound was added at indicated concentrations. When present 10 mM asc, 100 μM TMPD, 1.25 μM rot,1 μM ant A. Dotted lines represent the behavior of mitochondrial ΔΨ without the addition indicated by the arrow.

In Figure 4A,B a typical bioenergetic experiment showing the mitochondrial membrane potential (ΔΨ) establishment by oxidation of succ plus rot is reported, along with the effect of compound **1**. As it is observable in Figure 4A, RLM incubated in a standard medium and energized as above mentioned, exhibit a ΔΨ of about 170 mV that is maintained for a long time (dotted line). The addition of antimycin A (ant A), an inhibitor of complex III, rapidly collapses ΔΨ, while a subsequent addition of ascorbate (asc) plus the shuttle tetramethyl-*p*-phenylendiamine (TMPD), that transfers electrons to cytochrome C, restores it. These results demonstrate in the isolated RLM the optimal functioning of the segment of the respiratory chain from complex II to oxygen.

Annopurpuricin A (**1**) is ineffective in inducing any change in ΔΨ at 10 nM concentration and after a further addition of 1 µM (Figure 4B). Addition of ant A, followed by asc/TMPD, exhibits the same effects as in Figure 4A, thus demonstrating that **1** does not alter the membrane integrity and the activity of the proton pumps, and electron transport located on the respiratory chain, downstream complex II.

Figure 4C shows the ΔΨ obtained in the presence of glutamate (glu)/malate (mal) as energizing substrates, that is a more physiological condition with respect to that in Figure 4A,B. Glu is transported in mitochondria by the activity of the shuttle malate/aspartate, then it is oxidized to α-ketoglutarate by glutamate dehydrogenase with the formation of NADH. The latter transfers electrons to NADH dehydrogenase, the complex I of the respiratory chain. This more complicated pathway causes a slower respiration and a consequent lowering in ΔΨ. Actually, mitochondrial ΔΨ established by the oxidation of glu/mal exhibits a value of about 160 mV (Figure 4C, dotted line). As expected, the addition of rot, an inhibitor of NADH dehydrogenase, induces a drop in ΔΨ, while a subsequent addition of succ restores it at the maximum value (Figure 4C, continuous line). The addition of 10 nM, **1** induces a gradual drop in ΔΨ, which becomes almost completely collapsed at 1 μM concentration (Figure 4D, continuous line). A subsequent addition of succ is able to restore ΔΨ. Overall, the results reported in Figure 4C,D suggest for **1** a rotenone-like effect.

### 2.4. Mitochondrial Respiration Parameters

**Figure 5:**
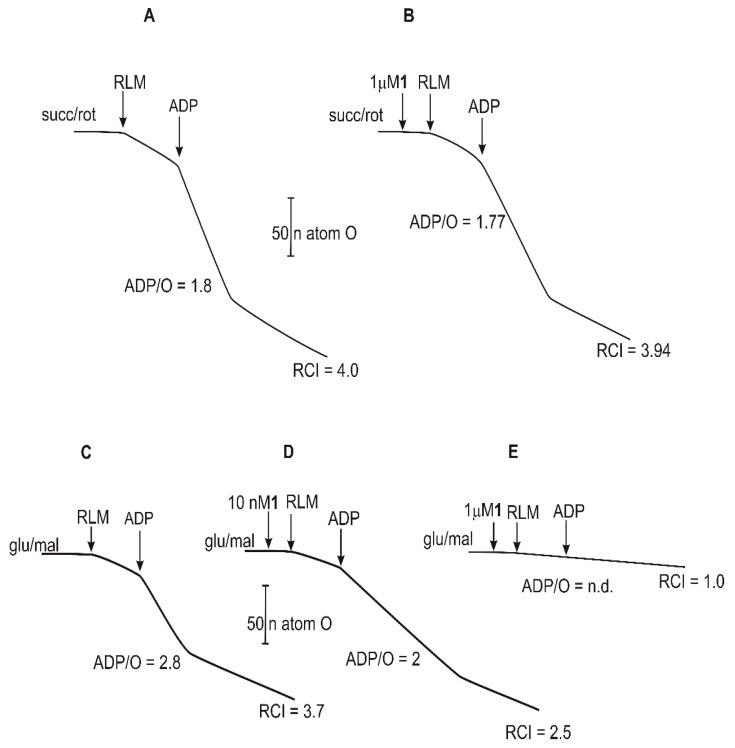
Effects of compound **1** on RCI and ADP/O ratio. (**A**,**B**): RLM (1 mg/mL) were incubated in a medium containing succ (5 mM) and rot (1.25 µM). (**C**–**E**): RLM (1 mg/mL) were incubated in a medium containing glu (5 mM) and mal (1 mM). Test compound was added at indicated concentrations. ADP concentration was 0.2 mM.

Other bioenergetic parameters that characterize mitochondrial functions, and also demonstrate the coupling of mitochondrial respiration with oxidative phosphorylation, are the respiratory control index (RCI) and the ADP (adenosine diphosphate)/O ratio. The results presented in Figure 5A show the calculation of these parameters in RLM energized by succ/rot. As observable in this Figure RCI exhibits a value of about 4 and ADP/O ratio of about 1.8, which are values typical of good mitochondrial preparations. The RCI and ADP/O ratio, calculated in the presence of **1** (1 μM) (Figure 5B), show values of about 3.94 and 1.77, respectively, and this means that the compound exhibits an almost negligible effect on these parameters. If RLM are energized by glu/mal, RCI and ADP/O ratio are about 3.7 and 2.8, respectively (Figure 5C), values in this case typical for these substrates. It is evidenced that both state 4 and state 3 respiration are slightly slower than those observed with succ/rot (compare Figure 5C with Figure 5A), in accordance with the above explanation. The addition of **1** at 10 nM, at RLM incubated in the presence of glu/mal, inhibited respiration states 4 and 3, with a consequent significant reduction of RCI and ADP/O ratio to 2.5 and 2, respectively (Figure 5D).

In these experimental conditions, the addition of **1** at 1 μM concentration almost completely blocks the oxygen consumption in both the respiratory states, and RCI becomes 1 (the lowest value) while ADP/O ratio is not detectable (Figure 5E). These results clearly indicate that **1** induces a dose-dependent inhibition of complex I of the respiratory chain, most probably by a rotenone-like effect, in accordance with previous studies on a number of acetogenins and derivatives [7,13,15].

**Figure 6:**
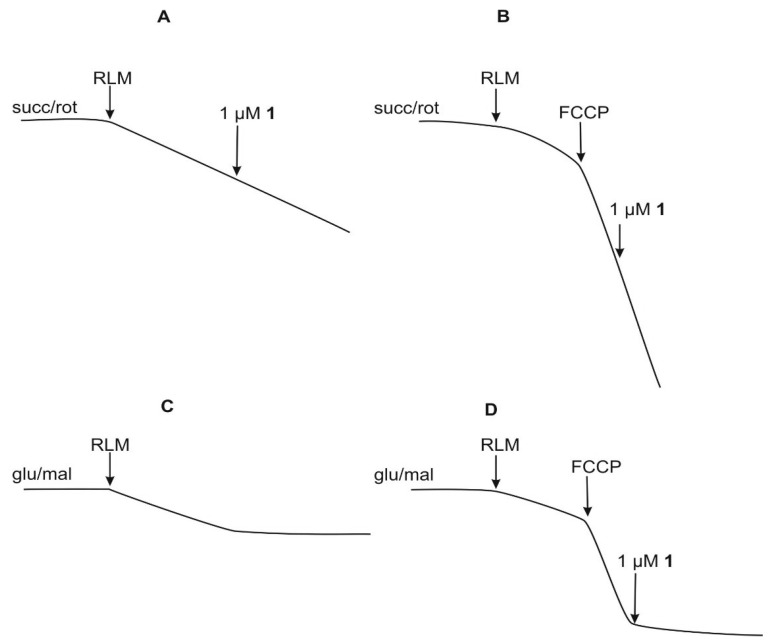
Effects of compound **1** on oxygen consumption in the absence or in the presence of the inhibitor of phosphorylation FCCP. (**A**,**B**): RLM (1 mg/mL) were incubated in a medium containing succ (5 mM) and rot (1.25 µM). (**C**,**D**): RLM (1 mg/mL) were incubated in a medium containing glut (5 mM) and mal (1 mM). Test compound was added at indicated concentration. FCCP concentration was 0.1 μg/mg protein.

Figure 6 shows the respiration of RLM in state 4 (A) and in uncoupled states induced by carbonylcyanide-*p*-trifluoromethoxyphenylhydrazone (FCCP) (B) in RLM energized with succ/rot. The addition of **1** at 1 μM is completely ineffective on oxygen consumption in both cases, by confirming that **1** does not inhibit the electron transport downstream complex I, also at different rates of this transport. Otherwise, with glut/mal (Figure 6C,D), the test compound at 1 μM completely blocks oxygen consumption both in state 4 (C) and in uncoupled state (D), thus confirming the block of complex I activity.

### 2.5. Mitochondrial Permeability Transition

As it is well known, mitochondria incubated in energized conditions, and in the presence of high Ca^2+^ concentrations, undergo the so-called mitochondrial permeability transition (MPT) with the opening of the transition pore [42]. This event causes a bidirectional traffic of solutes and metabolites across the mitochondrial membranes leading to the collapse of the mitochondrial gradients, matrix swelling and the release of proapoptotic factors [43].

**Figure 7:**
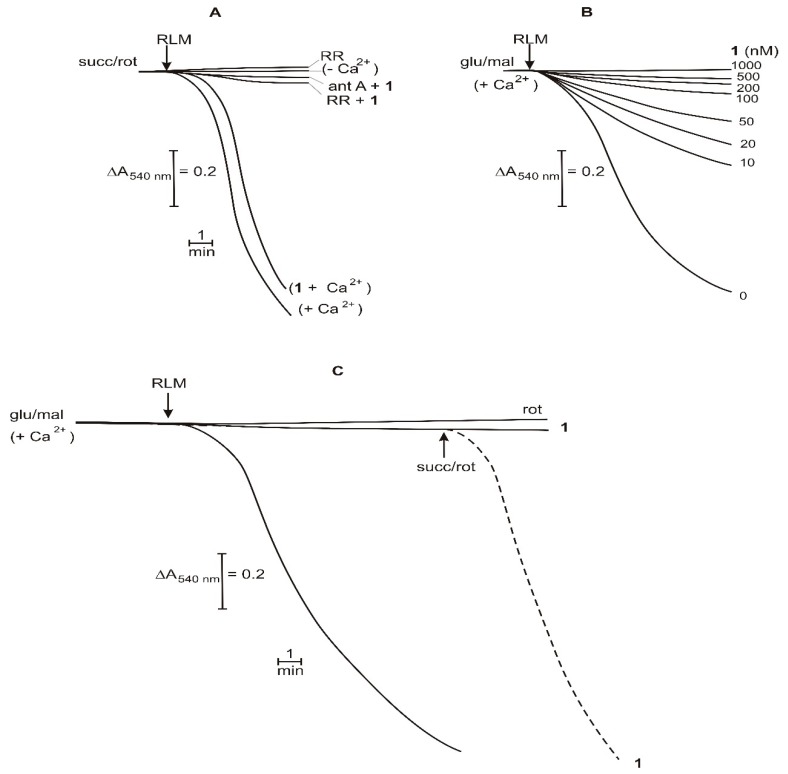
Effects of compound **1** on mitochondrial swelling. (**A**): RLM (1 mg/mL) were incubated in a medium containing succ (5 mM) and rot (1.25 µM). (**B**,**C**): RLM (1 mg/mL) were incubated in a medium containing glu (5 mM) and mal (1 mM). Test compound was added at 1 μM (**A**,**C**) or at indicated concentrations (**B**). Ca^2+^ was present or added at 20 μM. Dotted line represents the behavior of mitochondrial swelling without the addition indicated by the arrow.

The induction of MPT is generally detected by measuring mitochondrial swelling. The results of Figure 7A show that RLM, energized by succ/rot and treated with 100 μM Ca^2+^, undergo a large amplitude swelling detected by a decrease in the apparent absorbance of mitochondrial suspension measured at 540 nm (+Ca^2+^). This event does not occur in the absence of Ca^2+^ (-Ca^2+^) or in the presence of the inhibitor of Ca^2+^ transport, ruthenium red (RR), thus evidencing the need of Ca^2+^ for inducing MPT. It is also evident that for inducing this phenomenon, Ca^2+^ has to enter into mitochondria by its electrophoretic uniport and consequently, the organelles have to be energized. This is confirmed by the results obtained with complex I inhibitor, ant A, that indeed completely prevents mitochondrial swelling. If **1** is added before Ca^2+^, at concentrations of 10 nM or 1 μM, in the above condition (succ/rot), the mitochondrial swelling is not affected, suggesting that Ca^2+^ transport is normally operating in the presence of **1** (results not shown). It is also evidence that both RR and ant A block swelling also in the presence of **1**.

RLM energized by glu/mal and treated with Ca^2+^ also exhibit mitochondrial swelling without any significant change with respect to the above experimental conditions (Figure 7B). Nevertheless, if different concentrations of **1** are added, a dose-dependent inhibition of mitochondrial swelling occurs.

Interestingly, both rot and compound **1** at 1 μM concentration, completely inhibit the swelling of RLM energized by glu/mal (Figure 7C). Moreover, the inhibition of swelling exerted by **1** is completely removed by the addition of succ/rot, and this result demonstrates that rot and **1** exhibit an almost identical inhibitory effect. The restoration of the energizing condition by succ/rot addition, in turn, restores mitochondrial swelling (Figure 7C).

### 2.6. Cytofluorimetric Analysis

**Figure 8:**
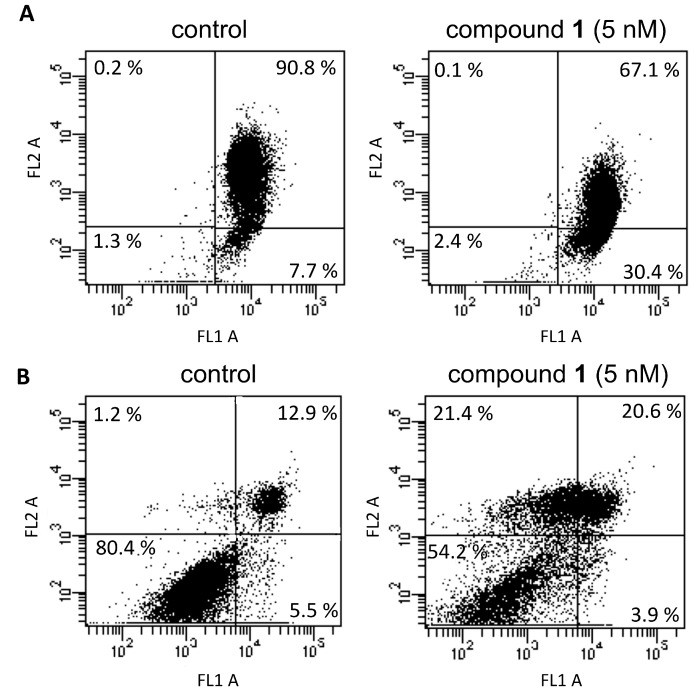
Effect of **1** on (**A**) mitochondrial ΔΨ of cells stained with JC-1 or (**B**) cell death mechanism. Dot plots from the flow cytometry analysis performed on HeLa cells incubated for 28 h in the absence (control) or in the presence of 5 nM compound **1**. A representative experiment of at least three replicates is reported.

To prove the involvement of mitochondrial damage on the cell effects induced by annopurpuricin A (**1**), the mitochondrial functionality in the presence of test compound was evaluated in whole HeLa cells by using JC-1 (5,5′,6,6′-tetrachloro-1,1′,3,3′-tetraethylbenzimidazolocarbo-cyanine iodide). The high mitochondrial ∆Ψ, negative inside, drives the uptake of this cationic probe into the matrix, leading to a highly red fluorescent emission. Following the collapse of mitochondrial ΔΨ, JC-1 leaks out from the organelles into the cytoplasm, resulting in a decrease of red fluorescence. The presence of **1** at 5 nM concentration induces a significant decrease of red fluorescence in cells with mitochondria labeled with JC-1 (Figure 8A). In particular, the percentage of cells showing depolarized mitochondria rises from 7.7% in untreated cells (control) to 30.4% in cells treated with **1**.

The result reporting that **1** is not able to induce MPT by preventing Ca^2+^ transport can be considered in the light of the triggering of intrinsic apoptosis at a cellular level. Nevertheless, if a block of the MPT induction can lead to a prevention of apoptosis, a block of complex I activity can result in a gradual deenergization of the cell, when the supply of FADH_2_ [flavin adenine dinucleotide (reduced form)] comes to lack, even though complex II should be operating, that can lead to cell death by necrosis. The investigation on the mechanism of cell death was performed by incubating HeLa cells in the presence of **1** at 5 nM concentration for 28 h. The obtained results highlighted a decrement of cell viability from 80.4% (untreated cells, control) to 54.2% in the presence of test compound (Figure 8B). Interestingly, annopurpuricin A (**1**) induces a significant increase of cells in late apoptosis (from 12.9% to 20.6%) and, more notably, an increase of necrotic cells from 1.2 % to 21.4%.

## 3. Materials and Methods

### 3.1. General Experimental Procedures

Melting points were obtained in a Fisher Johns fusiometer. Optical rotations were recorded in methanol on a JASCO P-2000 polarimeter. UV data were obtained on an Evolution 300 spectrophotometer in MeOH solution. Circular Dichroism spectra were performed on a JASCO 810 spectropolarimeter at 25 °C in MeOH solution. FTIR spectroscopic data were measured on a Varian 660-IR spectrophotometer. NMR spectra were recorded in a Bruker 400 MHz using CDCl_3_ and C_6_D_6_ as solvents. The chemical shifts are given in δ (ppm) and coupling constants (*J*) are reported in Hz. ESI mass analysis was carried out employing a direct infusion in a triple quadrupole mass spectrometer Xevo TQD (Waters, Milford, MA, USA). The fragmentation patterns were obtained under conditions of capillary voltage: 2.5, 3.0 and 3.2 kV, cone voltage: 20, 50 and 120 V, collision energy: 10 to 35 V; a source temperature of 300 °C, cone gas flow 40 L/h and desolvation gas flow was adjusted to 600 L/h. The source parameters were established in positive ion mode (ESI^+^) with a sample flow of 10 μL/min. The EI-MS analysis of trimethylsilyl derivatives was made in a Varian 3800 Gas Chromatograph equipment coupled with Varian Saturn 2200 Ion Trap Mass Spectrometer. The flow were set at 1 mL/min with a temperature range from 100 °C to 280 °C. High-resolution mass spectra were acquired with an Agilent Technologies ESI-TOF spectrometer. Additional elemental analyses were performed on a Truspec^®^ Micro Analyzer. The purity of each isolate was determined by HPLC analysis performed on a Waters e2695 coupled with a photodiode array detection 2998, using an analytical column XBRIDGE (3.5 μm, 4.6 × 150 mm, C_18_), the mobile phase was MeOH/water (90:10), with a flow of 1 mL/min. The detector was set at 210 nm. The diode array detector was set at 200–600 nm. The Empower 3 software program (Waters) performed control of the equipment, data acquisition, processing and management of chromatographic information.

### 3.2. Plant Material

The roots of *Annona purpurea* Moc & Sessé ex Dunal were collected in January 2014, in Minatitlán, Colima, México, (19°21′24.4″ N, 104°05′01.6″). The plant was correctly identified at the “Herbario Nacional de México” (MEXU) of the “Universidad Nacional Autónoma de México (UNAM)” and a voucher specimen was deposited (MEXU 1457308). An additional collection from Santa María, Michoacán, Mexico was also used (MEXU 1054418) to compare the presence of the metabolites in a different area (data not included). Considering the importance of the root in the survival of a tree, the sample taken by individual was an amount that did not represent risk, and after the collection a continuous monitoring of the survival of trees was made.

### 3.3. Extraction and Isolation

Roots of *A. purpurea* (3.47 Kg) were cleaned, dried and ground. Then, they were exhaustively extracted with hexane, DCM and methanol by maceration at room temperature (24 h × 5 L) to give three extracts, hexane (10.2 g), DCM (20.1 g) and methanol (103.5 g). The extracts were analyzed by TLC. Kedde´s reagent was employed to detect the presence of lactones in the DCM extract. Separation of the compounds present in the DCM extract was carried out using a chromatographic column of 4Φ × 60 cm packed with silica gel for thin layer chromatography (Macherey-Nagel). The column was loaded with 13 g of the extract, and it was eluted stepwise with solutions of n-hexane: ethyl acetate: methanol of increasing polarity. During the elution procedure, 210 fractions of 25 mL volume were obtained. The composition of the eluent solutions and their total volumes where: 100:0:0 (250 mL, fractions 1–10), 90:10:0 (500 mL, fractions 11–30), 80:20:0 (750 mL, fractions 31–60), 70:30:0 (750 mL, fractions 61–90), 50:50:0 (1000 mL, fractions 91–130), 20:80:0 (750 mL, fractions 131–160), 0:100:0 (1400 mL, fractions 161–200), 0:80:20 (125 mL, fractions 201–205), 0:0:100 (125 mL, fractions 206–210). These fractions were grouped in 27 pools (A-Z) according to the TLC profile, of which the most abundant fractions were I (fractions 100–115), R (fractions 131–143), W (fractions (147–160), X (fractions 167–170), Z (172–202); showing a positive reaction to Kedde’s reagent. These pools were purified to afford compounds **1** to **5**. Compound **1** (500 mg) was obtained from fraction Z by precipitation with a cold mixture of hexane: acetonitrile (1:1). **2** (30 mg) and **4** (268 mg) were obtained by a similar method employing the fractions W and X, respectively. Treatment of the fraction R with a heterogeneous system of methanol and hexane (95:5) yielded the purification of a white solid **3** (209 mg). Compound **5** was obtained from fraction I by treatment with methanol until a white wax was obtained; this was subsequently precipitated employing a mixture of hexane and acetonitrile (1:2) to yield 185.8 mg.

#### 3.3.1. Annopurpuricin A (**1**)

Yellow amorphous powder; 0.018% (500 mg); mp 57–60 °C; [α]^25^_D_ +0.34° (*c* 0.02, MeOH); CD (MeOH) ∆ε (nm): −2.09 × 10^3^ (237.5); UV (MeOH) λmax (log ε) 209 (4.00); IR νmax 3369, 2916, 2850, 1742, 1471, 1324, 1080, and 869 cm^−1^; HRMS (ESI-TOF) *m/z*: 645.4695 [C_37_H_66_O_7_ +Na]^+^, calcd. 645.4701 [C_37_H_66_O_7_Na]^+^, calcd. 622.4809 [C_37_H_66_O_7_]. ^1^H and ^13^C NMR spectroscopic data, see Table 1.

#### 3.3.2. Annopurpuricin B (**2**)

White amorphous powder; 1.30 × 10^−3^% (30 mg); mp 65–66 °C; [α]^25^_D_ −0.11° (*c* 0.02, MeOH); CD (MeOH) ∆ε (nm): −1.81 × 10^3^ (237.5); UV (MeOH) λmax (log ε) 212 (4.00); IR νmax 3414, 3370, 2916, 2849, 1737, 1471, 1072 cm^−1^; HRMS (ESI-TOF) *m/z*: 645.4700 [C_37_H_66_O_7_ +Na]^+^, calcd. 645.4701 [C_37_H_66_O_7_Na]^+^, calcd. 622.4809 [C_37_H_66_O_7_]. ^1^H and ^13^C NMR spectroscopic data, see Table 1.

#### 3.3.3. Annopurpuricin C (**3**)

White amorphous powder; 6.65 × 10^−3^% (209 mg); mp 69–71 °C; [α]^25^_D_ +0.44° (*c* 0.02, MeOH); CD (MeOH) ∆ε (nm): −2.16 × 10^3^ (237.5); UV (MeOH) λmax (log ε) 209 (4.00); IR νmax 3414, 2915, 2849, 1743, 1471, 1070 cm^−1^; HRMS (ESI-TOF) *m/z*: 629.4753 [C_37_H_66_O_6_ +Na]^+^, calcd. 629.4752 [C_37_H_66_O_6_Na]^+^, calcd. 606.4859 [C_37_H_66_O_6_]. ^1^H and ^13^C NMR spectroscopic data, see Table 2.

#### 3.3.4. Annopurpuricin D (**4**)

White amorphous powder; 8.1 × 10^−3^% (268 mg); mp 77–78 °C; [α]^25^_D_ −0.27° (*c* 0.02, MeOH); CD (MeOH) ∆ε (nm): −1.67 × 10^3^ (238.5); UV (MeOH) λmax (log ε) 210 (3.94); IR νmax 3413, 2917, 2850, 1740, 1072 and 1025 cm^−1^; HRMS (ESI-TOF) *m/z*: 645.4706 [C_37_H_66_O_7_ +Na]^+^, calcd. 645.4701 [C_37_H_66_O_7_Na]^+^, calcd. 622.4809 [C_37_H_66_O_7_]. ^1^H and ^13^C NMR spectroscopic data, see Table 2.

#### 3.3.5. Annopurpuricin E (**5**)

White amorphous powder; 5.63 × 10^−3^% (185.8 mg); mp 55–56 °C; [α]^25^_D_ −0.43° (*c* 0.02, MeOH); CD (MeOH) ∆ε (nm): −1.68 × 10^3^ (239); UV (MeOH) λmax (log ε) 208 (3.85); IR νmax 2950, 2916, 2848, 1748, 1462, 831 cm^−1^; HRMS (ESI-TOF) *m/z*: 595.4693 [C_37_H_64_O_4_+Na]^+^, calcd. 595.4697 [C_37_H_64_O_4_Na^]+^, calcd. 572.4804 [C_37_H_64_O_4_]. ^1^H and ^13^C NMR spectroscopic data, see Table 2.

### 3.4. Preparation of Derivatives

#### 3.4.1. Acetyl derivatives (**1a–4a**)

Treatment of **1**–**4** (10 mg) with acetic anhydride/pyridine (room temperature, 18 h) and subsequent work-up gave compounds **1a–4a** (13–20 mg of yield) as a yellow semisolid [20].

#### 3.4.2. Trimethylsilyl Derivative

A 1 mg sample of each dry compound (**1**–**4**) was treated with N,O-Bis (trimethylsilyl) trifluoroacetamide (BSTFA) (Sigma Chemical Co., St. Louis, MO, USA) and dry pyridine (10: 1) at room temperature for 30 min. The derivatization conditions were applied following the conditions given by the supplier with slight modifications to obtain the analytical response [44].

#### 3.4.3. (*R*) - and (*S*)-MTPA Ester Derivatives

In a 5 mL round-bottom flask, 5 mg of the selected acetogenin (**1**–**4**) was dissolved in 1 mL of CH_2_Cl_2_, after that, 36 mg of the (*R*)-(+)-α-Methoxy-α-trifluoromethylphenylacetic acid [(*R*)-MTPA] were added. The reaction was mixed with a magnetic stir bar and placed on ice bath, under a N_2_ gas flow. Subsequently, 30 mg of DCC and 5 mg of DMAP were added. The reaction system was carefully shaken in an ice bath for 5 min, and then at room temperature. After that dicyclohexylurea precipitated. Each reaction was monitored by TLC and dried under vacuum pressure once the reaction had finished. Then, the residue was percolated using Si-gel and employing an ascending polarity system as eluent. Finally, the product was dissolved in CDCl_3_ and analyzed by ^1^H NMR spectroscopy. The (*S*)-MTPA ester of each acetogenin was prepared from (*S*)-MTPA [(S)-(−)-α-methoxy-α-(trifluoromethyl)phenylacetic acid] following the same method [26]. All reactive were purchased by (Sigma Chemical Co., St. Louis, MO, USA).

### 3.5. Computational Details

Geometry optimizations of the acetogenins, without symmetry restrictions, were performed within the density functional theory framework implemented in the Gaussian 09 [45] code. The hybrid functional B3LYP [46] and wB97XD which uses a version of Grimme’s D2 dispersion model [47], were employed together with the all-electron 6-311G(d, p) basis set. The minimum energy states were verified through a vibrational analysis at the same theory level and compared with experimental FTIR data of each acetogenin. The harmonic frequencies were scaled depending on the function, namely, 0.9614 value for B3LYP functional as recommended by Scott and Random [48] and 0.957 value for the other functional as suggested by the Computational Chemistry Comparison and Benchmark DataBase [49].

### 3.6. Inhibition Growth Assay

MSTO-211H and HeLa were grown in RPMI 1640 (Sigma Chemical Co., St. Louis, MO, USA) supplemented with 2.4 g/L HEPES (2-[4-(2-hydroxyethyl)piperazin-1-yl]ethanesulfonic acid), 0.11 g/L pyruvate sodium and Nutrient Mixture F-12 [HAM] (Sigma Chemical Co., St. Louis, MO USA), respectively. HepG2 were grown in Dulbecco’s Modified Eagle’s Medium (Sigma Chemical Co., St. Louis, MO, USA). 10% Heat-inactivated fetal bovine serum (Invitrogen, Carlsbad, CA, USA), 100 U/mL penicillin, 100 µg/mL streptomycin, and 0.25 μg/mL amphotericin B (Sigma Chemical Co., St. Louis, MO, USA) were added to all media. The cells were cultured at 37 °C in a moist atmosphere of 5% carbon dioxide in air. Cells (3 × 10^4^) were seeded into each well of a 24-well cell culture plate. After incubation for 24 h, various concentrations of the test acetogenins were added. The cells were then incubated in standard conditions for a further 72 h. A trypan blue assay was performed to determine cell viability. 

### 3.7. Mitochondrial Isolation and Standard Incubation Procedures

Rat liver was homogenized in isolation medium (250 mM sucrose, 5 mM HEPES, 0.5 mM EDTA, pH 7.4) and subjected to centrifugation (900× *g*) for 5 min. The supernatant was centrifuged at 12,000× *g* for 10 min to separate the crude mitochondrial pellets. The resulting pellets were suspended in isolation medium lacking EDTA [50]. Protein content was measured by the Biuret method with BSA as standard [51]. The isolation of the RLM was performed in accordance with the guiding principles in the care and use of animals and approved by the Italian Ministry of Health.

RLM (1 mg protein mL^−1^) were incubated in a water-jacketed cell at 20 °C. The standard medium contained 200 mM sucrose, 10 mM HEPES (pH 7.4), and 1 mM sodium phosphate. Variations and/or other additions are described in the specific experiments presented.

### 3.8. Determination of Mitochondrial Functions

The ∆Ψ was measured by assessing the distribution of the lipophilic cation tetraphenylphosphonium (TPP^+^) across the mitochondrial membrane with an ion-selective electrode specific for TPP^+^ [52] with a calomel reference electrode (Radiometer K401). In this method, the electrode potential was linear with respect to the logarithm of the TPP^+^ activity, with a slope of 58 mV, according to the Nernst equation and the law of mass conservation, and assuming an inner mitochondrial volume of 1.4 L/mg protein, calculated from the distributions of [^14^C] sucrose and ^3^H_2_O [53]. TPP^+^ was added at a concentration of 2 μmol L^−1^ to allow accurate measurements while avoiding toxic effects on the H^+^-ATPase and Ca^2+^ [52,53,54]. ∆Ψ measured with the TPP^+^ electrode was calibrated using the equation: ∆Ψ = (∆Ψ electrode−66.16)/0.92 [54]. The RCI was calculated by determining the difference in rates of oxygen uptake between respiratory state 4 (in the absence of 300 μmol L^−1^ ADP) and state 3 (in the presence of 300 μmol L^−1^ADP) using a Clark oxygen electrode (Yellow Springs Instrument Co. Inc., Yellow Springs, OH, USA) in a closed, thermostatically controlled vessel with a magnetic stirrer, coupled to a Perkin-Elmer 561 recorder [55]. The ADP/O ratio was calculated by dividing the concentration of added ADP by the corresponding oxygen uptake during respiratory state 3.

### 3.9. Determination of Mitochondrial Membrane Potential on Whole Cells

The mitochondrial ΔΨ was evaluated by using the BD™ MitoScreen Kit (BD Biosciences, San Jose, USA) containing the membrane-permeable lipophilic cationic fluorochrome 5,5′,6,6′-tetrachloro-1,1′,3,3′-tetraethylbenzimidazolcarbocyanine iodide (JC-1) [56]. HeLa cells (2.5 × 10^5^) were seeded into each cell culture plate. After 24 h of incubation, the test agent was added to the complete medium at the indicated concentration and cells were incubated for a further 28 h. After treatment, cells were centrifuged, resuspended in JC-1 working solution and incubated for 30 min at 37 °C in CO_2_ incubator. Following incubation, cells were washed twice, suspended in assay buffer and analyzed by a FACSCanto II flow cytometer. Results are presented as percentage of cells with depolarized mitochondrial membrane (JC-1 monomers) [56].

### 3.10. Evaluation of Apoptotic Cell Death

To detect phosphatidylserine translocation from the inner to the outer face of plasma membrane a FITC Annexin V Apoptosis Detection Kit I (BD Pharmingen, San Jose, USA) was used. HeLa cells (2.5 × 10^5^) were seeded into each cell culture plate. After incubation for 24 h the test acetogenin was added to the complete medium at the indicated concentration and the cells were incubated for a further 28 h. After treatment, the cells were centrifuged and suspended at 10^6^ cells/mL in binding buffer. Cell suspensions (100 μL) were added with Annexin V-FITC and propidium iodide (PI) as indicated by the supplier’s instructions and incubated for 15 min at room temperature in the dark. The populations of Annexin V-negative/PI-negative (viable), Annexin V-positive/PI-negative (early apoptosis), Annexin V-positive/PI-positive (late apoptosis) and Annexin V-negative/PI-positive (necrosis) cells were evaluated by FACSCanto II flow cytometer (Becton–Dickinson, Mountain View, CA, USA) [57].

## 4. Conclusions

Although a previous study reports the presence of an alkaloid [21], our results demonstrated that bis-THF acetogenins are also present in the roots of *A. purpurea*, which is in total agreement with previous studies of its leaves and seeds [16,19,20]. Five new acetogenins, named annopurpuricins A–E (**1**–**5**), were isolated from roots of *Annona purpurea*. Their structures and absolute configuration were determined by interpretation of the spectroscopic data of original compounds and some of their derivatives. Compounds **1**–**4** showed a significant cytotoxic activity on MSTO-211H, HeLa and HepG2 cancer cell lines with GI_50_ values in the nanomolar range. In particular, annopurpuricin A (**1**) a bis-THF acetogenin, was the main component of the DCM extract (0.018% yield) and showed a GI_50_ value notably low on HeLa cells (0.06 nM). The investigation on the mechanism of action of **1** highlighted the ability to inhibit NADH dehydrogenase (complex I) in RLM, similarly to rot. Cytofluorimetric experiments demonstrated that **1** is able to cause the depolarization of mitochondrial membrane in whole cells, and able to induce cell death through late apoptosis and necrosis.

## Figures and Tables

**Table 1:** ^1^H and ^13^C NMR data for compounds **1** and **2** recorded in CDCl_3_^a^.

	1		2	
Position	δ _H_ (*J* Hz)	δ _C_	δ _H_ (*J* Hz)	δ _C_
1	-	174.0 C	-	174.7 C
2	-	134.4 C	-	131.3 C
3a	2.24 br t	25.1 CH_2_	2.38 m	33.4 CH_2_
3b	2.24 br t	25.1 CH_2_	2.51 m	33.4 CH_2_
4	1.51 m	25.3 CH_2_	3.81 m	70.0 CH
5	1.29 m	29.4 CH_2_	1.62 m	29.6 CH_2_
6	3.82 m	71.4 CH	1.22 br s	29.6–29.8 CH_2_
7	1.33 m	32.0 CH_2_	1.22 br s	29.6–29.8 CH_2_
8	1.23 br s	29.5 CH_2_	1.22 br s	29.6–29.8 CH_2_
9	1.23 br s	29.5 CH_2_	1.22 br s	29.6–29.8 CH_2_
10	1.53 m	27.3 CH_2_	1.22 br s	29.6–29.8 CH_2_
11	3.88 m	82.5 CH	1.35 m	33.4 CH_2_
12	1.93 m	29.0 CH_2_	3.37 m	74.2 CH
13	1.77 m	24.6 CH_2_	3.79 m	83.2 CH
14	3.91 m	82.7 CH	1.93 m	29.1 CH_2_
15	3.78 m	82.1 CH	1.56 m	28.5 CH_2_
16	1.56 m	29.0 CH_2_	3.81 m	82.2 CH
17	1.94 m	28.5 CH_2_	3.91 m	82.8 CH
18	3.79 m	83.2 CH	1.81 m	24.6 CH_2_
19	3.38 m	74.1 CH	1.97 m	29.0 CH_2_
20	1.35 m	33.2 CH_2_	3.90 m	82.4 CH
21	1.23 br s	29.5 CH_2_	1.59 m	29.4 CH_2_
22	1.23 br s	29.5 CH_2_	1.45 m	37.5 CH_2_
23	1.40 m	37.3 CH_2_	3.84 m	71.5 CH
24	3.55 m	71.7 CH	1.32 m	32.0 CH_2_
25	1.39 m	37.4 CH_2_	1.22 br s	29.6–29.8 CH_2_
26	1.23 br s	25.1–29.2 CH_2_	1.22 br s	29.6–29.8 CH_2_
27	1.23 br s	25.1–29.2 CH_2_	1.22 br s	29.6–29.8 CH_2_
28	1.23 br s	25.1–29.2 CH_2_	1.22 br s	29.6–29.8 CH_2_
29	1.23 br s	25.1–29.2 CH_2_	1.22 br s	29.6–29.8 CH_2_
30	1.23 br s	25.1–29.2 CH_2_	1.22 br s	29.6–29.8 CH_2_
31	1.23 br s	25.1–29.2 CH_2_	1.22 br s	29.6–29.8 CH_2_
32	1.26 m	22.7 CH_2_	1.22 br s	29.6–29.8 CH_2_
33	1.27 m	32.0 CH_2_	1.28 m	32.5 CH_2_
34	0.86 t (6.76)	14.1 CH_3_	0.86 t (6.9)	14.2 CH_3_
35	6.97 br q (1.48)	149.0 CH	7.18 br d (1.3)	151.9 CH
36	4.98 dq (6.53, 5.12, 6.76)	77.4 CH	5.05 qq (5.48, 6.8, 6.84)	78.0 CH
37	1.38 d (6.8)	19.2 CH_3_	1.42 d (6.8)	19.2 CH_3_

^a^ δ from TMS (ppm). Assignments confirmed by COSY, HMBC and HSQC experiments (supplementary material) ^1^H NMR [400 MHz, CDCl_3_, *J* (Hz)] and ^13^C NMR (100 MHz) data for compounds **1** and **2**. br (broad signal).

**Table 2:** ^1^H and ^13^C NMR data for compounds **3**-**5**, recorded in CDCl_3_^a^.

	3		4		5	
Position	δ _H_ *(J* Hz)	δ _C_	δ _H_ (*J* Hz)	δ _C_	δ _H_ (*J* Hz)	δ _C_
1	-	174.0 C	-	174.6 C	-	174.0 C
2	-	134.5 C	-	131.3 C	-	134.5 C
3a	2.25 t	25.3 CH_2_	2.37 m	33.4 CH_2_	2.25 m	25.4 CH_2_
3b	2.25 t	25.3 CH_2_	2.50 m	33.4 CH_2_	2.25 m	25.4 CH_2_
4	1.51 m	27.5 CH_2_	3.82 m	70.0 CH	1.52 m	26.7 CH_2_
5	1.30 m	26.1 CH_2_	1.45 m	37.5 CH_2_	1.32 br s	27.4 CH_2_
6	1.24 br s	29.3-29.8 CH_2_	1.24 br s	29.6-29.8 CH_2_	1.32 br s	29.7-28.8 CH_2_
7	1.24 br s	29.3-29.8 CH_2_	1.24 br s	29.6-29.8 CH_2_	1.32 br s	29.7-28.8 CH_2_
8	1.24 br s	29.3-29.8 CH_2_	1.24 br s	29.6-29.8 CH_2_	1.32 br s	29.7-28.8 CH_2_
9	1.34 m	32.6 CH_2_	1.24 br s	29.6-29.8 CH_2_	1.32 br s	29.7-28.8 CH_2_
10	3.88 m	71.4 CH	1.45 m	25.76 CH_2_	1.32 br s	29.7-28.8 CH_2_
11	3.82 m	82.2 CH	1.37 m	33.3 CH_2_	1.51 m	27.9 CH_2_
12	1.94 m	29.1 CH_2_	3.38 m	74.2 CH	2.97 m	57.0 CH
13	1.78, 1.87 m	24.6 CH_2_	3.81 m	83.3 CH	2.95 m	56.9 CH
14	3.92 m	82.8 CH	1.95 m	29.0 CH_2_	2.95 m	56.8 CH
15	3.90 m	82.5 CH	1.59 m	28.5 CH_2_	2.95 m	57.4 CH
16	1.58, 1.95 m	29.0 CH_2_	3.80 m	82.3 CH	1.59 m	28.1 CH_2_
17	1.61 m	28.5 CH_2_	3.91 m	82.6 CH	2.21 m	24.4 CH_2_
18	3.80 m	83.3 CH	1.56, 1.96 m	29.0 CH_2_	5.37 m	128.2 CH
19	3.39 m	74.2 CH	1.88 m	24.6 CH_2_	5.40 m	131.3 CH
20	1.37 m	33.5 CH_2_	3.93 m	82.9 CH	2.02 m	27.5 CH_2_
21	1.24 br s	29.3–29.8 CH_2_	3.84 m	71.5 CH	1.32 m	29.4 CH_2_
22	1.24 br s	29.3–29.8 CH_2_	1.33 m	32.5 CH_2_	1.27 br s	29.7–28.8 CH_2_
23	1.24 br s	29.3–29.8 CH_2_	1.24 br s	29.6-29.8 CH_2_	1.27 br s	29.7–28.8 CH_2_
24	1.24 br s	29.3–29.8 CH_2_	1.24 br s	29.6-29.8 CH_2_	1.27 br s	29.7–28.8 CH_2_
25	1.24 br s	29.3–29.8 CH_2_	1.24 br s	29.6-29.8 CH_2_	1.27 br s	29.7–28.8 CH_2_
26	1.24 br s	29.3–29.8 CH_2_	1.24 br s	29.6-29.8 CH_2_	1.27 br s	29.7–28.8 CH_2_
27	1.24 br s	29.3–29.8 CH_2_	1.24 br s	29.6-29.8 CH_2_	1.27 br s	29.7–28.8 CH_2_
28	1.24 br s	29.3–29.8 CH_2_	1.24 br s	29.6-29.8 CH_2_	1.27 br s	29.7–28.8 CH_2_
29	1.24 br s	29.3–29.8 CH_2_	1.24 br s	29.6-29.8 CH_2_	1.27 br s	29.7–28.8 CH_2_
30	1.24 br s	29.3–29.8 CH_2_	1.24 br s	29.6-29.8 CH_2_	1.27 br s	29.7–28.8 CH_2_
31	1.24 br s	29.3–29.8 CH_2_	1.24 br s	29.6-29.8 CH_2_	1.27 br s	29.7–28.8 CH_2_
32	1.27 m	22.8 CH_2_	1.24 br s	32.0 CH_2_	1.24 br s	32.0 CH_2_
33	1.26 m	32.0 CH_2_	1.27 m	22.8 CH_2_	1.26 m	22.8 CH_2_
34	0.86 t (6.9)	14.2 CH_3_	0.85 t (6.9)	14.2 CH_3_	0.87 t (6.9)	14.2 CH_3_
35	6.98 q (1.48, 1.56, 1.52)	148.9 CH	7.18 br d (1.3)	151.8 CH	6.98 q (1.48, 1.56, 1.52)	148.9 CH
36	4.99 qq (5.08, 1.72, 5.08)	77.4 CH	5.04 qq (5.48, 6.8, 6.84)	78.0 CH	4.99 qq (5.08, 1.72, 5.08)	77.4 CH
37	1.39 d (6.8)	19.3 CH_3_	1.41 d (6.8)	19.2 CH_3_	1.40 d (6.8)	19.3 CH_3_

^a^ δ from TMS (ppm). Assignments confirmed by COSY, HMBC and HSQC experiments (supplementary material). ^1^H NMR [400 MHz, CDCl_3_, *J* (Hz)] and ^13^C NMR (100 MHz) spectroscopic data for compounds **3**, **4** and **5**. br (broad signal).

**Table 3:** ^1^H NMR data of the Mosher esters of **1**–**4**^a^.

MTPA Config.	Proton Chemical Shifts									Carbinol Configuration		
	**H (4)**	**H (5)**	**H (7)**	**H (10)**	**H (17)**	**H (18)**	**H (20)**	**H (23)**	**H (25)**	6*R*	19*R*	24*S*
1 S-MTPA	1.52	1.33	1.61	1.15	1.38	3.38	1.63	1.99	1.80			
1 R-MTPA	1.56	1.35	1.57	1.13	1.39	3.39	1.59	1.97	1.85			
∆δ_H_	−0.04	−0.02	+0.04	+0.02	−0.01	−0.01	+0.04	+0.02	−0.05			
	H (3) ^b^	H (5) ^b^	H (11)	H (13)	H (16)	H (17)	H (21)	H (24)	H (35)	4*R*	12*S*	23*S*
2 S-MTPA	2.58	1.61	1.41	3.91	3.82	3.39	1.92	1.62	6.73			
2 R-MTPA	2.64	1.59	1.61	4.05	3.76	3.37	1.58	1.63	6.98			
∆δ_H_	−0.06	+0.02	−0.20	−0.14	+0.06	+0.02	+0.34	−0.01	−0.25			
	H (4)	H (9)	H (11)	H (17)	H (18)	H (20)	H (33)	H (34)		10*R*	19*S*	
3 S-MTPA	1.52	1.64	3.34	1.38	3.34	1.64	1.41	0.92				
3 R-MTPA	1.54	1.58	3.41	1.94	3.41	1.58	1.33	0.89				
∆δ_H_	−0.02	+0.06	−0.07	−0.56	−0.07	+0.06	+0.08	+0.02				
	H (3)	H (5)	H (11)	H (13)	H (16)	H (17)	H (20)	H (22)	H (35)	4*R*	12*R*	21*S*
4 S-MTPA	2.59	1.65	1.63	3.40	3.72	3.67	3.85	1.36	6.72			
4 R-MTPA	2.64	1.61	1.48	3.99	3.82	3.64	3.93	1.59	6.97			
∆δ_H_	−0.05	+0.04	+0.23	−0.59	−0.1	+0.03	−0.08	−0.2	−0.25			

^a^ CDCl_3_, 400 MHz, ∆δ_H_ = δS-δR; ^b^ Independent experiments acquired in C_6_D_6_ were employed only to confirm the configuration at C-4 [∆δ_H_: H-3 (−0.01), H-5(+0.17)]. [30].

**Table 4:** Cell growth inhibition in the presence of compounds **1**–**5** and camptothecin taken as reference.

Compound	Cell Lines GI_50_^a^ (nM)		
	MSTO-211H	HeLa	HepG2
**1**	25.9 ± 4.5	0.06 ± 0.01	0.45 ± 0.16
**2**	6.9 ± 1.9	0.09 ± 0.02	0.66 ± 0.09
**3**	7.2 ± 1.6	0.41 ± 0.16	3.0 ± 1.3
**4**	7.3 ± 2.1	0.35 ± 0.06	2.5 ± 0.2
**5**	>50	>50	>50
Camptothecin	2.1 ± 0.1 *	5.4 ± 0.2 *	3.00 ± 0.20

* From ref. [40] ^a^ Means values ± of at least three independent experiments.

## Supplementary Materials

Supplementary Materials can be found at https://www.mdpi.com/1422-0067/20/8/1870/s1.

## Abbreviations

ADP	Adenosine diphosphate
ant A	Antimycin A
asc	Ascorbate
Bis-THF	Bis tetrahydrofuran
BSTFA	N,O-Bis(trimethylsilyl)trifluoroacetamide
B3LYP	Becke, three-parameter, Lee-Yang-Parr
OTMSi	O-trimethylsilyl ether
CD	Circular dichroism
C_6_D_6_	Benzene-*d_6_*
COSY	Correlated spectroscopy
DCM	Dichloromethane
ESI-TOF	Electrospray ionization time-of-flight mass spectrometry
FADH_2_	flavin adenine dinucleotide (reduced form)
FCCP	Carbonyl cyanide-4-(trifluoromethoxy)phenylhydrazone
FITC	Fluorescein isotiocyanate
FTIR	Fourier transform infrared spectroscopy
glu	Glutamate
HEPES	2-[4-(2-hydroxyethyl)piperazin-1-yl]ethanesulfonic acid
HMBC	Heteronuclear multiple bond correlation
HRMS	High-resolution mass spectra
HSQC	Heteronuclear single-quantum correlation
Hz	Hertz
JC-1	5,5′,6,6′-tetrachloro-1,1′,3,3′-tetraethylbenzimidazolocarbo-cyanine iodide
mal	Malate
mp	Melting point
MPT	Mitochondrial permeability transition
NADH	Nicotinamide adenine dinucleotide reduced form
^1^H NMR	Proton nuclear magnetic resonance
^13^C NMR	Carbon-13 nuclear magnetic resonance
S-MTPA	(S)-(−)-α-methoxy-α-(trifluoromethyl)phenylacetic acid
ppm	Parts per million
RCI	Respiration control index
rot	Rotenone
R-MTPA	(R)-(−)-α-methoxy-α-(trifluoromethyl)phenylacetic acid
RLM	Rat liver mitochondria
RR	Ruthenium red
succ	Succinate
THF	Tetrahydrofuran
TMPD	N,N,N′,N′-tetramethyl-p-phenylenediamine
TPP+	tetraphenylphosphonium cation
TMSi	Trimethylsilyl
UV	Ultraviolet–visible spectrophotometry
ΔΨ	Membrane potential

## References

[1] Bories C., Loiseau P., Cortes D., Myint S., Hocquemiller R., Gayral P., Cavé A., Laurens A. (1991). Antiparasitic activity of *Annona muricata* and *Annona cherimolia* seeds. Planta Med..

[2] Bourne R.K., Egbe P.C. (1979). A preliminary study of the sedative effects of *Annona muricata* (sour sop). West Indian Med. J..

[3] Cronquist A. (1981). An Integrated System of Classification of Flowering Plants.

[4] Hasrat J.A., Pieters L., De Backer J.-P., Vauquelin G., Vlietinck A.J. (1997). Screening of medicinal plants from Suriname for 5-HT1A ligands: Bioactive isoquinoline alkaloids from the fruit of *Annona muricata*. Phytomedicine.

[5] Martínez M. (1989). Las Plantas Medicinales de México.

[6] Emanuel R.V. (2016). Árboles de Minatitlán, Colima: Guía de usos tradicionales.

[7] Zafra-Polo M.C., González M.C., Estornell E., Sahpaz S., Cortes D. (1996). Acetogenins from annonaceae, inhibitors of mitochondrial complex I. Phytochemistry.

[8] Zafra-Polo M.C., Figadère B., Gallardo T., Tormo J., Cortes D. (1998). Natural acetogenins from annonaceae, synthesis and mechanisms of action. Phytochemistry.

[9] Melot A., Fall D., Gleye C., Champy P. (2009). Apolar annonaceous acetogenins from the fruit pulp of *Annona muricata*. Molecules.

[10] Mangal M., Khan M.I., Agarwal S.M. (2015). Acetogenins as potential anticancer agents. Anticancer Agents Med. Chem..

[11] Oberlies N.H., Chang C., McLaughlin J.L. (1997). Structure−Activity relationships of diverse annonaceous acetogenins against multidrug resistant human mammary adenocarcinoma (MCF-7/Adr) Cells. J. Med. Chem..

[12] Wu T.-Y., Yang I.-H., Tsai Y.-T., Wang J.-Y., Shiurba R., Hsieh T.-J., Chang F.-R., Chang W.-C. (2012). Isodesacetyluvaricin, an annonaceous acetogenin, specifically inhibits gene expression of cyclooxygenase-2. J. Nat. Prod..

[13] Kojima N., Fushimi T., Tatsukawa T., Tanaka T., Okamura M., Akatsuka A., Yamori T., Dan S., Iwasaki H., Yamashita M. (2014). Thiophene-3-carboxamide analogue of annonaceous acetogenins as antitumor drug lead. Eur. J. Med. Chem..

[14] Miyoshi H., Ohshima M., Shimada H., Akagi T., Iwamura H., McLaughlin J.L. (1998). Essential structural factors of annonaceous acetogenins as potent inhibitors of mitochondrial complex I. Biochim. Biophys. Acta BBA—Bioenerg..

[15] Nakanishi S., Abe M., Yamamoto S., Murai M., Miyoshi H. (2011). Bis-THF motif of acetogenin binds to the third matrix-side loop of ND1 subunit in mitochondrial NADH-ubiquinone oxidoreductase. Biochim. Biophys. Acta BBA—Bioenerg..

[16] Cepleanu F., Ohtani K., Hamburger M., Hostettmann K., Gupta M.P., Solis P. (1993). Novel acetogenins from the leaves of *Annona purpurea*. Helv. Chim. Acta.

[17] Chang F.R., Chen C.Y., Wu P.H., Kuo R.Y., Chang Y.C., Wu Y.C. (2000). New alkaloids from *Annona purpurea*. J. Nat. Prod..

[18] Chang F.-R., Wei J.-L., Teng C.-M., Wu Y.-C. (1998). Antiplatelet aggregation constituents from *Annona purpurea*. J. Nat. Prod..

[19] Chávez D., Mata R. (1999). Purpuracenin: A new cytotoxic adjacent bis-tetrahydrofuran annonaceous acetogenin from the seeds of *Annona purpurea*. Phytochemistry.

[20] Chávez D., Mata R. (1998). Purpurediolin and purpurenin, two new cytotoxic adjacent bis-tetrahydrofuran annonaceous acetogenins from the seeds of *Annona purpurea*. J. Nat. Prod..

[21] Rejón-Orantes J.D.C., González-Esquinca A.R., de la Mora M.P., Roldan Roldan G., Cortes D. (2011). Annomontine, an alkaloid isolated from *Annona purpurea*, has anxiolytic-like effects in the elevated plus-maze. Planta Med..

[22] Alali F.Q., Zhang Y., Rogers L., McLaughlin J.L. (1998). Mono-tetrahydrofuran acetogenins from *Goniothalamus giganteus*. Phytochemistry.

[23] Gawroński J., Wu Y.-C. (1999). A note on the determination of absolute configuration of acetogenins by circular dichroism. Pol. J. Chem..

[24] Cortes D., Figadere B., Cavé A. (1993). Bis-tetrahydrofuran acetogenins from annonaceae. Phytochemistry.

[25] Duret P., Waechter A.-I., Figadère B., Hocquemiller R., Cavé A. (1998). Determination of absolute configurations of carbinols of annonaceous acetogenins with 2-naphthylmethoxyacetic acid esters. J. Org. Chem..

[26] Rieser M.J., Hui Y.H., Rupprecht J.K., Kozlowski J.F., Wood K.V., McLaughlin J.L., Hanson P.R., Zhuang Z., Hoye T.R. (1992). Determination of absolute configuration of stereogenic carbinol centers in annonaceous acetogenins by proton and fluorine 19-NMR analysis of Mosher ester derivatives. J. Am. Chem. Soc..

[27] Gallardo T., Saez J., Granados H., Tormo J.R., Velez I.D., Brun N., Torres B., Cortes D. (1998). 10-Oximeguanacone, the first nitrogenated acetogenin derivative found to be a potent inhibitor of mitochondrial complex I. J. Nat. Prod..

[28] Gu Z., Zhou D., Lewis N.J., Wu J., Shi G., McLaughlin J.L. (1997). Isolation of new bioactive annonaceous acetogenins from *Rollinia mucosa* guided by liquid chromatography/mass spectrometry. Bioorg. Med. Chem..

[29] Liu X.-X., Alali F.Q., Hopp D.C., Rogers L.L., Pilarinou E., McLaughlin J.L. (1998). Glabracins A and B, two new acetogenins from *Annona glabra*. Bioorg. Med. Chem..

[30] Navrátilová H., de Gelder R., Kříž Z. (2002). Enantiodiscrimination in NMR Spectra and X-Ray Structures of Diastereomeric Salts of Trans-4-(4-Fluorophenyl)-3-Hydroxymethyl-1-Methylpiperidine with (S)-Mosher Acid. J. Chem. Soc. Perkin Trans..

[31] Zhao G.-X., Gu Z.-M., Zeng L., Chao J.-F., Kozlowski J.F., Wood K.V., McLaughlin J.L. (1995). The absolute configuration of trilobacin and trilobin, a novel highly potent acetogenin from the stem bark of *Asimina triloba* (Annonaceae). Tetrahedron.

[32] Raynaud S., Fourneau C., Hocquemiller R., Sévenet T., Hadi H.A., Cavé A. (1997). Acetogenins from the bark of *Uvaria pauci-ovulata*. Phytochemistry.

[33] Alali F.Q., Liu X.-X., McLaughlin J.L. (1999). Annonaceous acetogenins: Recent progress. J. Nat. Prod..

[34] Chih H.-W., Chiu H.-F., Tang K.-S., Chang F.-R., Wu Y.-C. (2001). Bullatacin, a potent antitumor annonaceous acetogenin, inhibits proliferation of human hepatocarcinoma cell line 2.2.15 by apoptosis induction. Life Sci..

[35] Chen Y.-Y., Chang F.-R., Yen H.-F., Wu Y.-C. (1996). Epomusenins A and B, two acetogenins from fruits of *Rollinia mucosa*. Phytochemistry.

[36] Gleye C., Laurens A., Hocquemiller R., Laprévote O., Serani L., Cavé A. (1997). Cohibins A and B, acetogenins from roots of *Annona muricata*. Phytochemistry.

[37] Meneses da Silva E.L., Roblot F., Mahuteau J., Cavé A. (1996). Coriadienin, the First Annonaceous Acetogenin with two double bonds isolated from *Annona coriaceae*. J. Nat. Prod..

[38] Tormo J.R., Zafra-Polo M.C., Serrano A., Estornell E., Cortes D. (2000). Epoxy-acetogenins and other polyketide epoxy derivatives as inhibitors of the mitochondrial respiratory chain complex I. Planta Med..

[39] Vázquez-Vuelvas O.F., Hernández-Madrigal J.V., Gaviño R., Tlenkopatchev M.A., Morales-Morales D., Germán-Acacio J.M., Gomez-Sandoval Z., Garcias-Morales C., Ariza-Castolo A., Pineda-Contreras A. (2011). X-ray, DFT, FTIR and NMR structural study of 2, 3-dihydro-2-(R-phenylacylidene)-1, 3, 3-trimethyl-1H-indole. J. Mol. Struc..

[40] Mazza A., Beccalli E.M., Contini A., Garcia-Argaez A.N., Dalla Via L., Gelmi M.L. (2016). A new scaffold of topoisomerase I inhibitors: Design, synthesis and biological evaluation. Eur. J. Med. Chem..

[41] Afonso S., Silva F.B., Silva A.F., Scarminio I.S., Bruns R.E. (2017). Infrared spectral evidence and DFT calculations of hydrogen-bonding and molecular structures of acetogenins. J. Mol. Struct..

[42] Zoratti M., Szabò I. (1995). The mitochondrial permeability transition. Biochim. Biophys. Acta.

[43] Susin S.A., Lorenzo H.K., Zamzami N., Marzo I., Snow B.E., Brothers G.M., Mangion J., Jacotot E., Costantini P., Loeffler M. (1999). Molecular characterization of mitochondrial apoptosis-inducing factor. Nature.

[44] Schummer C., Delhomme O., Appenzeller B.M.R., Wennig R., Millet M. (2009). Comparison of MTBSTFA and BSTFA in derivatization reactions of polar compounds prior to GC/MS analysis. Talanta.

[45] Frisch M.J., Trucks G.W., Schlegel H.B., Scuseria G.E., Robb M.A., Cheeseman J.R., Scalmani G., Barone V., Petersson G.A., Nakatsuji H. (2009). Gaussian 09, Revision A.02.

[46] Becke A.D. (1993). Density-functional thermochemistry. III. The role of exact exchange. J. Chem. Phys..

[47] Chai J.D., Head-Gordon M. (2008). Long-range corrected hybrid density functionals with damped atom–atom dispersion corrections. Phys. Chem. Chem. Phys..

[48] Scott A.P., Radom L. (1996). Harmonic vibrational frequencies: An evaluation of Hartree−Fock, Møller−Plesset, quadratic configuration interaction, density functional theory, and semiempirical scale factors. J. Phys. Chem..

[49] NIST N. (2010). Computational chemistry comparison and benchmark database. NIST Standard Reference Database Number 101.

[50] Schneider W.C., Hogeboom G.H. (1951). Cytochemical studies of mammalian tissues; the isolation of cell components by differential centrifugation: A review. Cancer Res..

[51] Gornall A.G., Bardawill C.J., David M.M. (1949). Determination of serum proteins by means of the biuret reaction. J. Biol. Chem..

[52] Kamo N., Muratsugu M., Hongoh R., Kobatake Y. (1979). Membrane potential of mitochondria measured with an electrode sensitive to tetraphenyl phosphonium and relationship between proton electrochemical potential and phosphorylation potential in steady state. J. Membr. Biol..

[53] Palmieri F., Klingenberg M., Methods E., Fleisher S., Packer L. (1979). Direct methods for measuring metabolite transport and distribution in mitochondria. Biomembranes Part G: Bioenergetics: Biogenesis of Mitochondria, Organization, and Transport.

[54] Gunter T.E., Jensen B.D. (1986). The efficiencies of the component steps of oxidative phosphorylation. I. A simple steady state theory. Arch. Biochem. Biophys..

[55] Estabrook R.W., Methods E., Estabrook R.W., Pullman M.E. (1967). Mitochondrial respiratory control and the polarographic measurement of ADP: O ratios. Oxidation and Phosphorylation.

[56] Cossarizza A., Baccaranicontri M., Kalashnikova G., Franceschi C. (1993). A new method for the cytofluorometric analysis of mitochondrial membrane potential using the J-aggregate forming lipophilic cation 5,5′,6,6′-Tetrachloro-1,1′,3,3′-tetraethylbenzimidazolcarbocyanine iodide (JC-1). Biochem. Biophys. Res. Commun..

[57] Van Engeland M., Nieland L.J., Ramaekers F.C., Schutte B., Reutelingsperger C.P. (1998). Annexin V-affinity assay: A review on an apoptosis detection system based on phosphatidylserine exposure. Cytometry.

