# Supplementary material for: Cytotoxic Acetogenins from the Roots of Annona purpurea
Source: Int J Mol Sci. 2019 Apr 16;20(8):1870. doi: 10.3390/ijms20081870 (PMC6515252; doi:10.3390/ijms20081870)
Supplement: Supplementary file 1 [file ijms-20-01870-s001.pdf]

Article

Cytotoxic Acetogenins from the Roots of *Annona purpurea*

Gustavo A. Hernández-Fuentes [1], Aída Nelly García-Argáez [2,3], Ana L. Peraza Campos [1], Iván Delgado-Enciso [4], Roberto Muñiz-Valencia [1], Francisco J. Martínez-Martínez [1], Antonio Toninello [5], Zeferino Gómez-Sandoval [1], Juan Pablo Mojica-Sánchez [1], Lisa Dalla Via [2],*, Hortensia Parra-Delgado [1],*.

1. *Facultad de Ciencias Químicas, Universidad de Colima, Carretera Colima-Coquimatlán km 9, 28400, Coquimatlán, Colima, México.*
2. *Dipartimento di Scienze del Farmaco, Università degli Studi di Padova, Via F. Marzolo 5, 35131 Padova, Italy*
3. *Fondazione per la Biologia e la Medicina della Rigenerazione T.E.S.-Tissue Engineering and Signalling Onlus, Via F. Marzolo, 13, 35131 Padova, Italy.*
4. *Facultad de Medicina, Universidad de Colima, Av. Universidad 333, Las Víboras, 28040 Colima, México.*
5. *Dipartimento di Scienze Biomediche, Università degli Studi di Padova, Via G. Colombo 3, 35121, Padova, Italy.*

* Correspondence:

Prof. Lisa Dalla Via. Phone: +39 (049) 8275712. Fax: +39 (049) 8275366. E-mail: lisa.dallavia@unipd.it.
Prof. Hortensia Parra-Delgado. Phone and fax: +52 (312) 3161163. E-mail: hparra@ucol.mx

Supplementary files

Table of Contents

S1 ^{1}H NMR (400 MHz, $CDCl_3$) spectrum of compound 1
S2 ^{13}C NMR (100 MHz, $CDCl_3$) spectrum of compound 1
S3 COSY experiment ($CDCl_3$) of 1
S4 HSQC experiment ($CDCl_3$) of 1
S5 HMBC experiment ($CDCl_3$) of 1
S6 Mass spectrum (IE) of 1
S7 HRMS (ESI-TOF) of compound 1
S8 Mass spectrum of the TMSi derivative of 1
S9 Correlations in COSY and ^{1}H NMR spectrum (C_6D_6) of 1
S10 ^{1}H NMR (400 MHz, $CDCl_3$) spectrum of compound 1a
S11 COSY experiment ($CDCl_3$), Mosher ester *R* of 1
S12 COSY experiment ($CDCl_3$), Mosher ester *S* of 1
S13 IR theoretical calculations of 1

S14 ^{1}H NMR (400 MHz, $CDCl_3$) spectrum of compound 2
S15 ^{13}C NMR (100 MHz, $CDCl_3$) spectrum of compound 2
S16 COSY experiment ($CDCl_3$) of 2
S17 HSQC experiment ($CDCl_3$) of 2
S18 HMBC experiment ($CDCl_3$) of 2
S19 Mass spectrum (IE) of 2
S20 HRMS (ESI-TOF) of compound 2
S21 Mass spectrum of the TMSi derivative of 2
S22 Correlations in COSY and ^{1}H NMR spectrum (C_6D_6) of 2
S23 ^{1}H NMR (400 MHz, $CDCl_3$) spectrum of compound 2a
S24 COSY experiment ($CDCl_3$), Mosher ester *R* of 2

S25 COSY experiment ($CDCl_3$), Mosher ester *S* of 2
S26 IR theoretical calculations of 2

S27 ^{1}H NMR (400 MHz, $CDCl_3$) spectrum of compound 3
S28 ^{13}C NMR (100 MHz, $CDCl_3$) spectrum of compound 3
S29 COSY experiment ($CDCl_3$) of 3
S30 HSQC experiment ($CDCl_3$) of 3
S31 HMBC experiment ($CDCl_3$) of 3
S32 Mass spectrum (IE) of 3
S33 HRMS (ESI-TOF) of compound 3
S34 Mass spectrum of the TMSi derivative of 3
S35 Correlations in COSY and ^{1}H NMR spectrum (C_6D_6) of 3
S36 ^{1}H NMR (400 MHz, $CDCl_3$) spectrum of compound 3a
S37 COSY experiment ($CDCl_3$), Mosher ester *R* of 3
S38 COSY experiment ($CDCl_3$), Mosher ester *S* of 3
S39 IR theoretical calculations of 3

S40 ^{1}H NMR (400 MHz, $CDCl_3$) spectrum of compound 4
S41 ^{13}C NMR (100 MHz, $CDCl_3$) spectrum of compound 4
S42 COSY experiment ($CDCl_3$) of 4
S43 HSQC experiment ($CDCl_3$) of 4
S44 HMBC experiment ($CDCl_3$) of 4
S45 Mass spectrum (IE) of 4
S46 Mass spectrum of the TMSi derivative of 4
S47 HRMS (ESI-TOF) of compound 4
S48 Correlations in COSY and ^{1}H NMR spectrum (C_6D_6) of 4
S49 ^{1}H NMR (400 MHz, $CDCl_3$) spectrum of compound 4a
S50 COSY experiment ($CDCl_3$), Mosher ester *R* of 4
S51 COSY experiment ($CDCl_3$), Mosher ester *S* of 4
S52 IR theoretical calculations of 4

S53 ^{1}H NMR (400 MHz, $CDCl_3$) spectrum of compound 5
S54 ^{13}C NMR (100 MHz, $CDCl_3$) spectrum of compound 5
S55 COSY experiment ($CDCl_3$) of 5
S56 HSQC experiment ($CDCl_3$) of 5
S57 HMBC experiment ($CDCl_3$) of 5
S58 Mass spectrum (IE) of 5
S59 Mass spectrum (ESI^+) of 5
S60 HRMS (ESI-TOF) of compound 5
S61 Correlations in COSY and ^{1}H NMR spectrum (C_6D_6) of 5
S62 IR theoretical calculations of 5

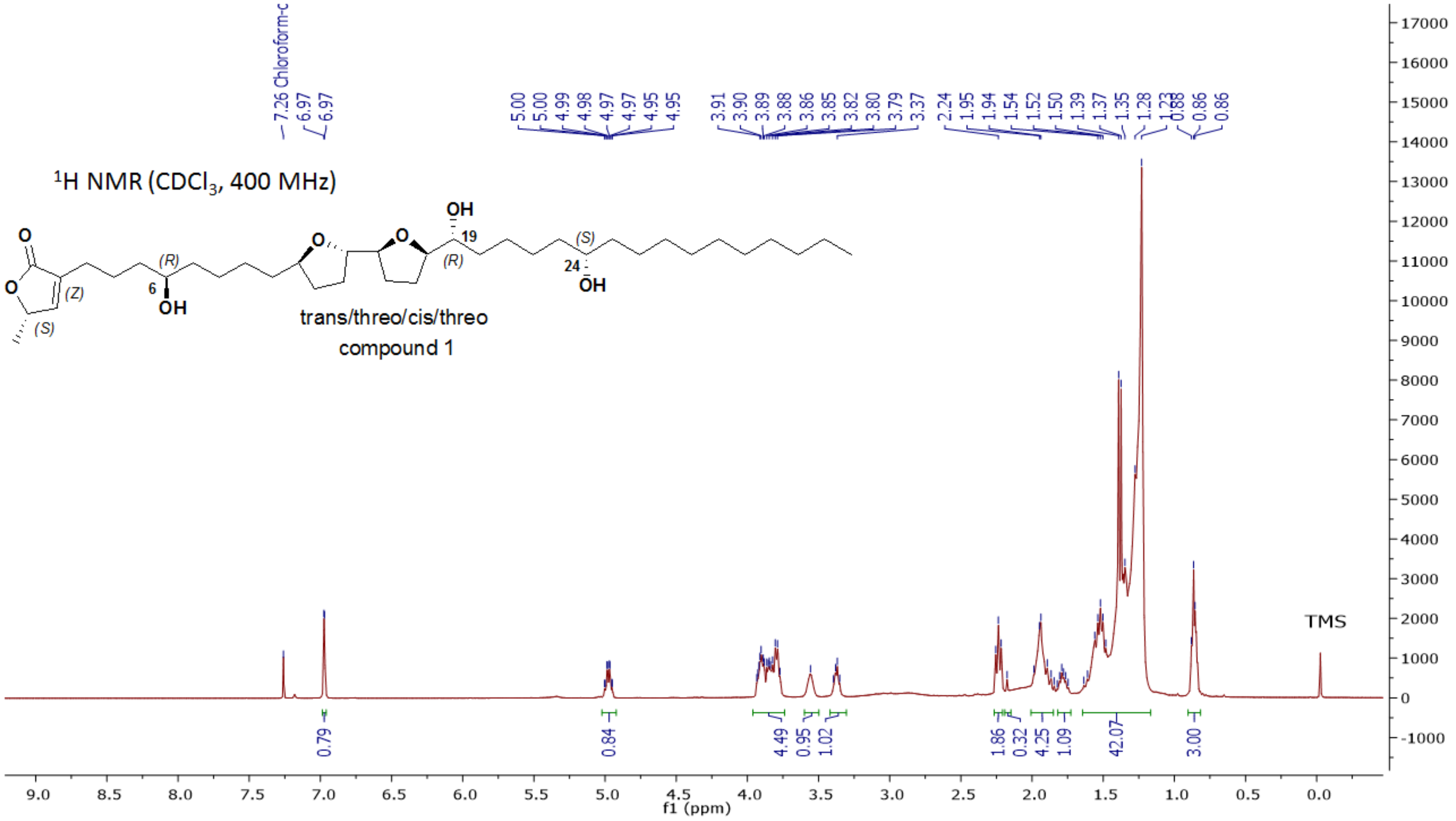

S1 ^{1}H NMR (400 MHz, $CDCl_3$) spectrum of compound 1

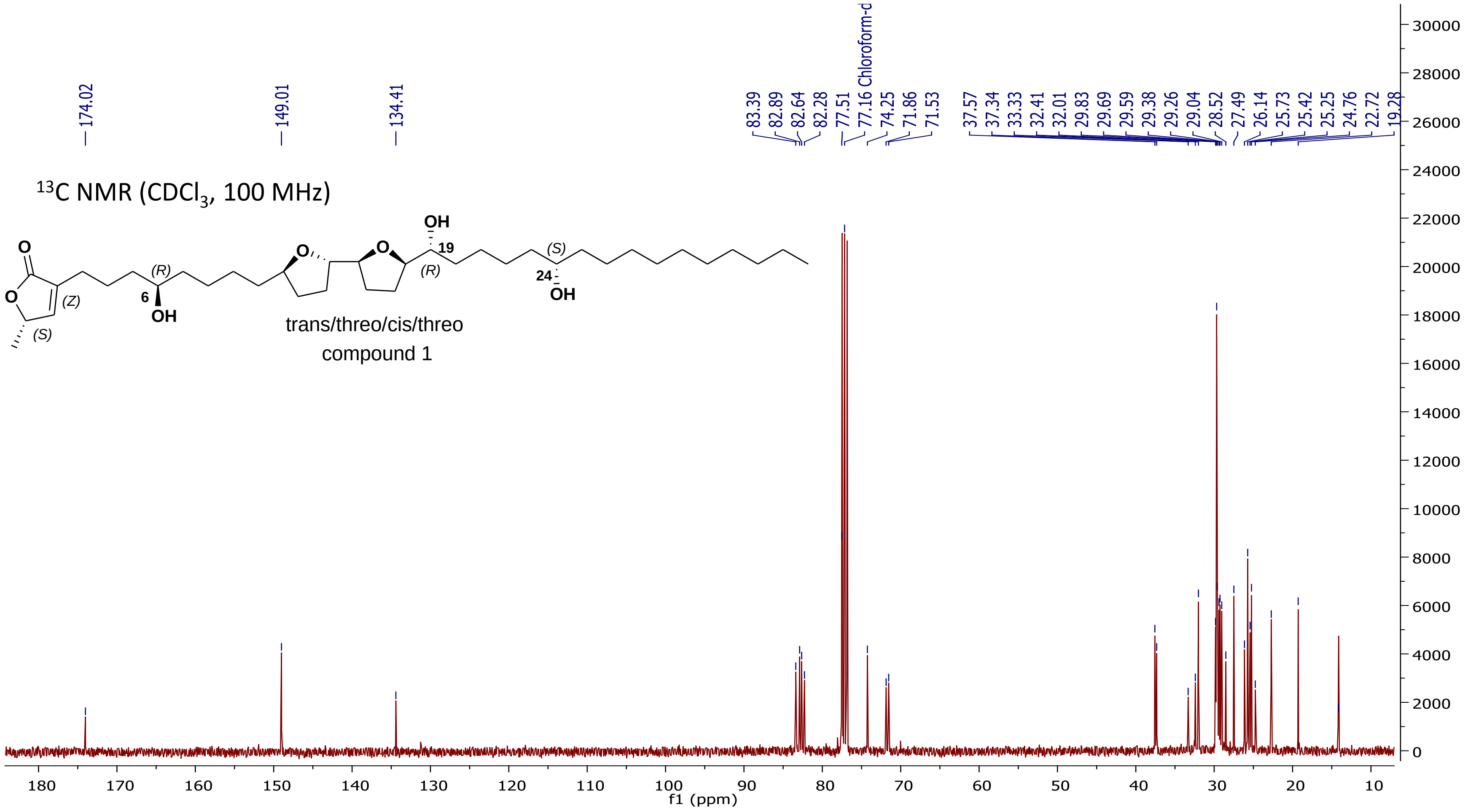

S2. ^{13}C NMR (100 MHz, $CDCl_3$) spectrum of compound 1

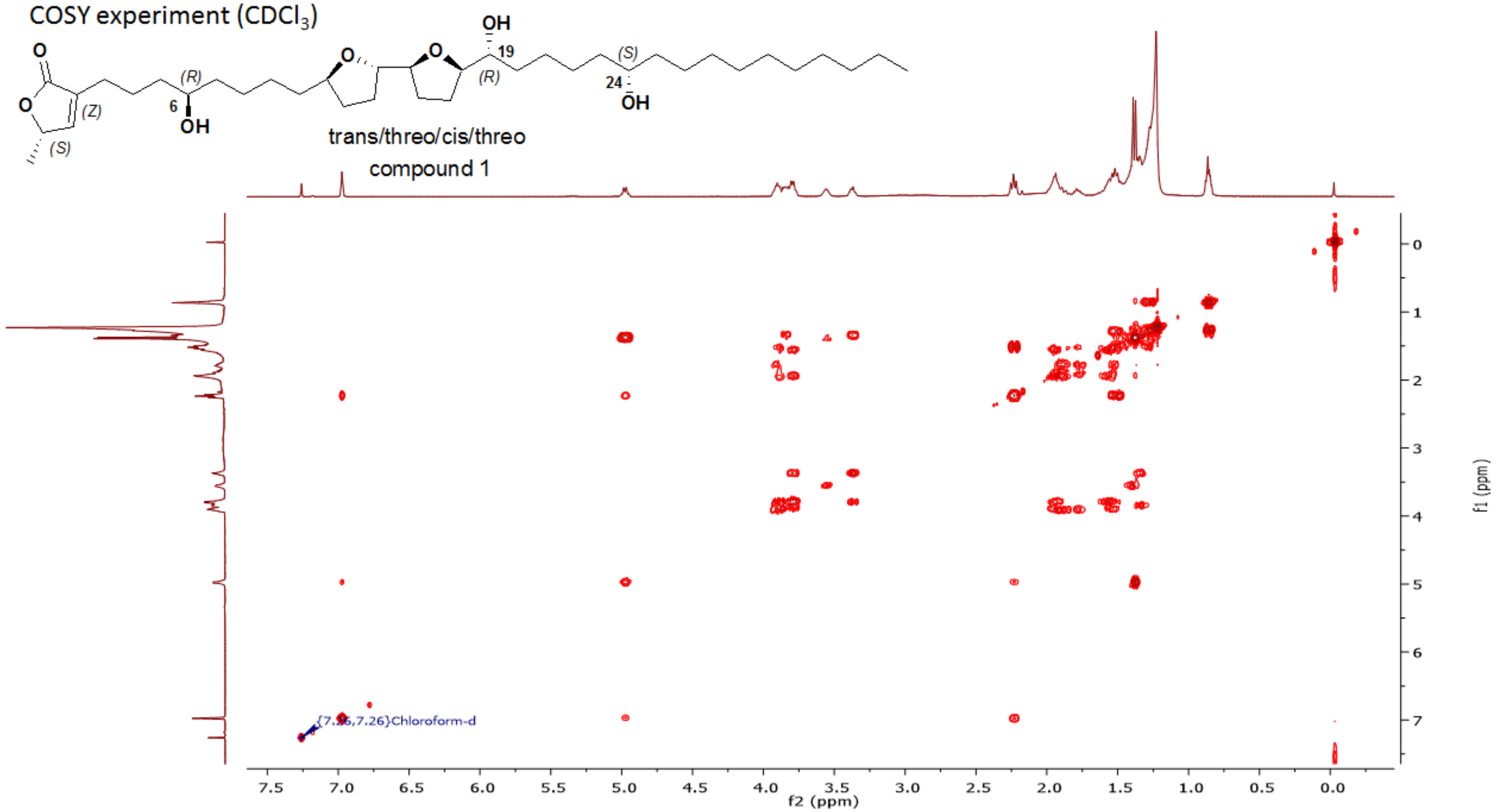

S3 COSY experiment (CDCl$_3$) of 1

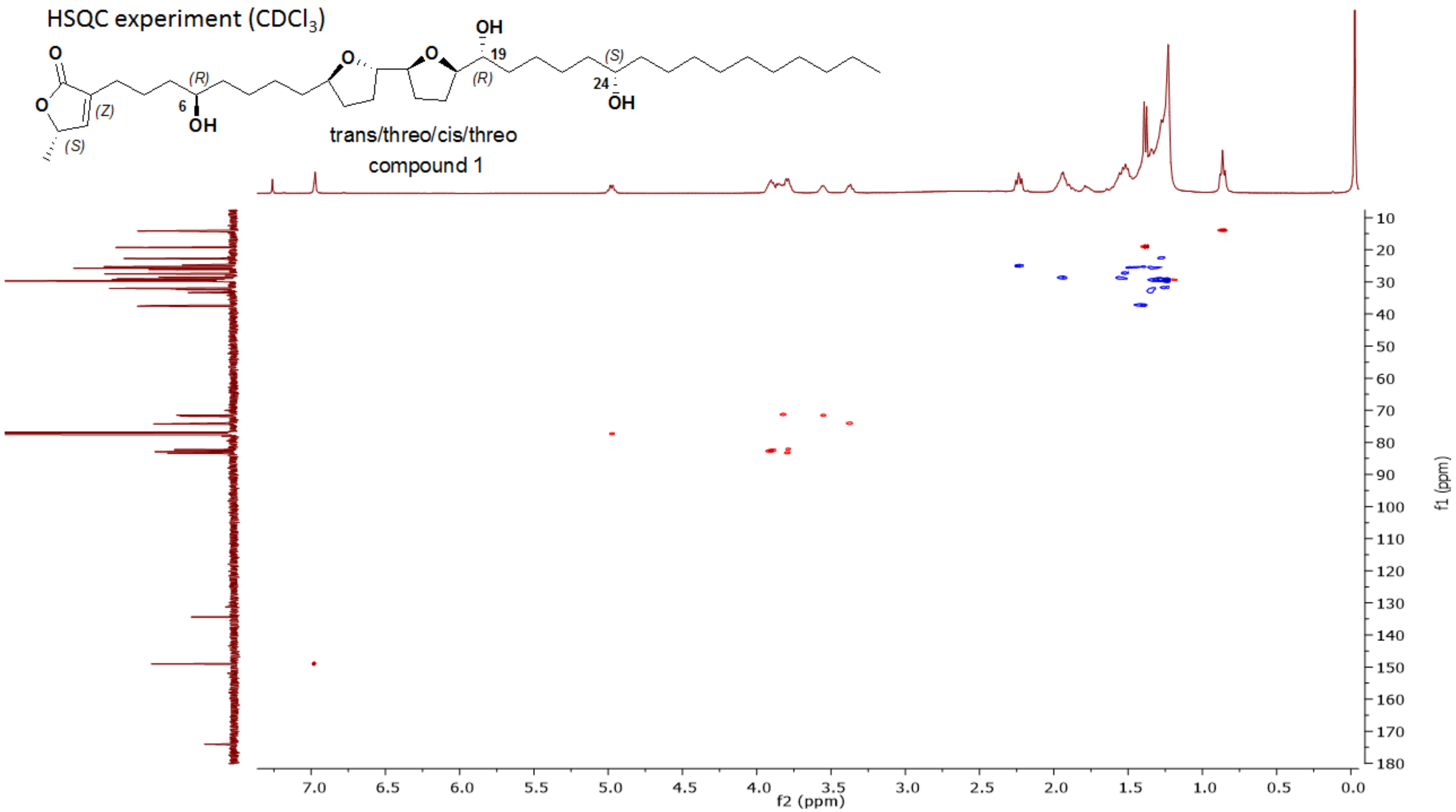

S4 HSQC experiment ($CDCl_3$) of 1

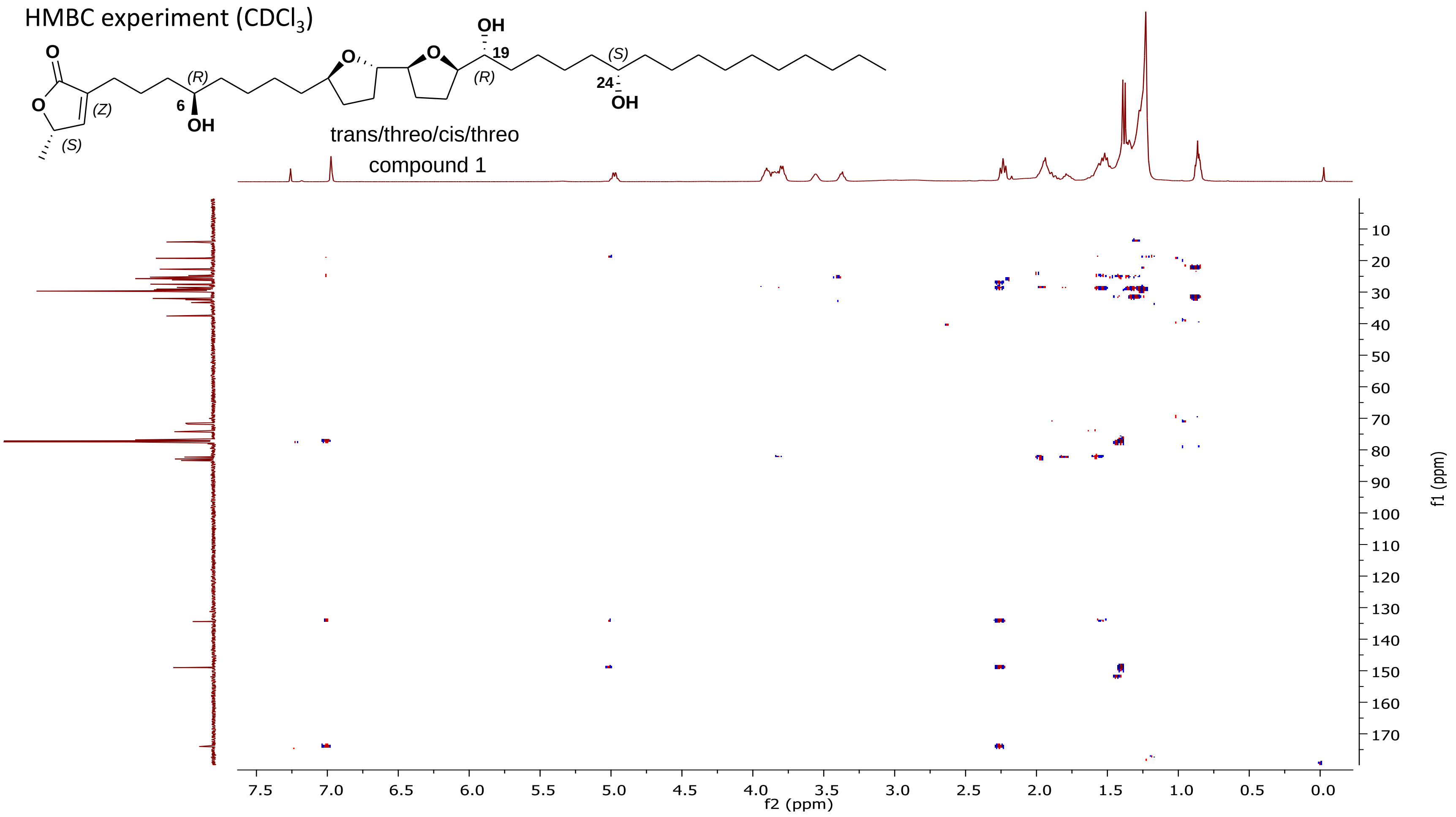

S5 HMBC experiment ($CDCl_3$) of 1

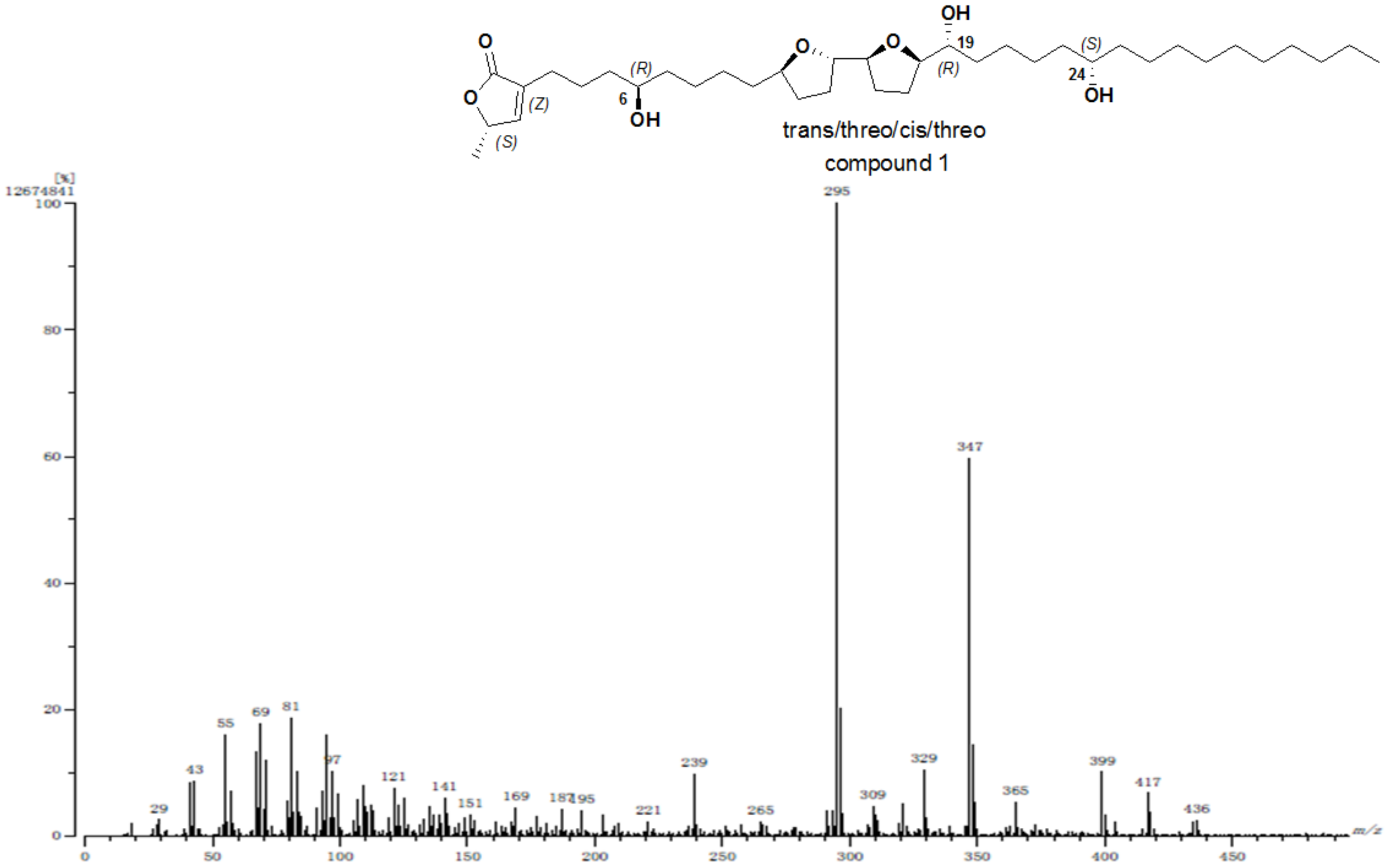

S6 Mass spectrum (IE) of 1

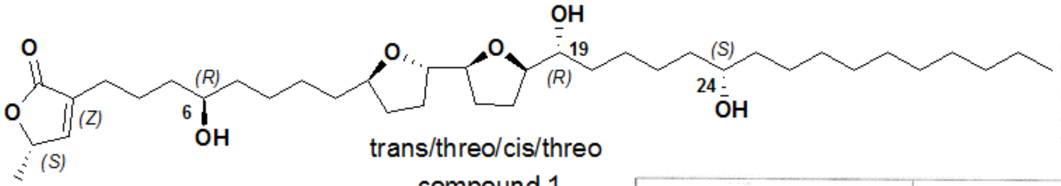

trans/threo/cis/threo
compound 1

Elemental composition calculator

```
Target m/z:          +645.4695      amu
Tolerance:             +3.0000      ppm
Result type:         Elemental
Max num of results:        1000
Min DBE:        -0.5000      Max DBE:          +100.0000
Electron state:      Even
Num of charges:      1
Add water:           N/A
Add proton:          N/A
File Name:           J-210618-Ar-1-9-01.wiff
```

	Elements	Min Number	Max Number
1	C	0	50
2	H	0	70
3	N	0	2
4	O	0	10
5	Na	0	2

	Formula	Calculated m/z (amu)	mDa Error	PPM Error	DBE
1	C37 H66 O7 Na	645.470076	-0.584240	-0.905138	4.5

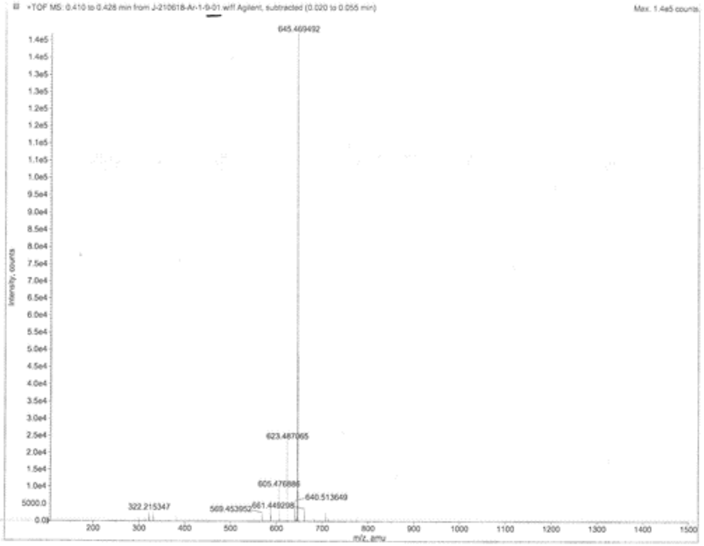

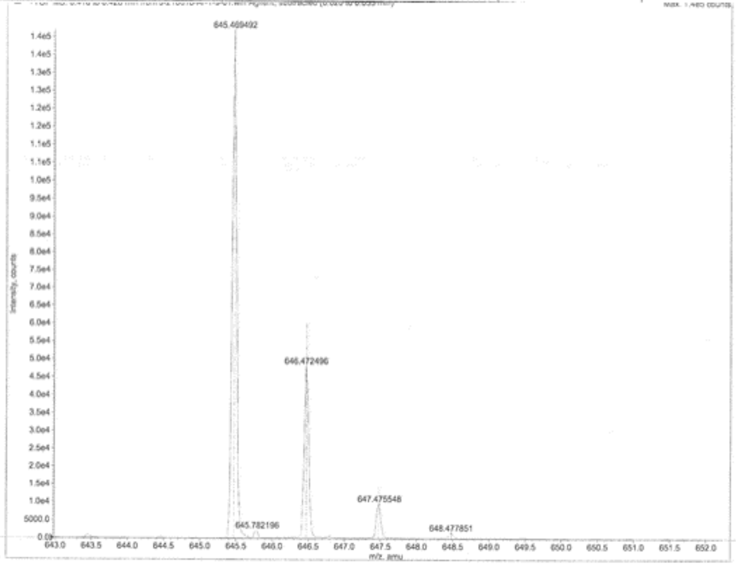

S7 HRMS (ESI-TOF) of compound 1

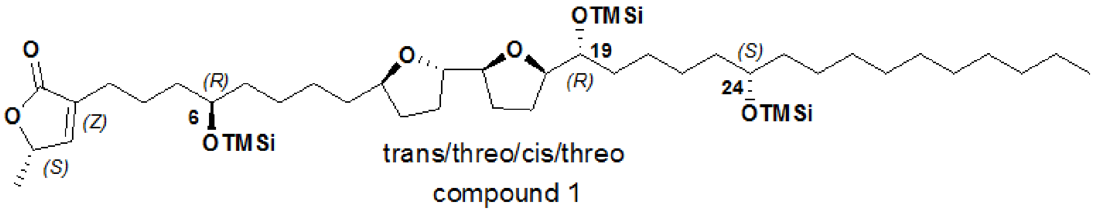

trans/threo/cis/threo
compound 1

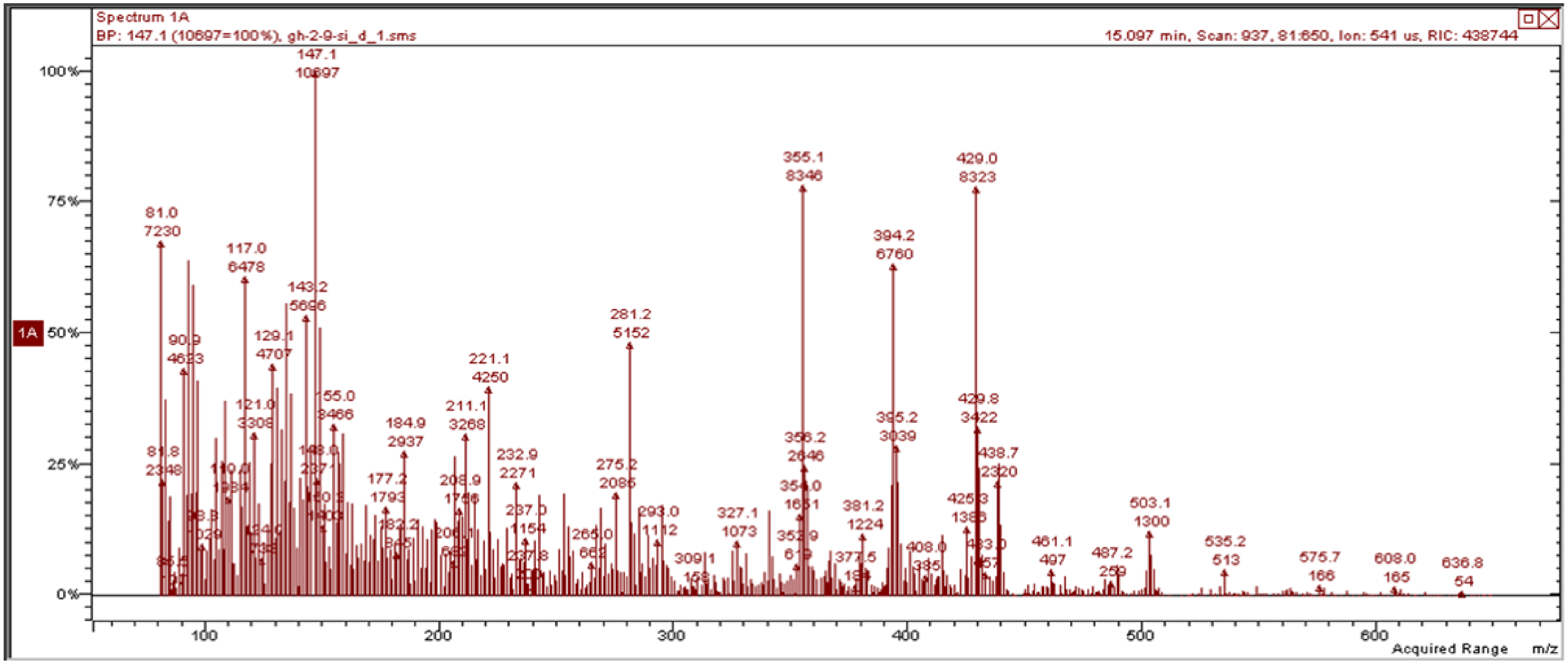

S8 Mass spectrum of the TMSi derivative of 1

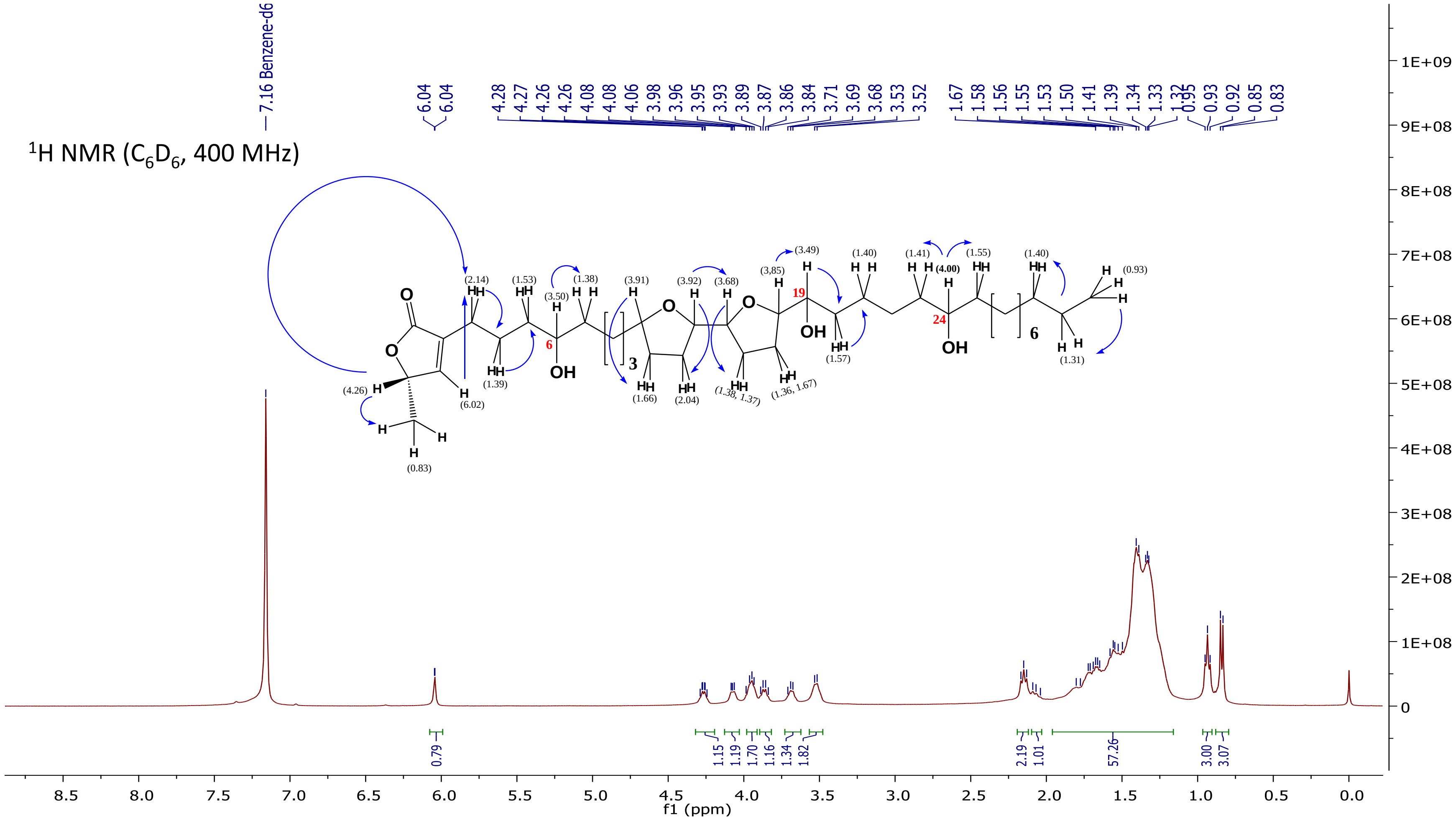

S9 Correlations in COSY and ^{1}H NMR spectrum (C_6D_6) of 1

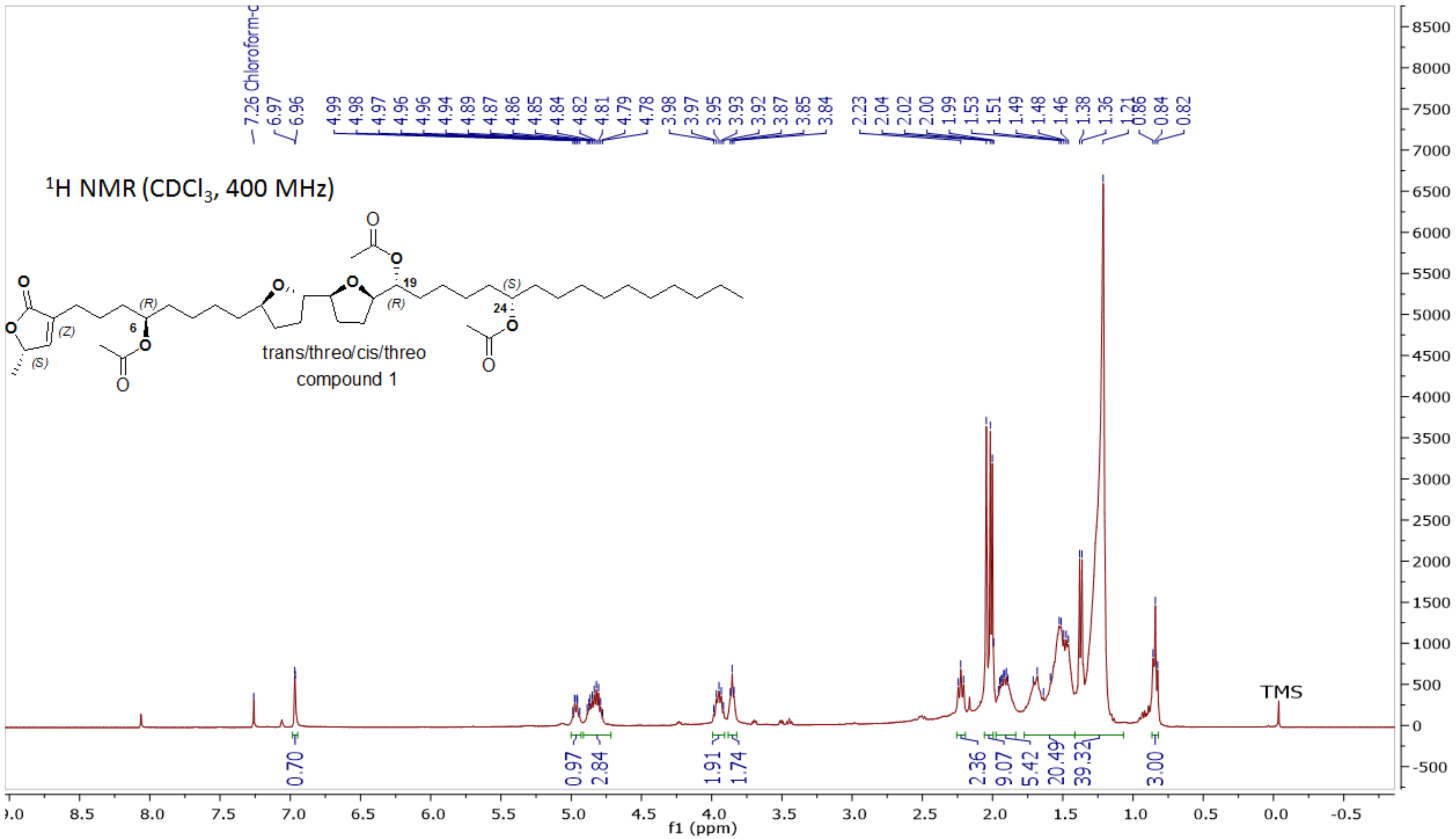

S10 ^{1}H NMR (400 MHz, $CDCl_3$) spectrum of compound 1a

COSY experiment, Mosher ester *R* ($CDCl_3$)

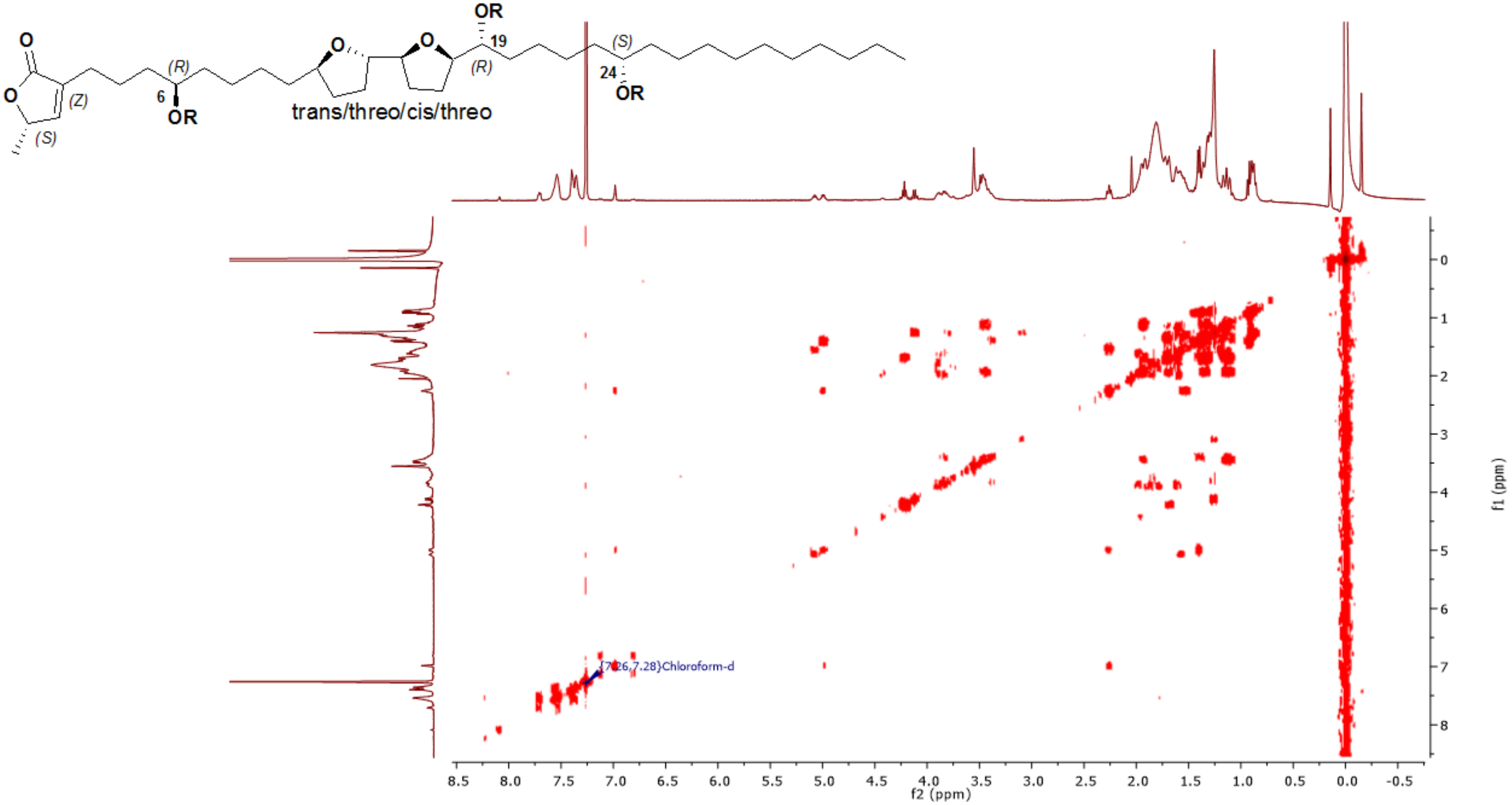

S11 COSY experiment ($CDCl_3$), Mosher ester of 1, R= (*R*)-MTPA

COSY experiment, Mosher ester *S* ($CDCl_3$)

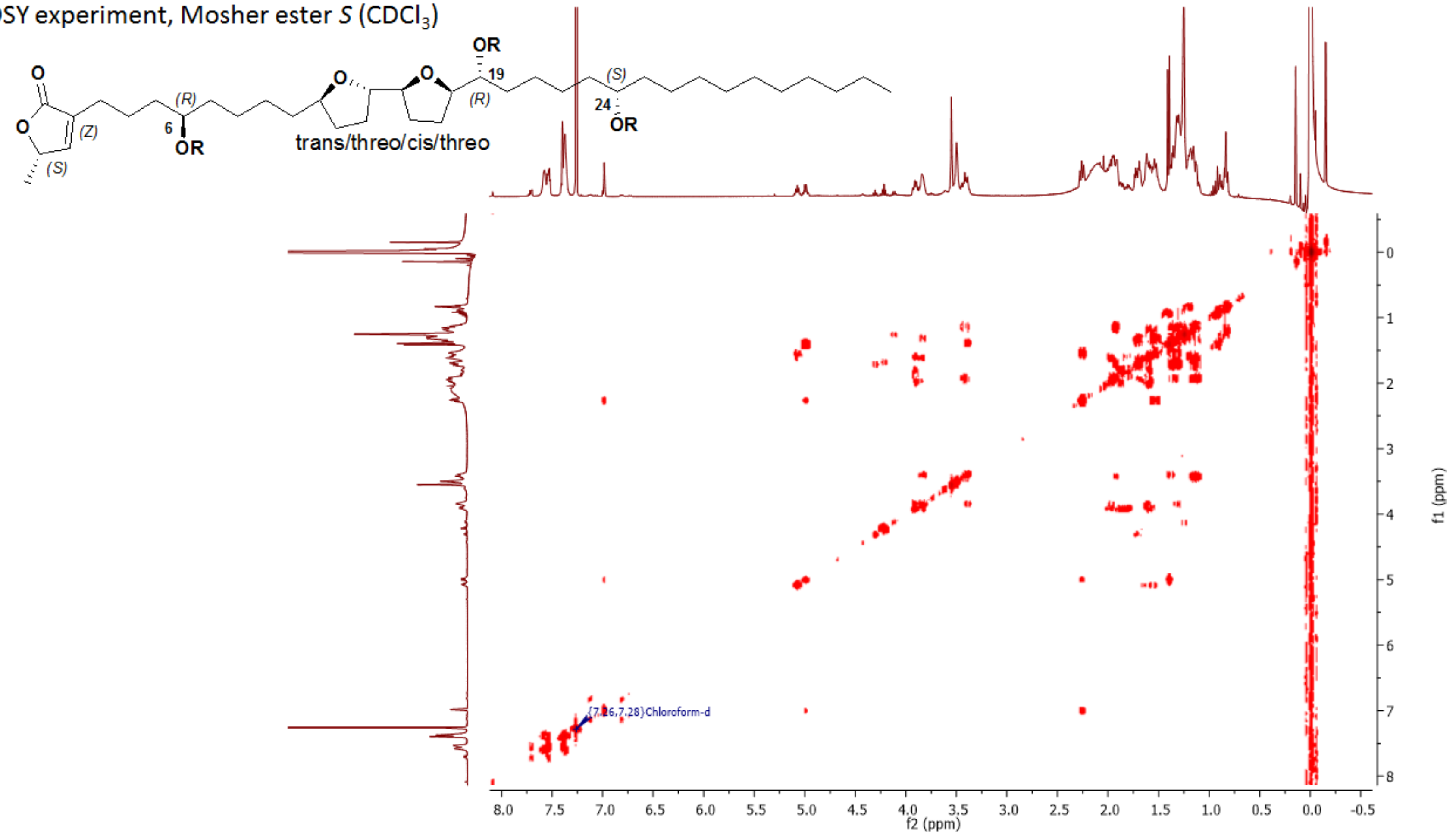

S12 COSY experiment ($CDCl_3$), Mosher ester of 1, R=(*S*)-MTPA

Annopurpuricin A		
Experimental	6-311G(d,p)/B3LYP[a]	6-311G(d,p)/ωB97XD[b]
3373.49	3651.98	3717.85
2916.36	2941.67	2917.35
2848.85	2863.30	2846.24
1742.68	1767.26	1805.48
1651.06	1641.57	1678.02
1471.68	1458.90	1469.24
1420.56	1423.38	1422.21
1371.38	1374.44	1373.00
1324.12	1324.52	1324.57
1203.57	1207.92	1203.50
1119.67	1120.29	1122.52
1080.13	1086.63	1082.00
1052.16	1050.23	1053.94
966.33	965.71	964.90
945.11	945.90	943.18
868.92	869.15	870.93
828.42	822.49	826.97
716.55	715.31	716.28
637.47	632.16	638.27
600.82	614.55	601.34

[a] Scaling factor of 0.9614
[b] Scaling factor of 0.957

S13 IR theoretical calculations of 1

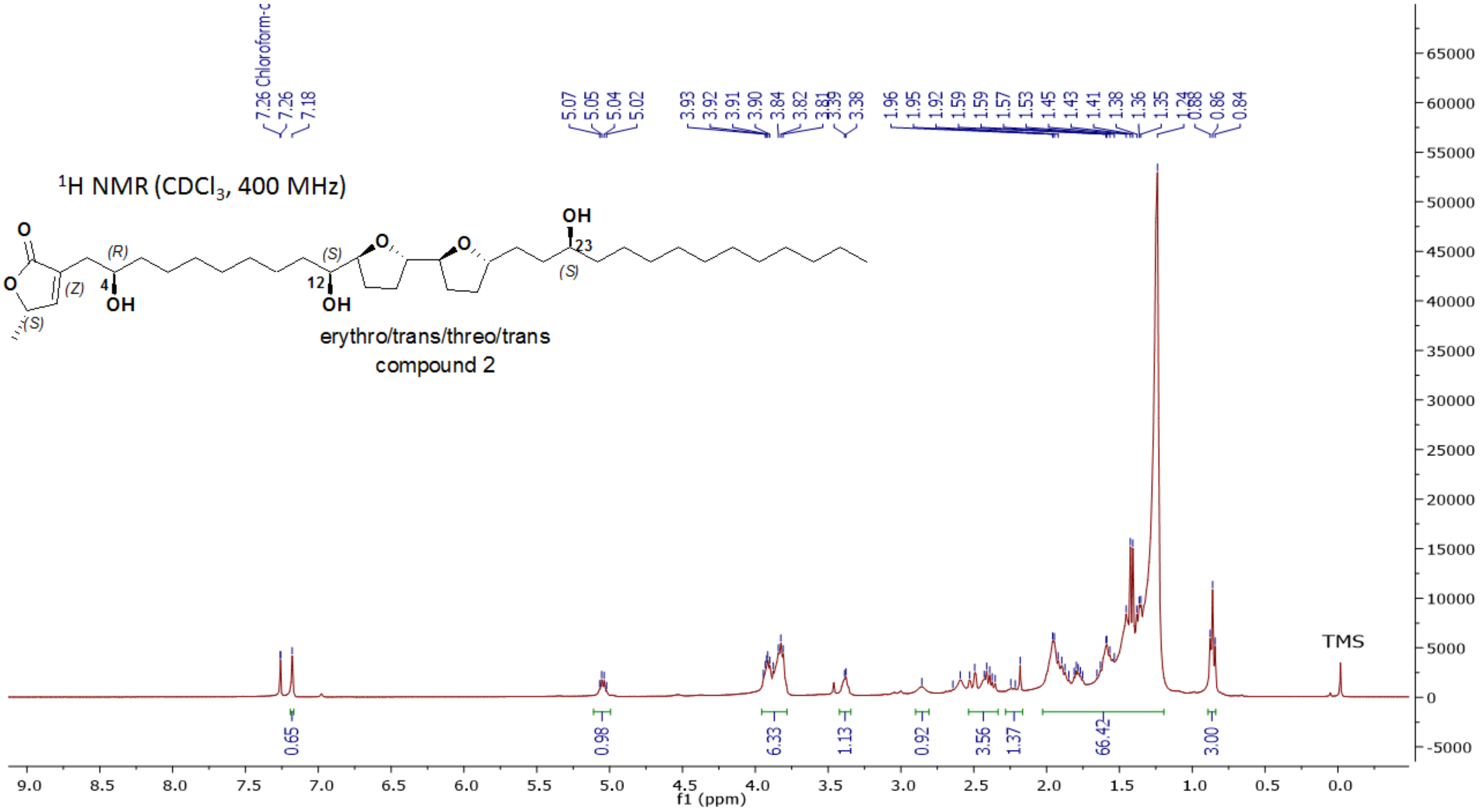

S14 ^{1}H NMR (400 MHz, $CDCl_3$) spectrum of compound 2

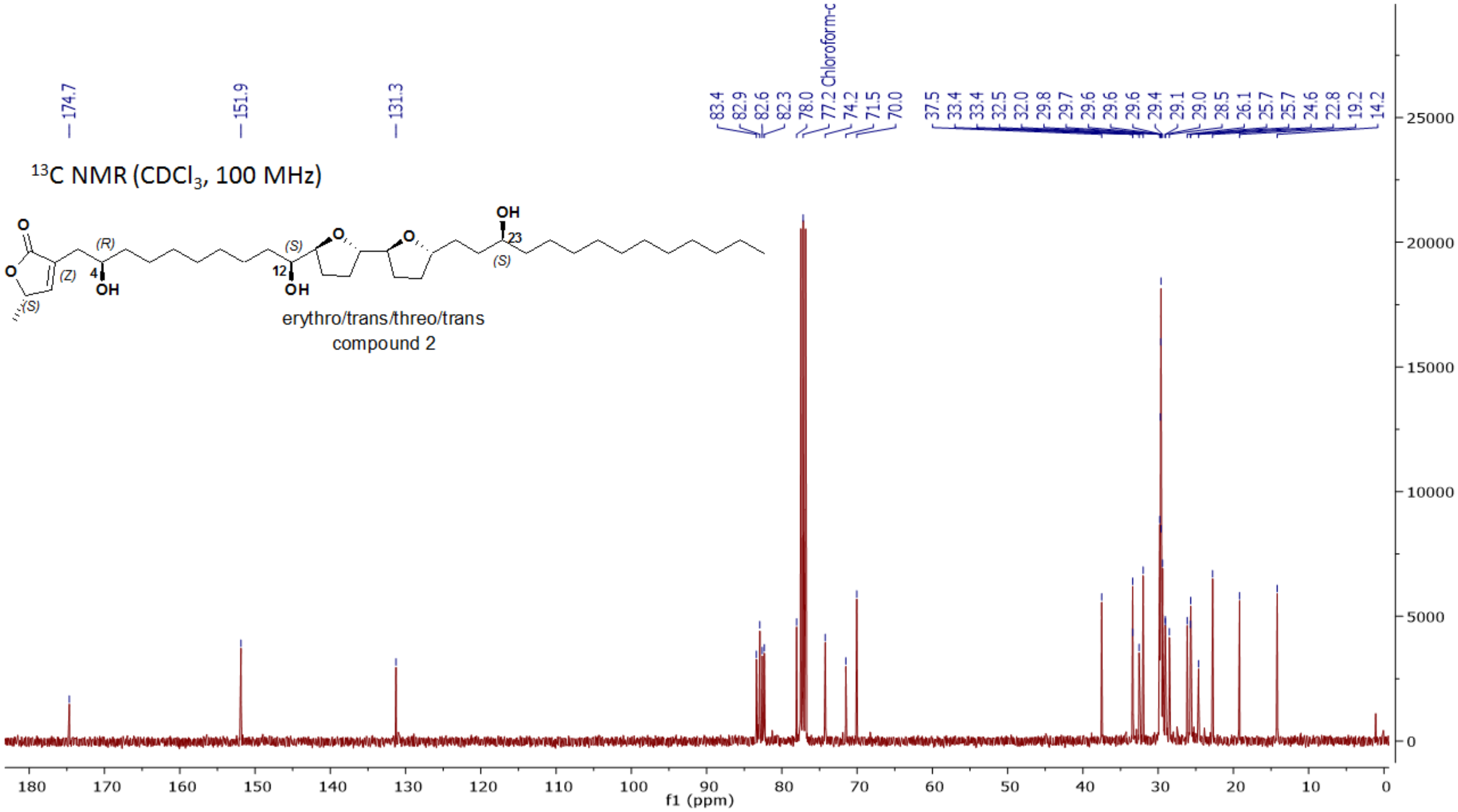

S15 ^{13}C NMR (100 MHz, $CDCl_3$) spectrum of compound 2

COSY experiment ($CDCl_3$)

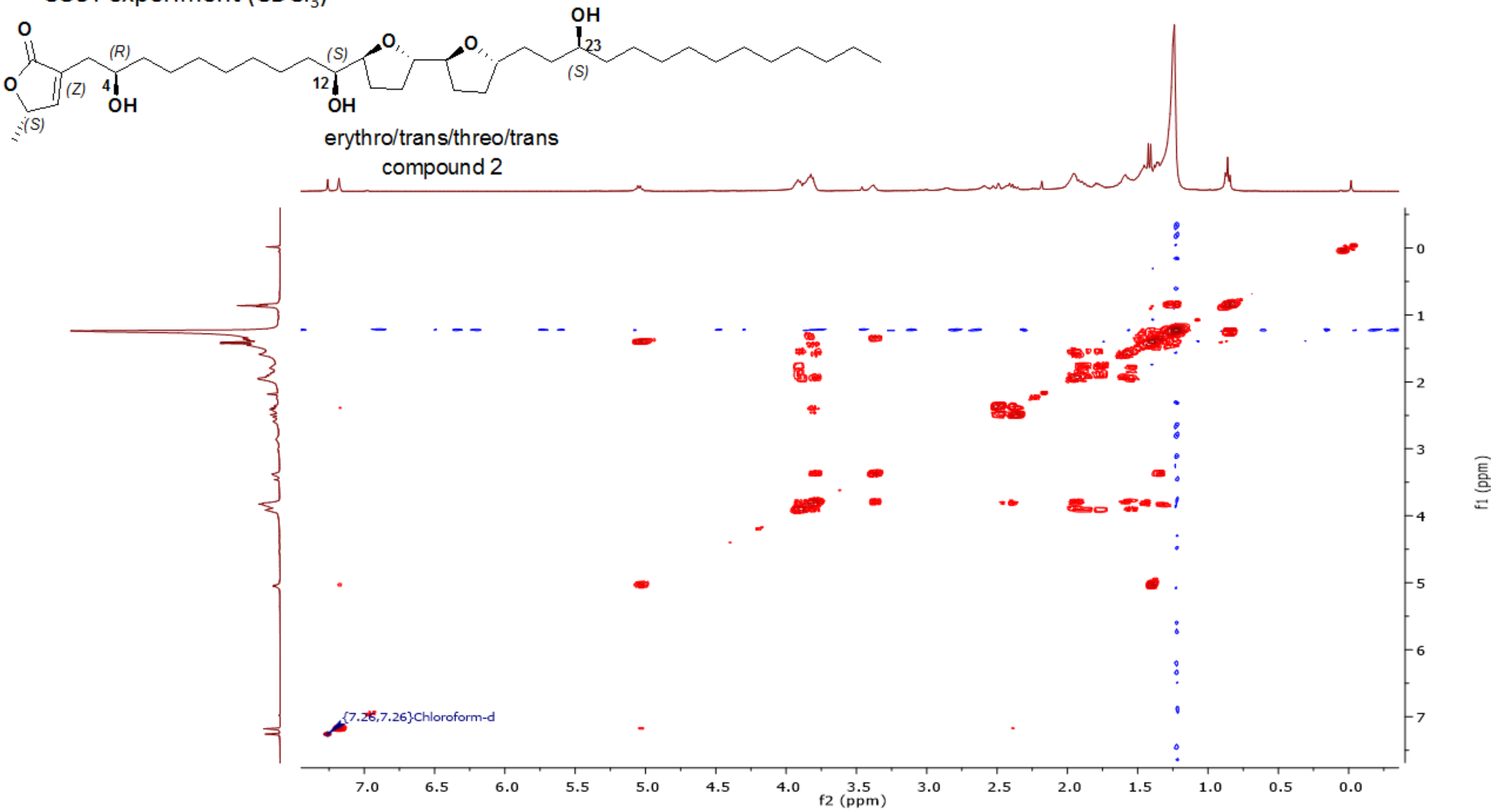

S16 COSY experiment ($CDCl_3$) of 2

HSQC experiment ($CDCl_3$)

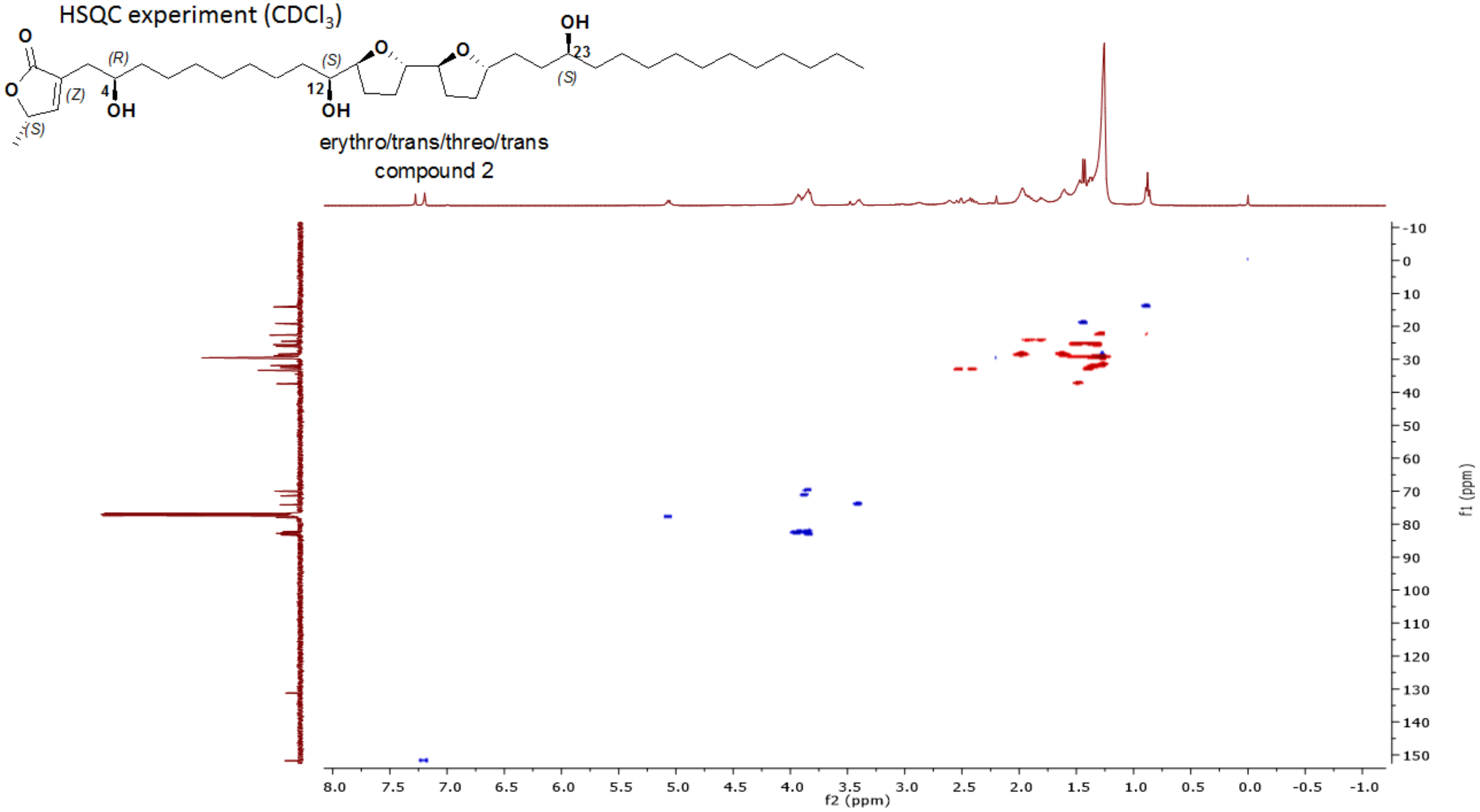

S17 HSQC experiment ($CDCl_3$) of 2

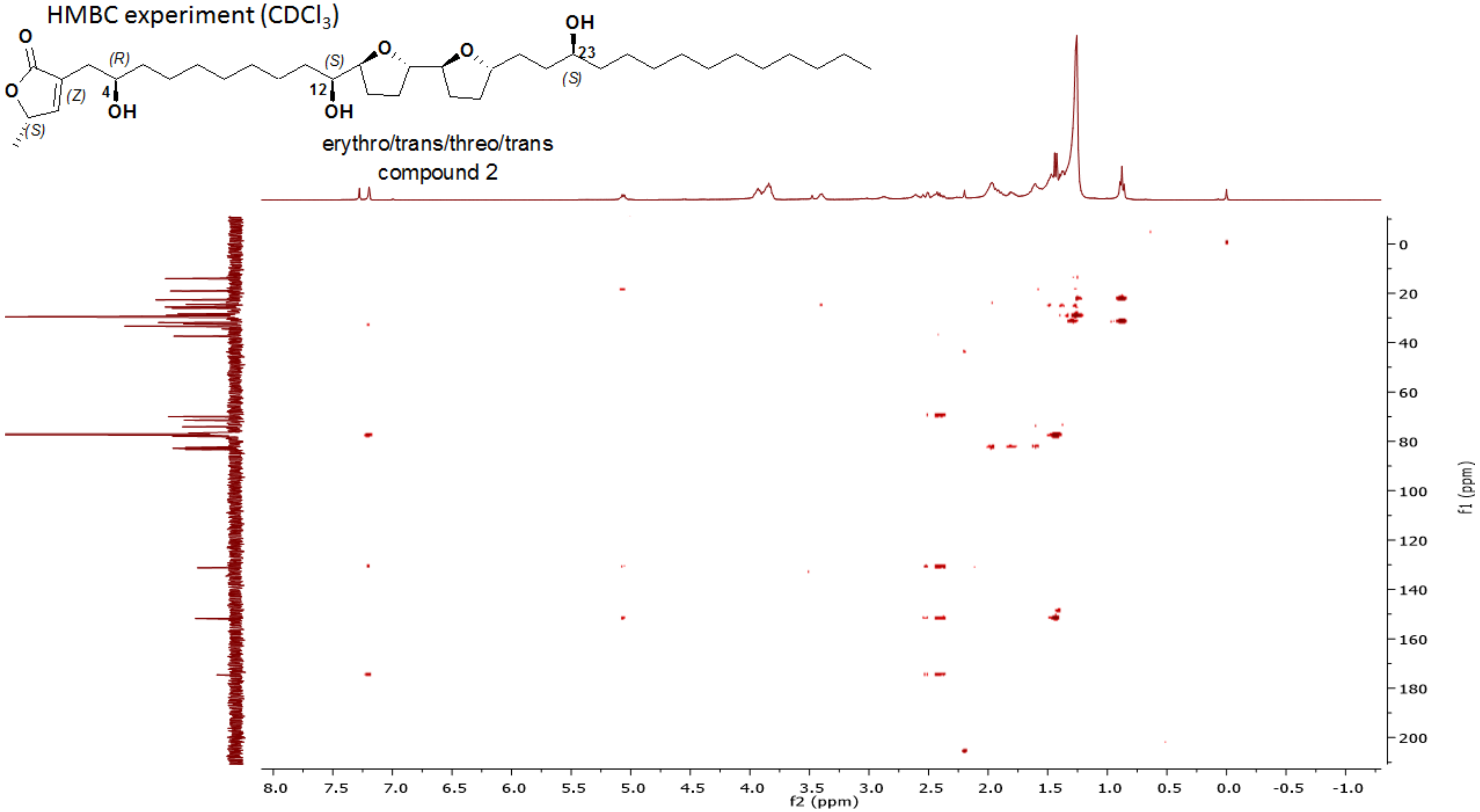

S18 HMBC experiment ($CDCl_3$) of 2

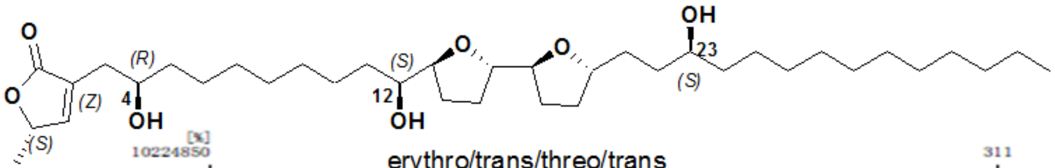

erythro/trans/threo/trans
compound 2

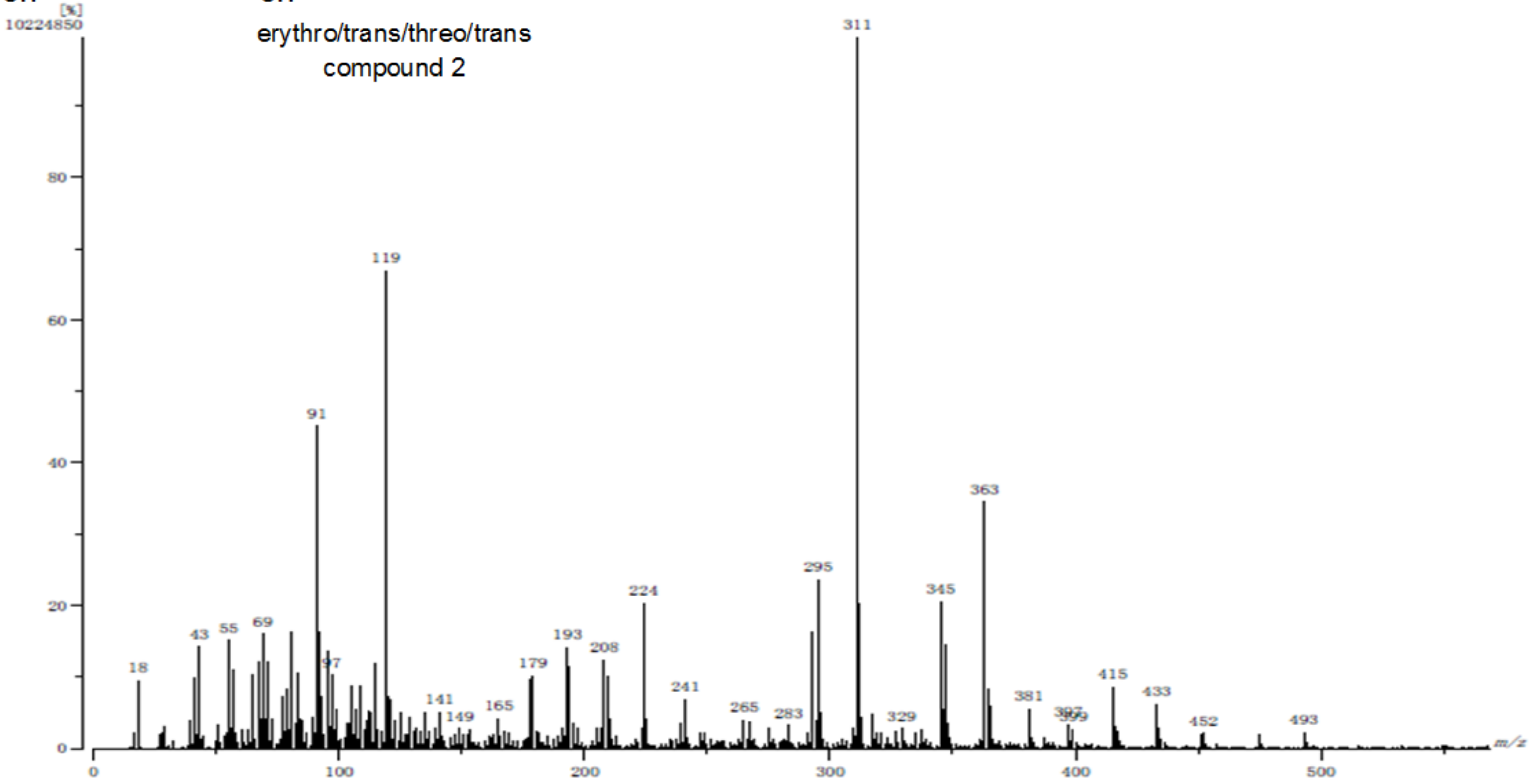

S19 Mass spectrum (IE) of 2

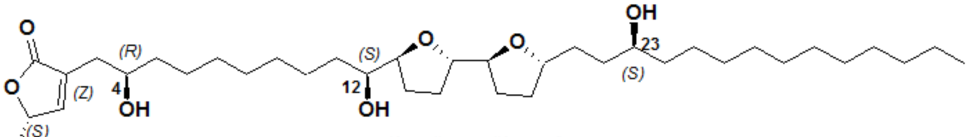

erythro/trans/threo/trans
compound 2

Elemental composition calculator

```
Target m/z:          +645.4700      amu
Tolerance:             +3.0000      ppm
Result type:         Elemental
Max num of results:        1000
Min DBE:     -0.5000      Max DBE:          +100.0000
Electron state:   Even
Num of charges:   1
Add water:        N/A
Add proton:       N/A
File Name:        J-210618-Ar-1-10-01.wiff
```

	Elements	Min Number	Max Number
1	C	0	50
2	H	0	70
3	N	0	2
4	O	0	10
5	Na	0	2

	Formula	Calculated m/z (amu)	mDa Error	PPM Error	DBE
1	C37 H66 O7 Na	645.470076	-0.120240	-0.186282	4.5

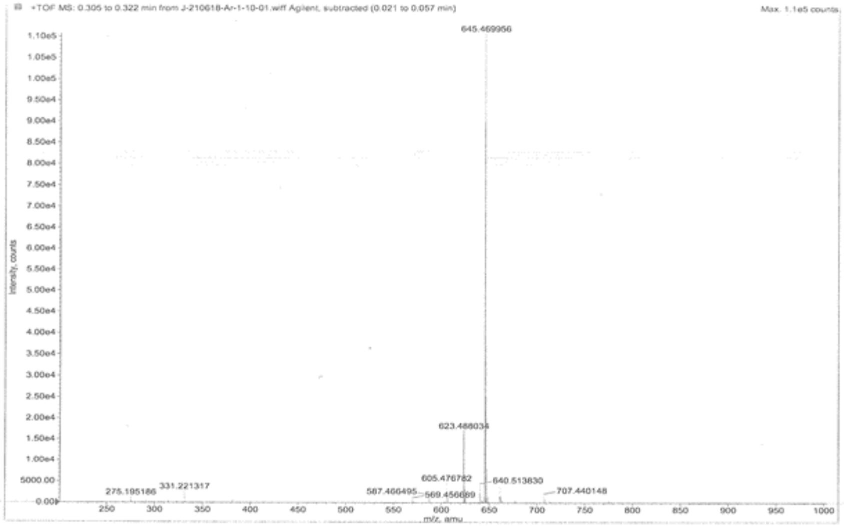

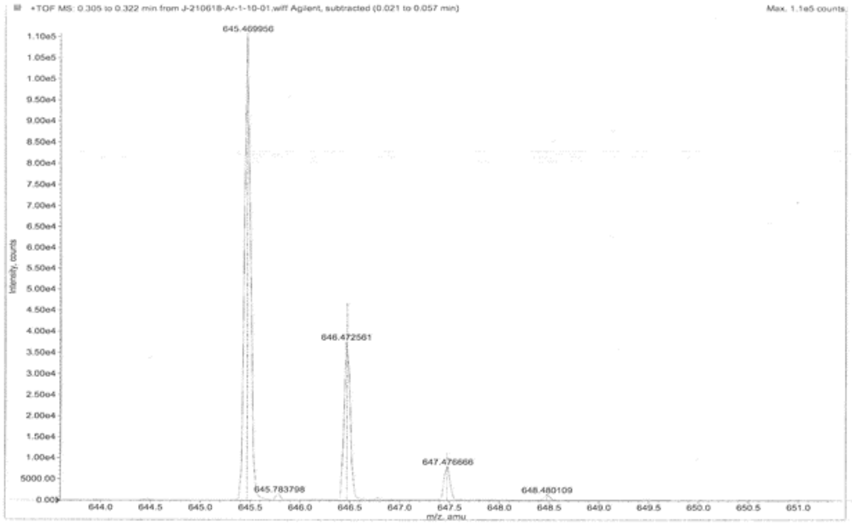

S20 HRMS (ESI-TOF) of compound 2

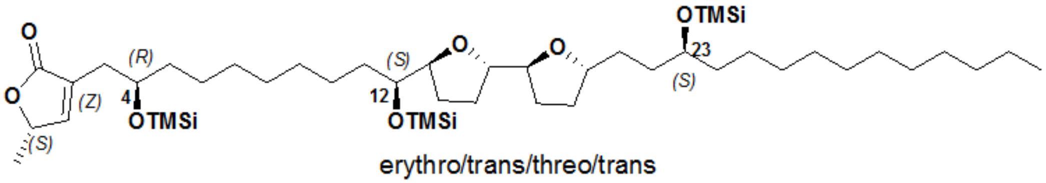

erythro/trans/threo/trans
compound 2

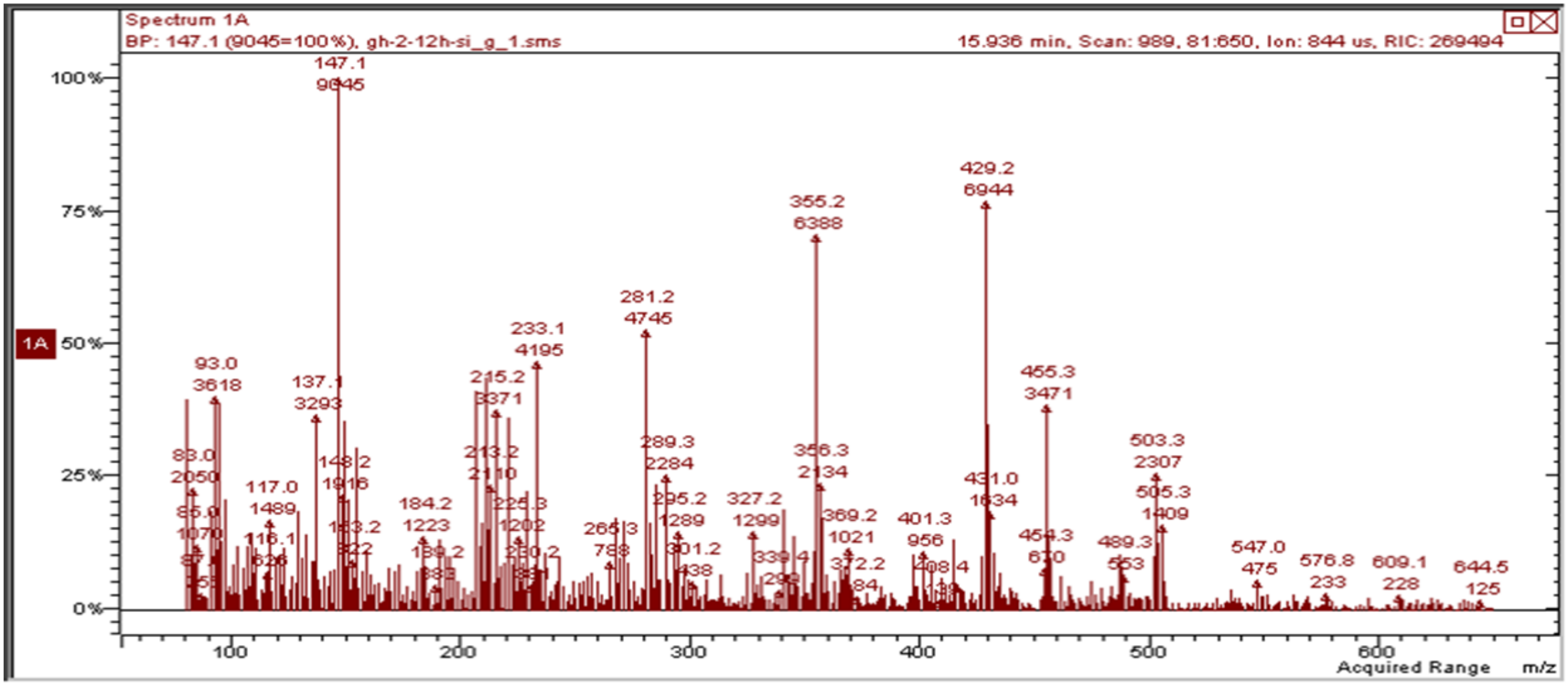

S21 Mass spectrum of the TMSi derivative of 2

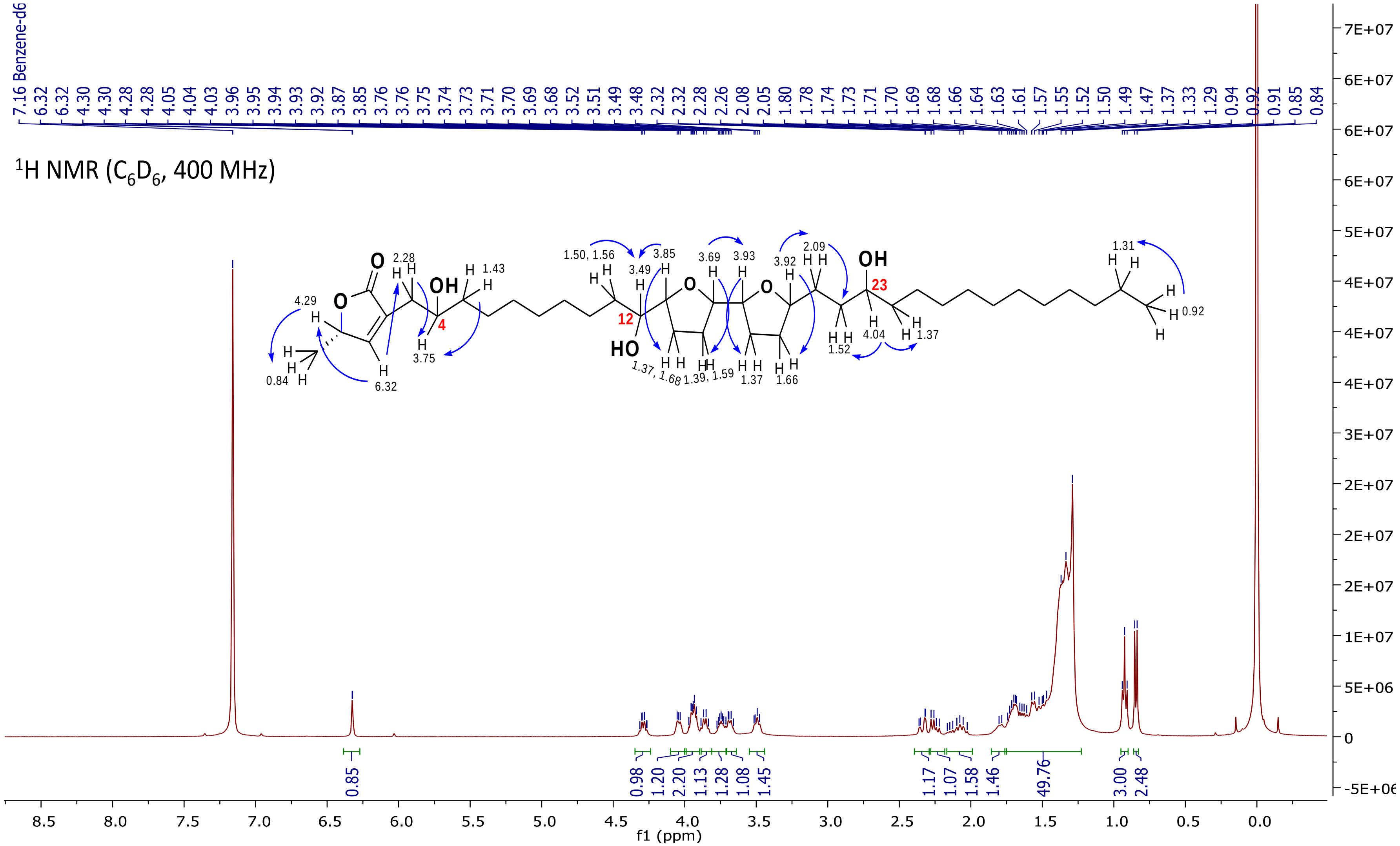

S22 Correlations in COSY and ^{1}H NMR spectrum (C_6D_6) of 2

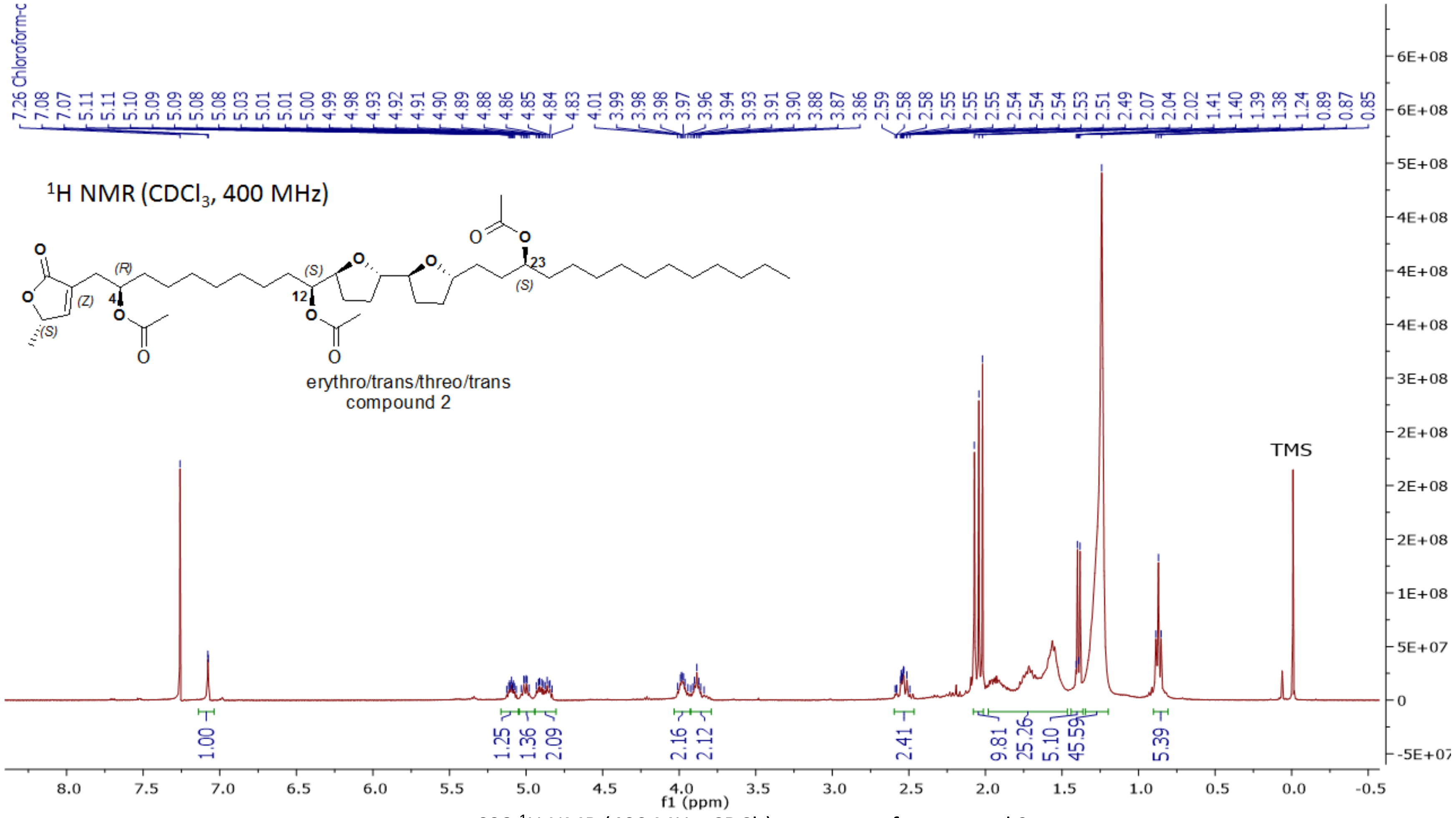

S23 ^{1}H NMR (400 MHz, $CDCl_3$) spectrum of compound 2a

COSY experiment, Mosher ester *R* ($CDCl_3$)

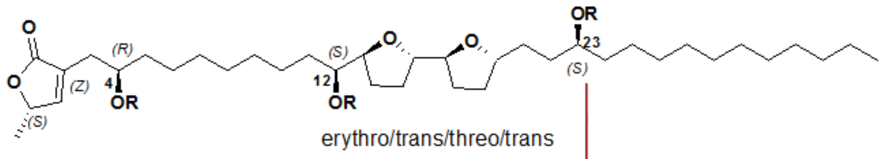

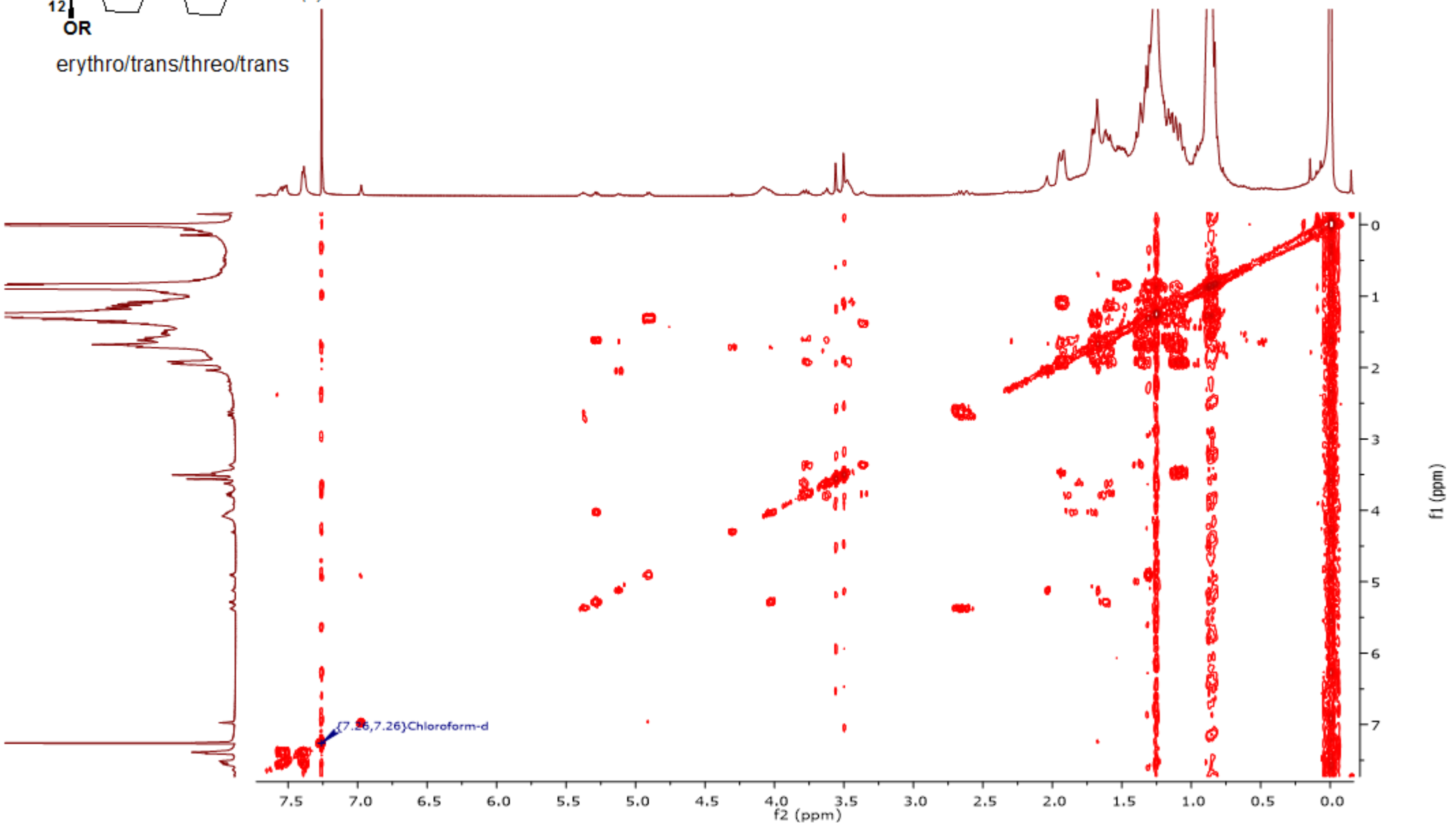

S24 COSY experiment ($CDCl_3$), Mosher ester of 2, R= (*R*)-MTPA

COSY experiment, Mosher ester *S* ($CDCl_3$)

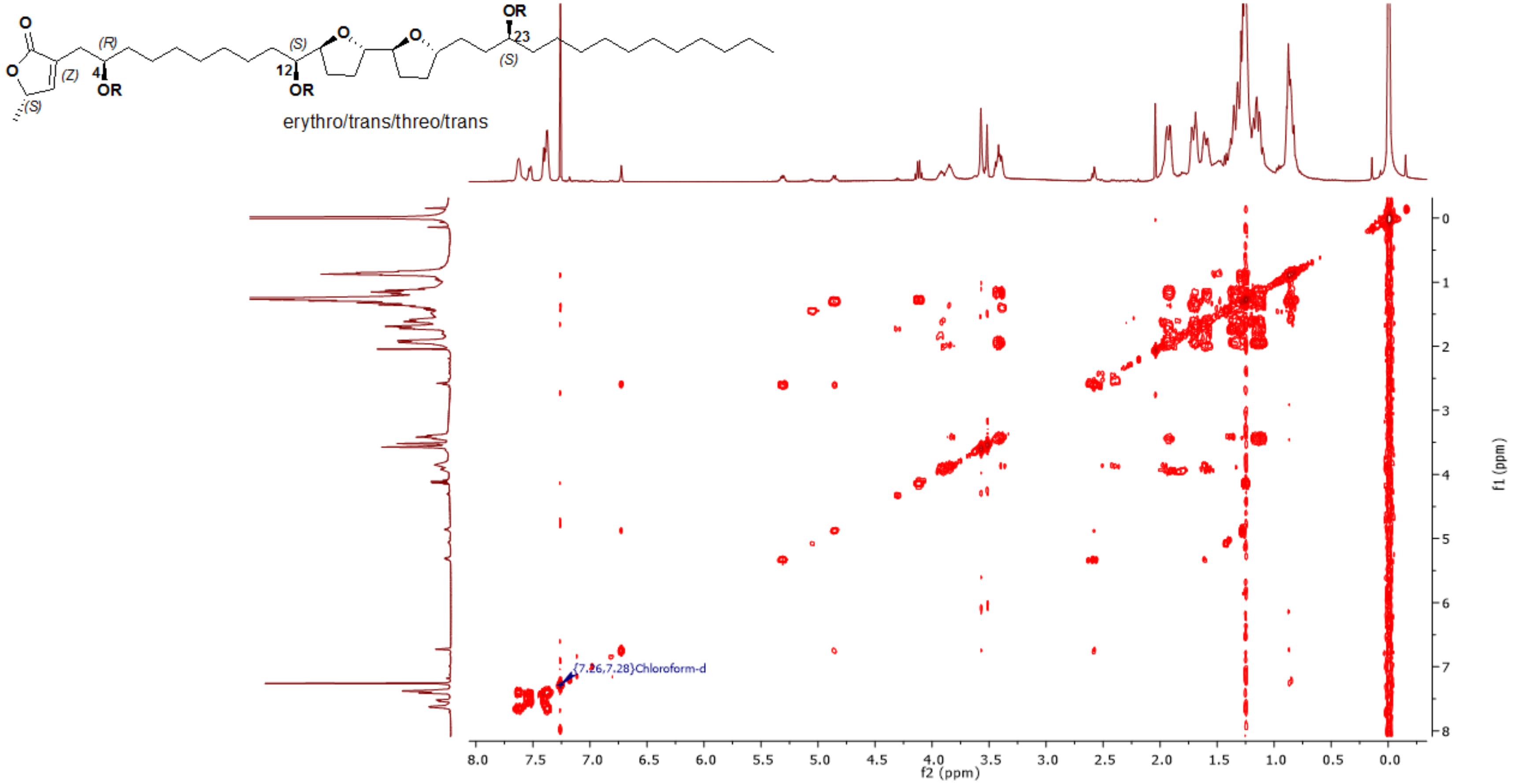

S25 COSY experiment ($CDCl_3$), Mosher ester of2, R= (S)-MTPA

Annopurpuricin B		
Experimental	6-311G(d,p)/B3LYP[a]	6-311G(d,p)/ωB97XD[b]
3414.16	3687.17	3751.55
3371.16	3621.53	3686.95
2953.01	2952.07	2954.12
2818.29	2826.32	2850.27
1738.82	1760.50	1800.79
1652.02	1633.96	1676.20
1463.96	1464.37	1462.21
1401.28	1415.27	1409.49
1371.38	1372.29	1377.12
1318.34	1323.34	1320.14
1197.79	1199.52	1200.70
1117.74	1115.44	1119.65
1071.45	1077.94	1071.42
1025.16	1025.28	1029.94
913.29	913.07	916.038
849.64	850.09	849.07
790.81	788.74	799.02
721.37	722.28	721.99
629.75	638.87	655.81
597.93	597.82	607.31
577.67	580.27	584.22

[a] Scaling factor of 0.9614
[b] Scaling factor of 0.957

S26 IR theoretical calculations of 2

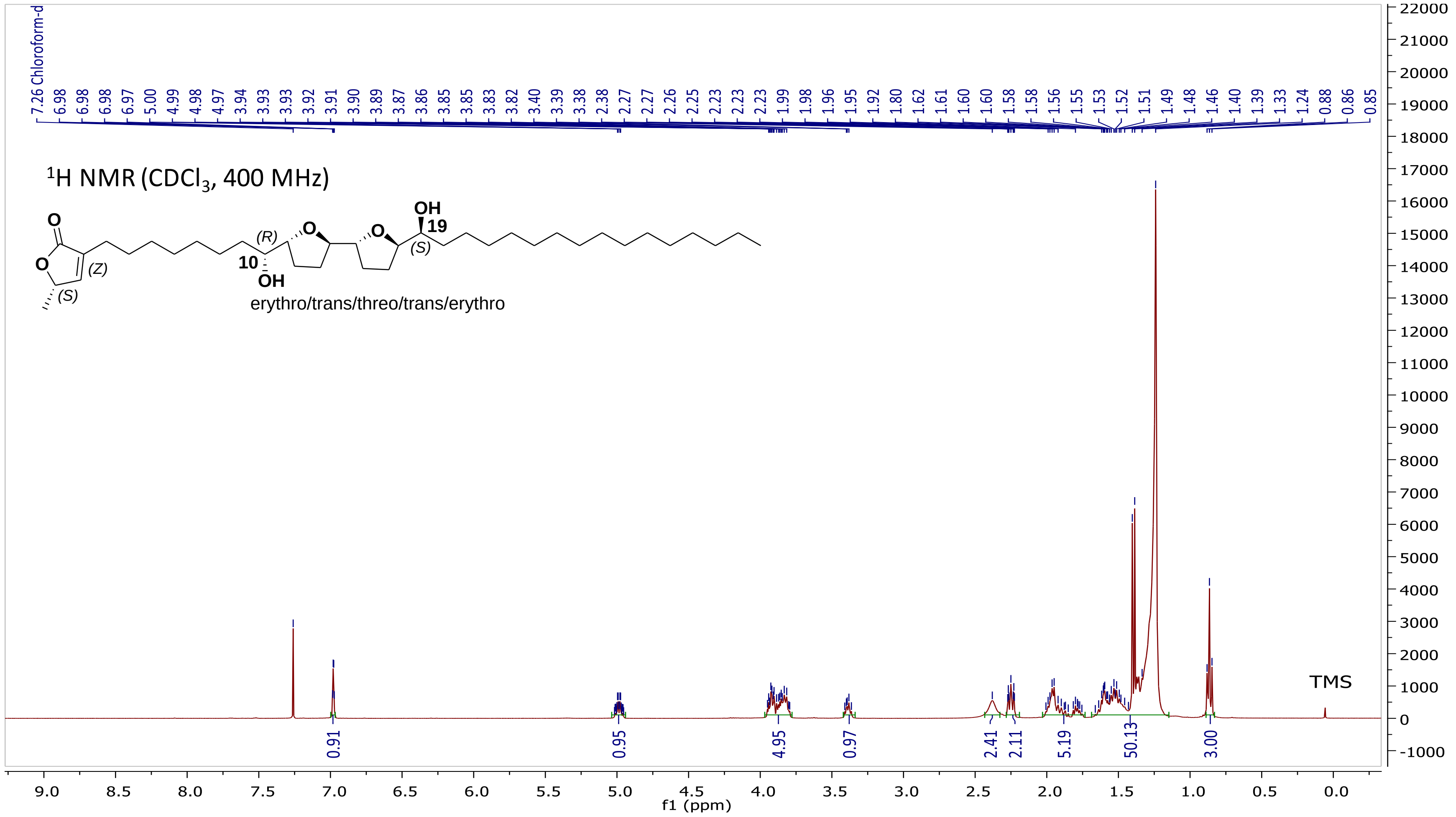

S27 ^1H NMR (400 MHz, $CDCl_3$) spectrum of compound 3

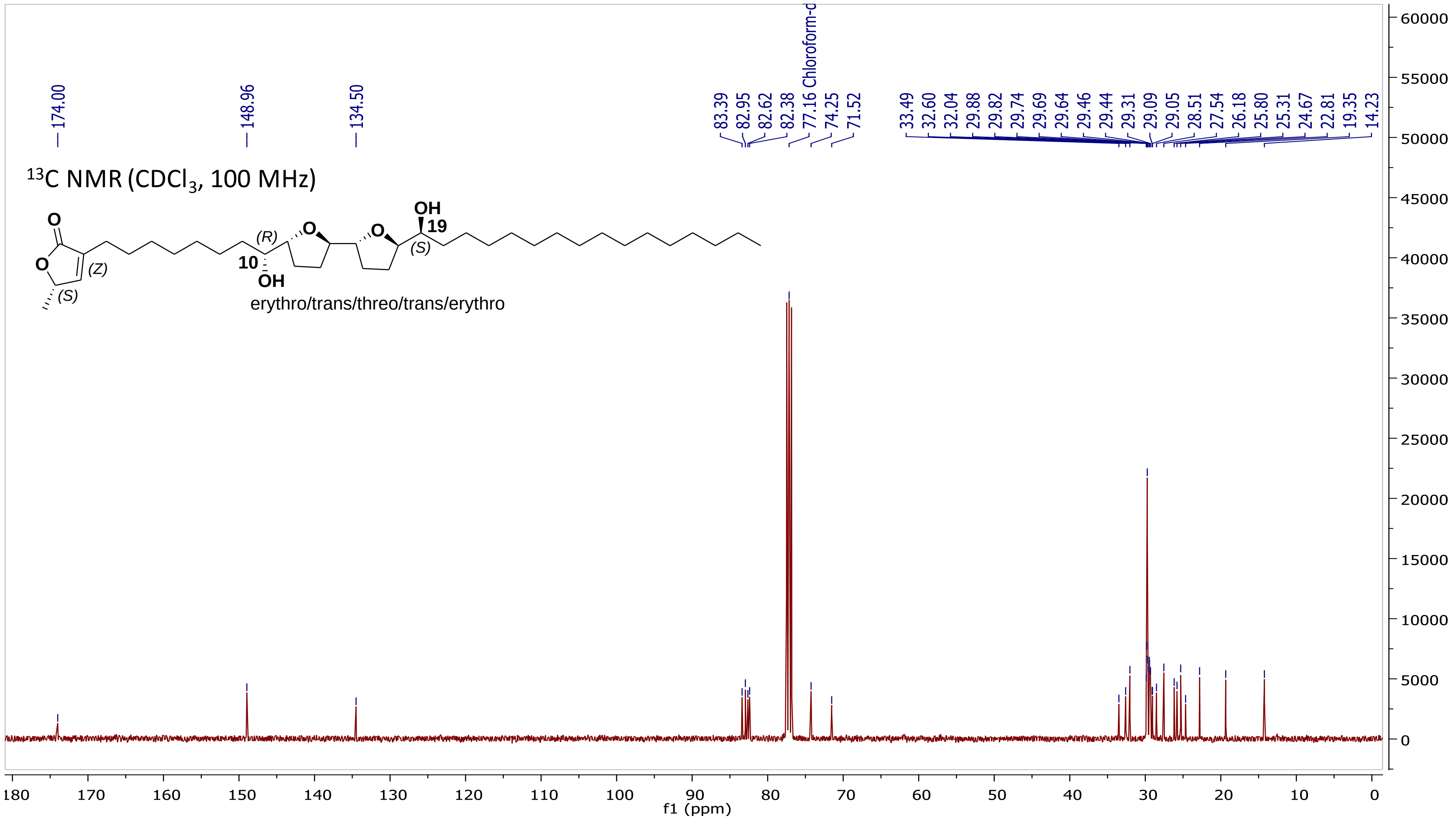

S28 ^{13}C NMR (100 MHz, $CDCl_3$) spectrum of compound 3

COSY experiment ($CDCl_3$)

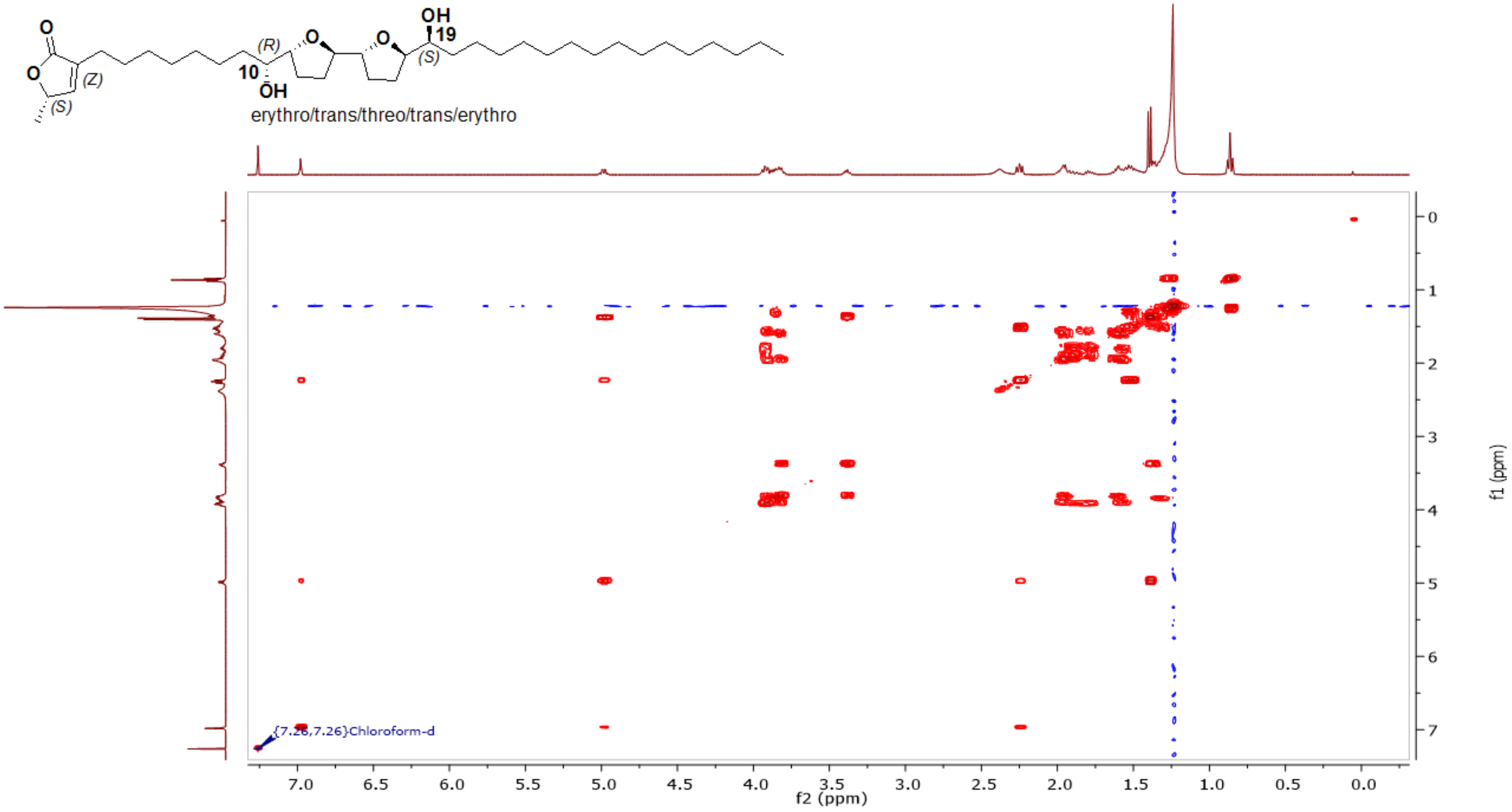

S29 COSY experiment ($CDCl_3$) of 3

HSQC experiment ($CDCl_3$)

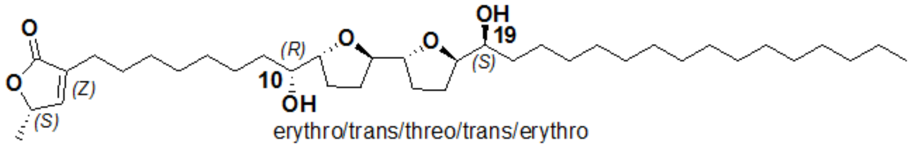

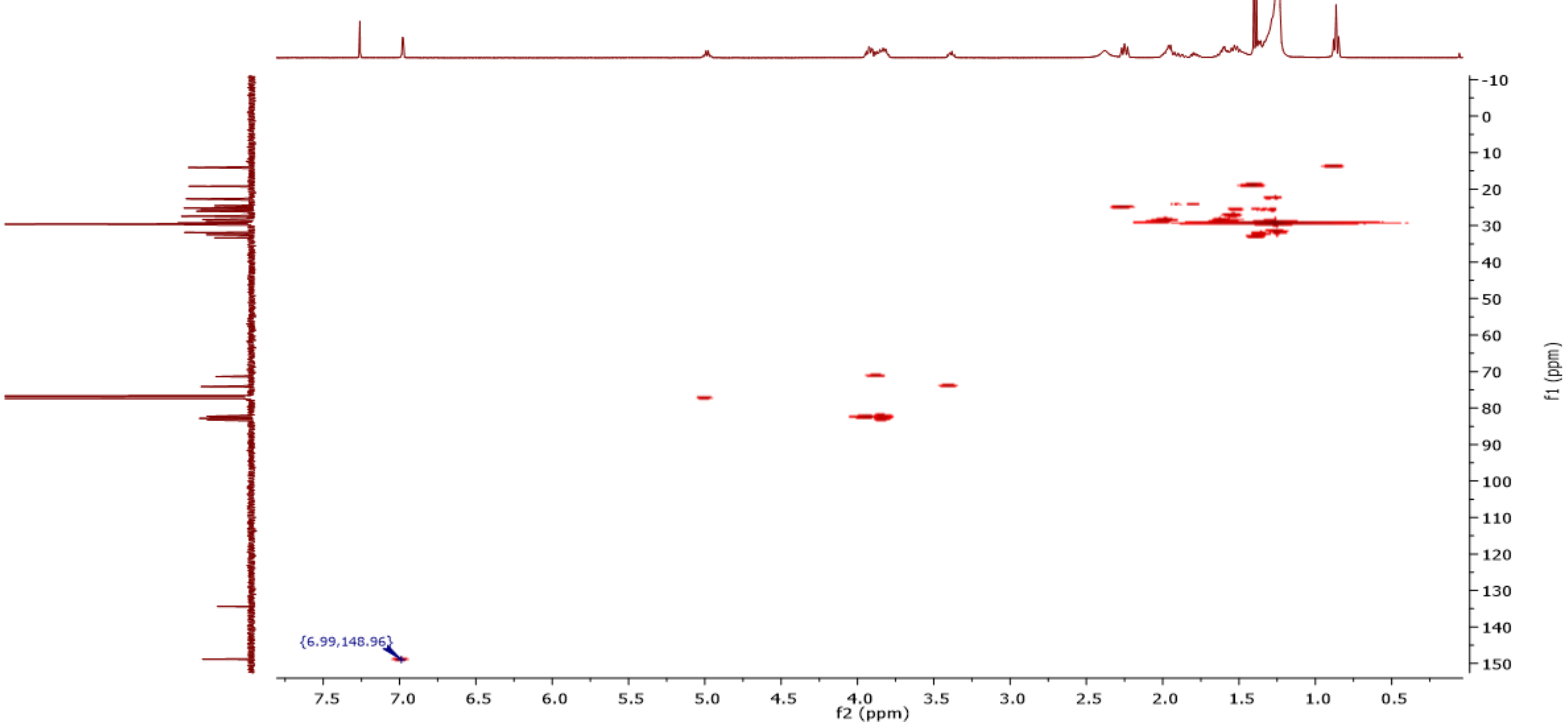

S30 HSQC experiment ($CDCl_3$) of 3

HMBC experiment ($CDCl_3$)

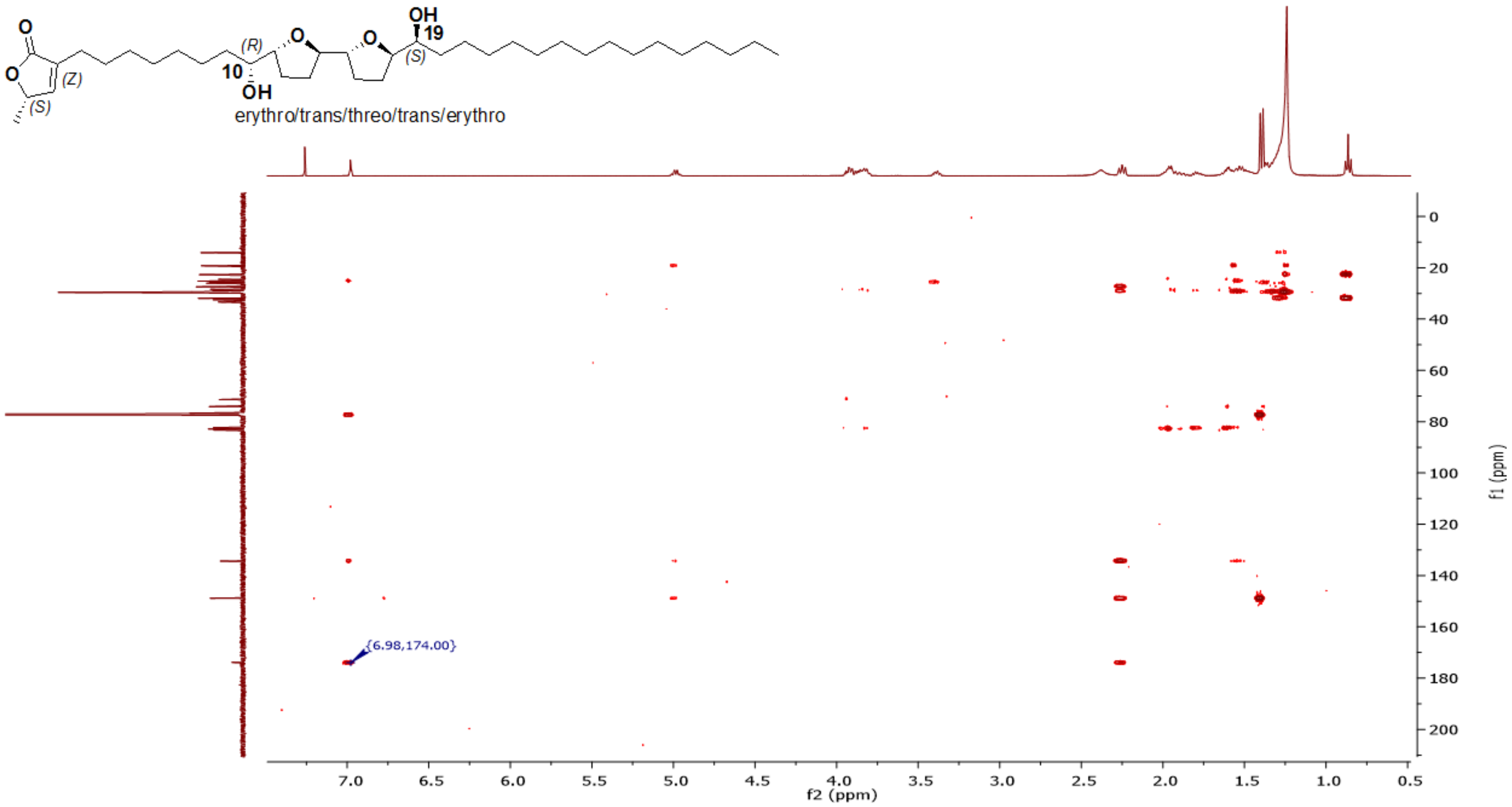

S31 HMBC experiment ($CDCl_3$) of 3

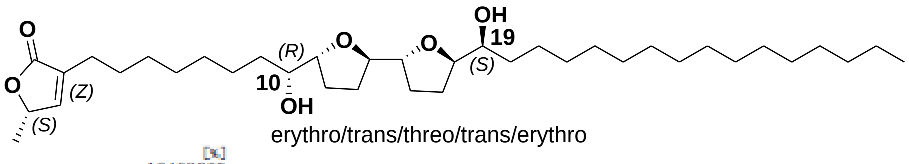

erythro/trans/threo/trans/erythro

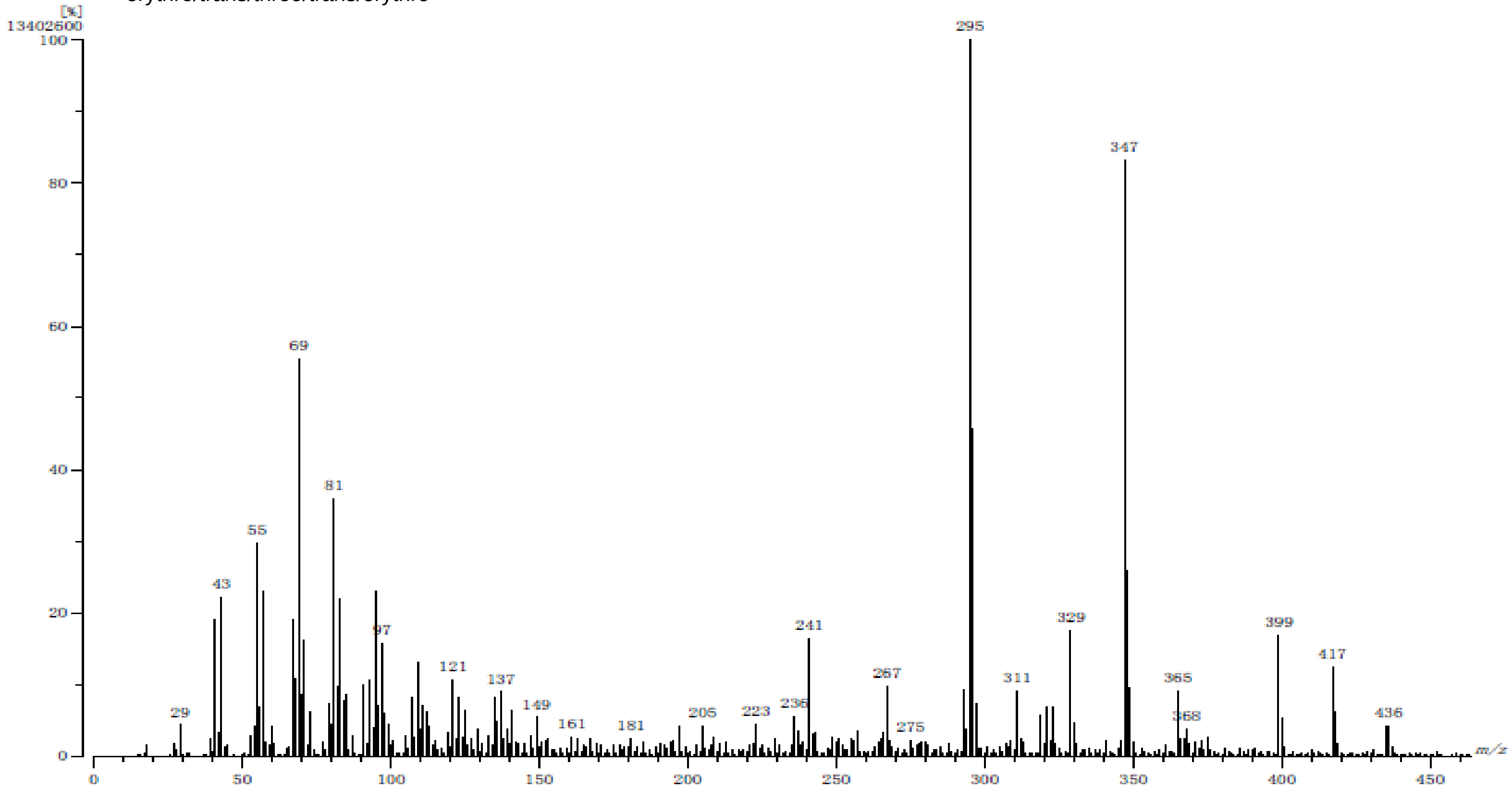

S32 Mass spectrum (IE) of 3

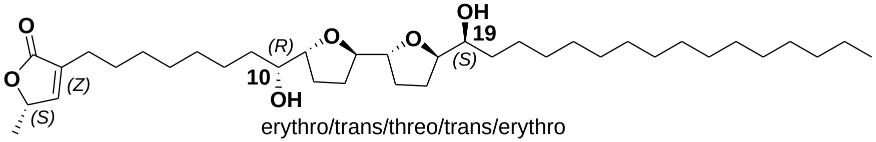

erythro/trans/threo/trans/erythro

Elemental composition calculator

Target m/z: +629.4753 amu
Tolerance: +3.0000 ppm
Result type: Elemental
Max num of results: 1000
Min DBE: -0.5000 Max DBE: +100.0000
Electron state: Even
Num of charges: 1
Add water: N/A
Add proton: N/A
File Name: J-210618-Ar-1-11-01.wiff

	Elements	Min Number	Max Number
1	C	0	50
2	H	0	70
3	N	0	2
4	O	0	10
5	Na	0	2

	Formula	Calculated m/z (amu)	mDa Error	PPM Error	DBE
1	C37 H66 O6 Na	629.475161	0.175399	0.278644	4.5

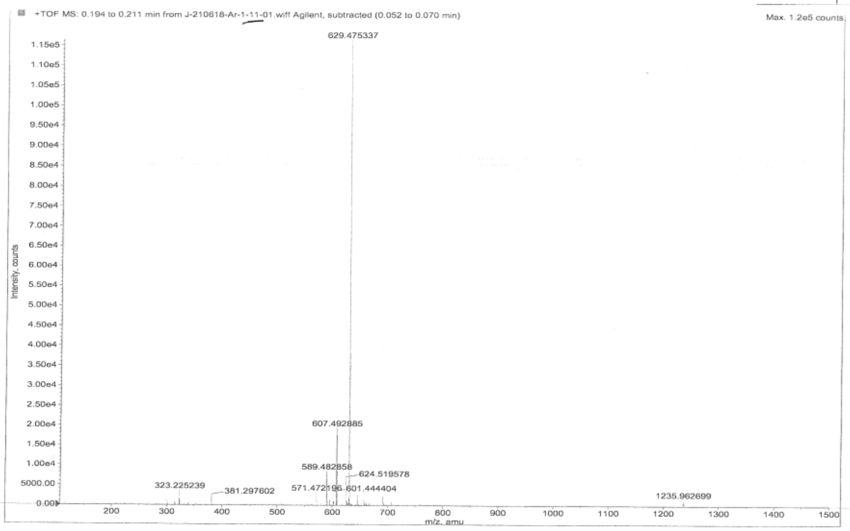

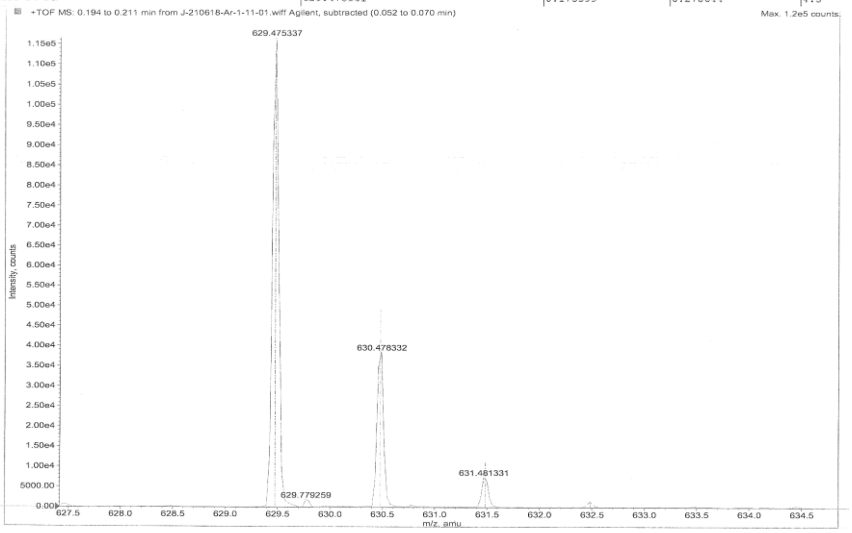

S33 HRMS (ESI-TOF) of compound 3

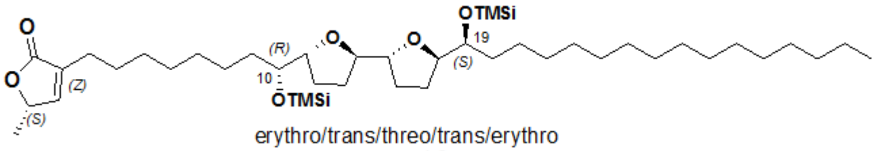

erythro/trans/threo/trans/erythro
compound 3

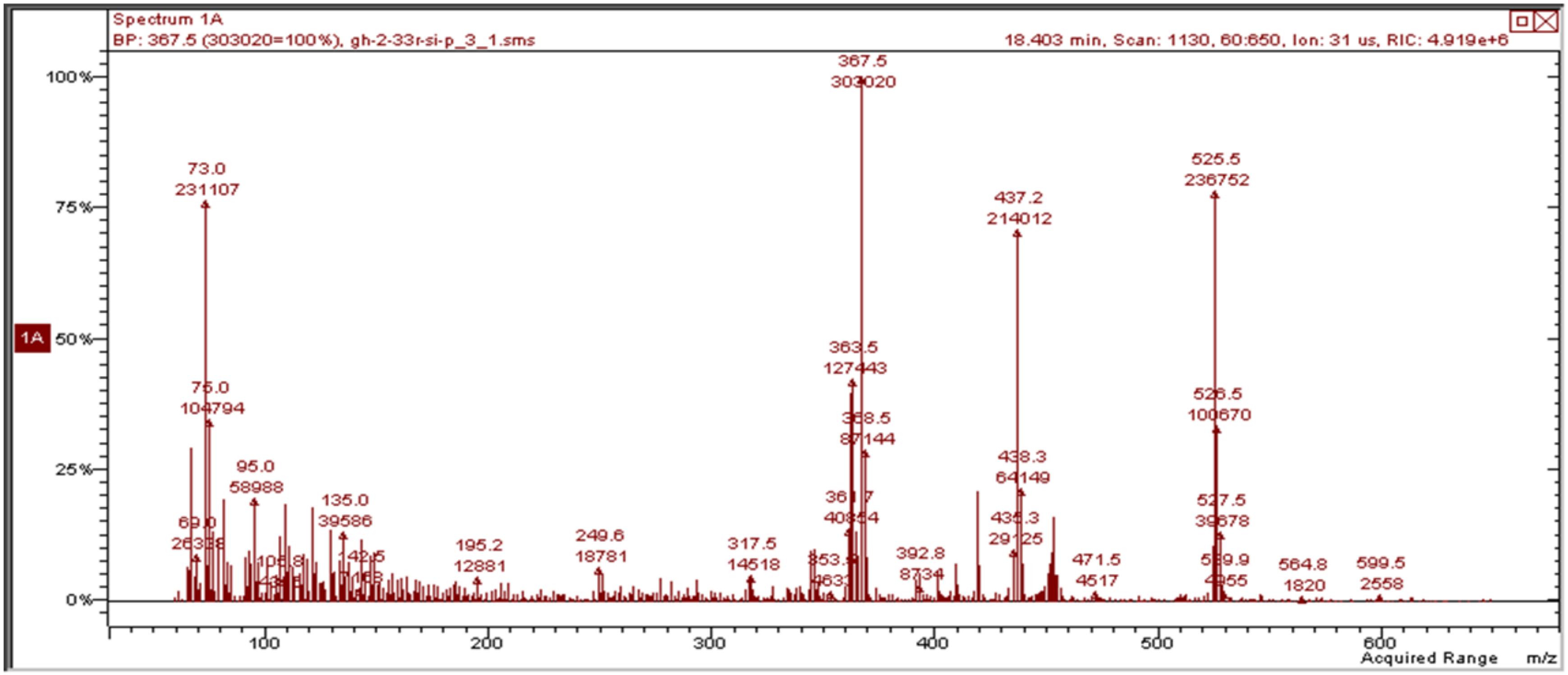

S34 Mass spectrum of the TMSi derivative of 3

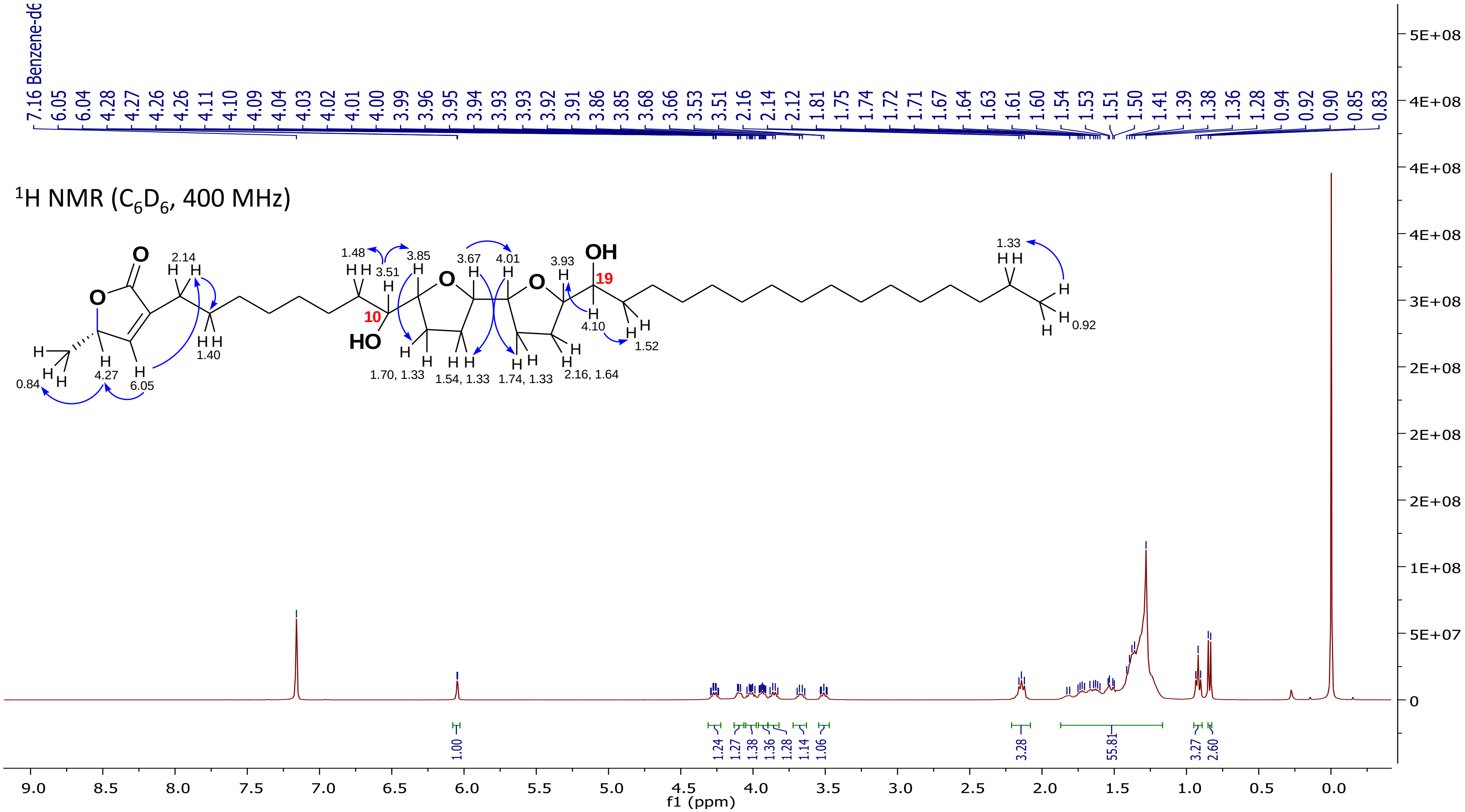

S35 Correlations in COSY and ^{1}H NMR spectrum (C_6D_6) of 3

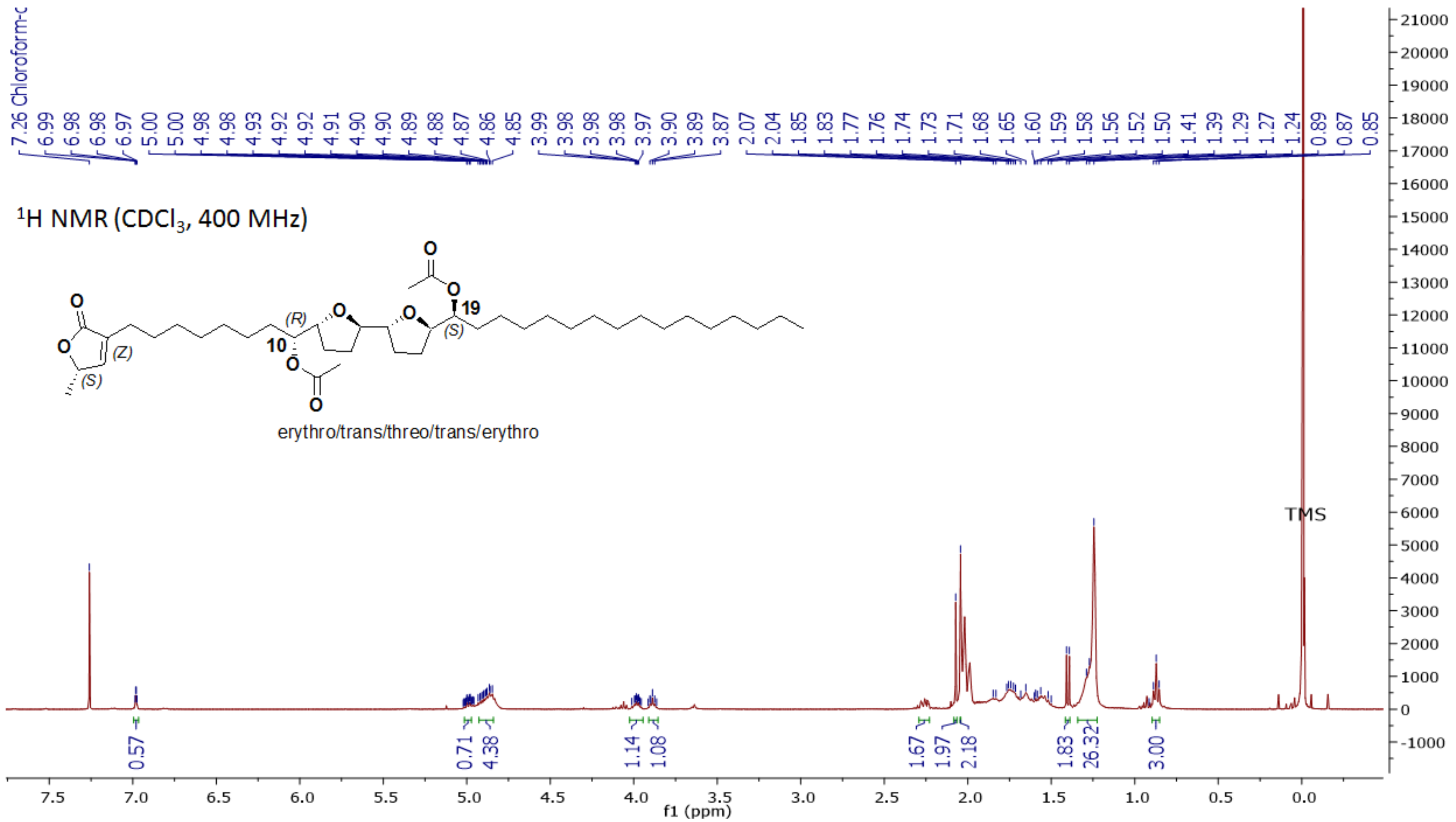

S36 ^{1}H NMR (400 MHz, $CDCl_3$) spectrum of compound 3a

COSY experiment, Mosher ester *R* ($CDCl_3$)

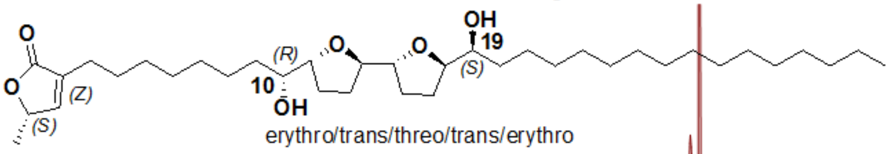

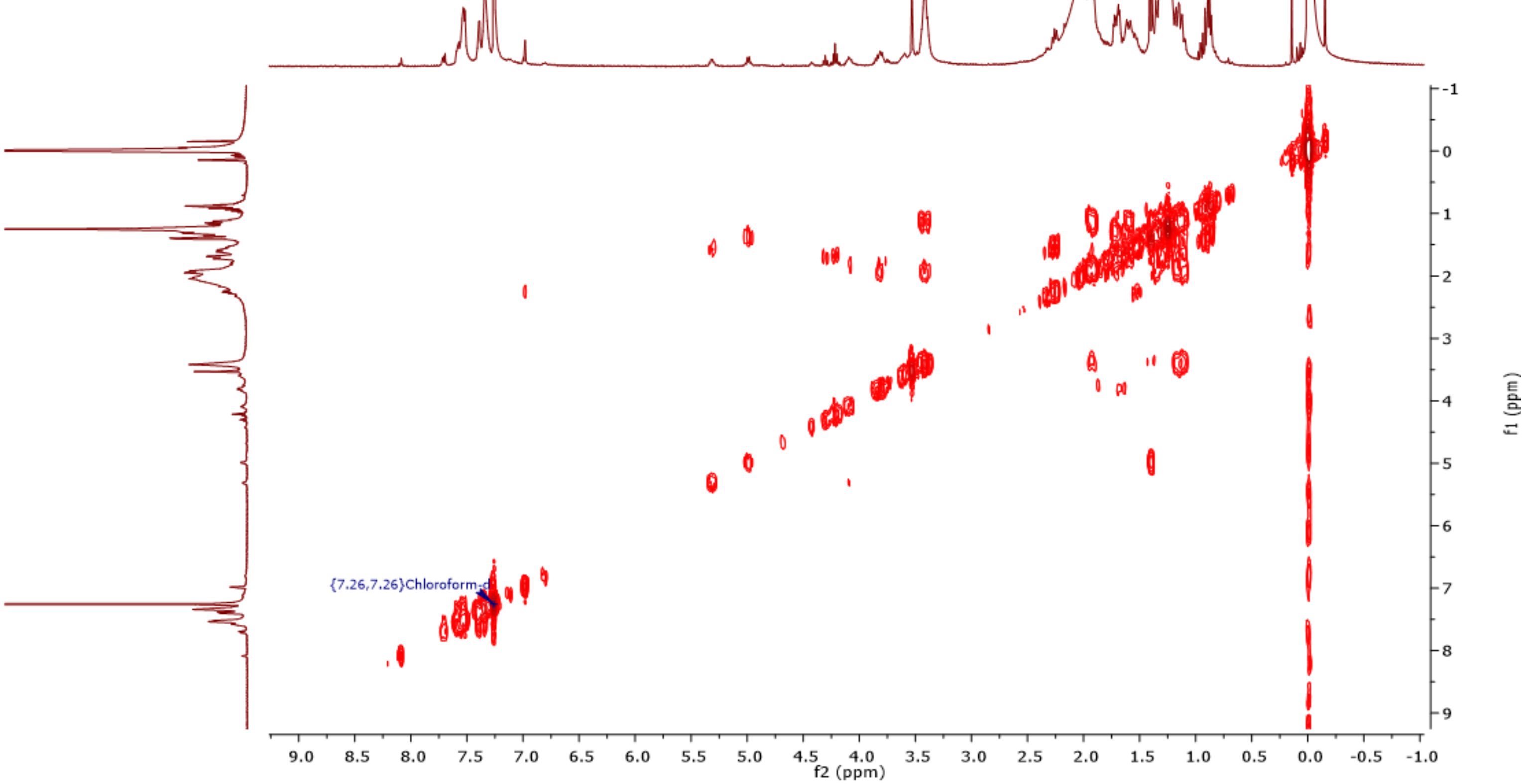

S37 COSY experiment ($CDCl_3$), Mosher ester *R* of 3

COSY experiment, Mosher ester *S* ($CDCl_3$)

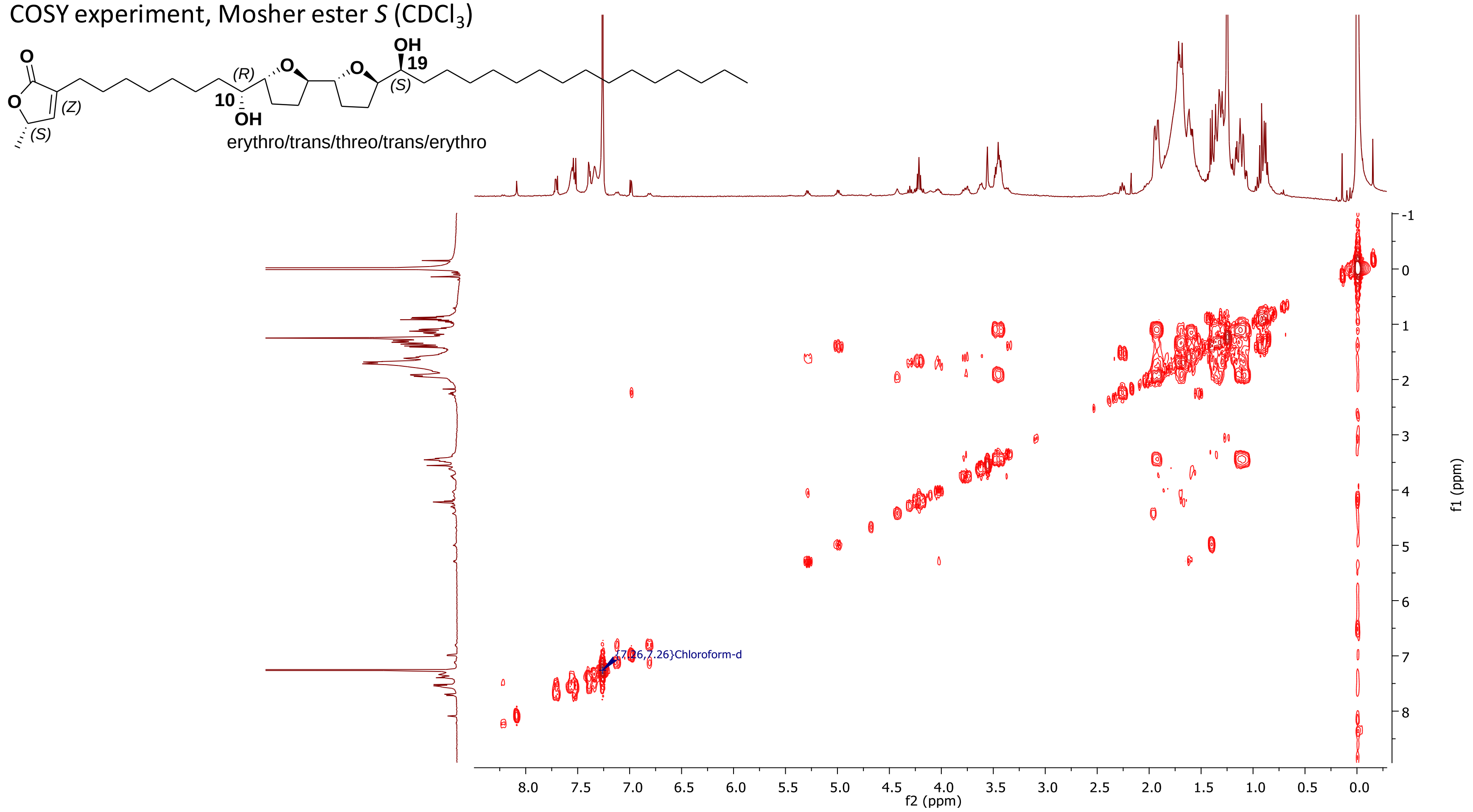

S38 COSY experiment ($CDCl_3$), Mosher ester *S* of 3

Annopurpuricin C		
Experimental	6-311G(d,p)/B3LYP[a]	6-311G(d,p)/ωB97XD[b]
3414.96	3696.59	3703.62
3373.49	3662.96	3672.85
2953.01	2957.93	2954.76
2916.36	2921.99	2916.93
2848.85	2850.15	2854.99
1742.68	1768.09	1778.42
1653.95	1641.06	1654.46
1471.68	1460.13	1457.46
1399.55	1423.16	1415.79
1314.48	1314.38	1321.17
1209.36	1212.24	1210.14
1167.89	1169.83	1170.13
1145.71	1145.47	1149.94
1111.95	1123.31	1113.32
1079.49	1083.09	1081.58
1049.27	1043.45	1048.90
1019.37	1021.23	1019.15
960.54	963.08	959.75
928.72	926.29	928.55
912.32	914.13	916.16
858.32	859.44	860.74
788.88	786.59	789.28
720.09	717.43	721.12

[a] Scaling factor of 0.9614
[b] Scaling factor of 0.957

S39 IR theoretical calculations of 3

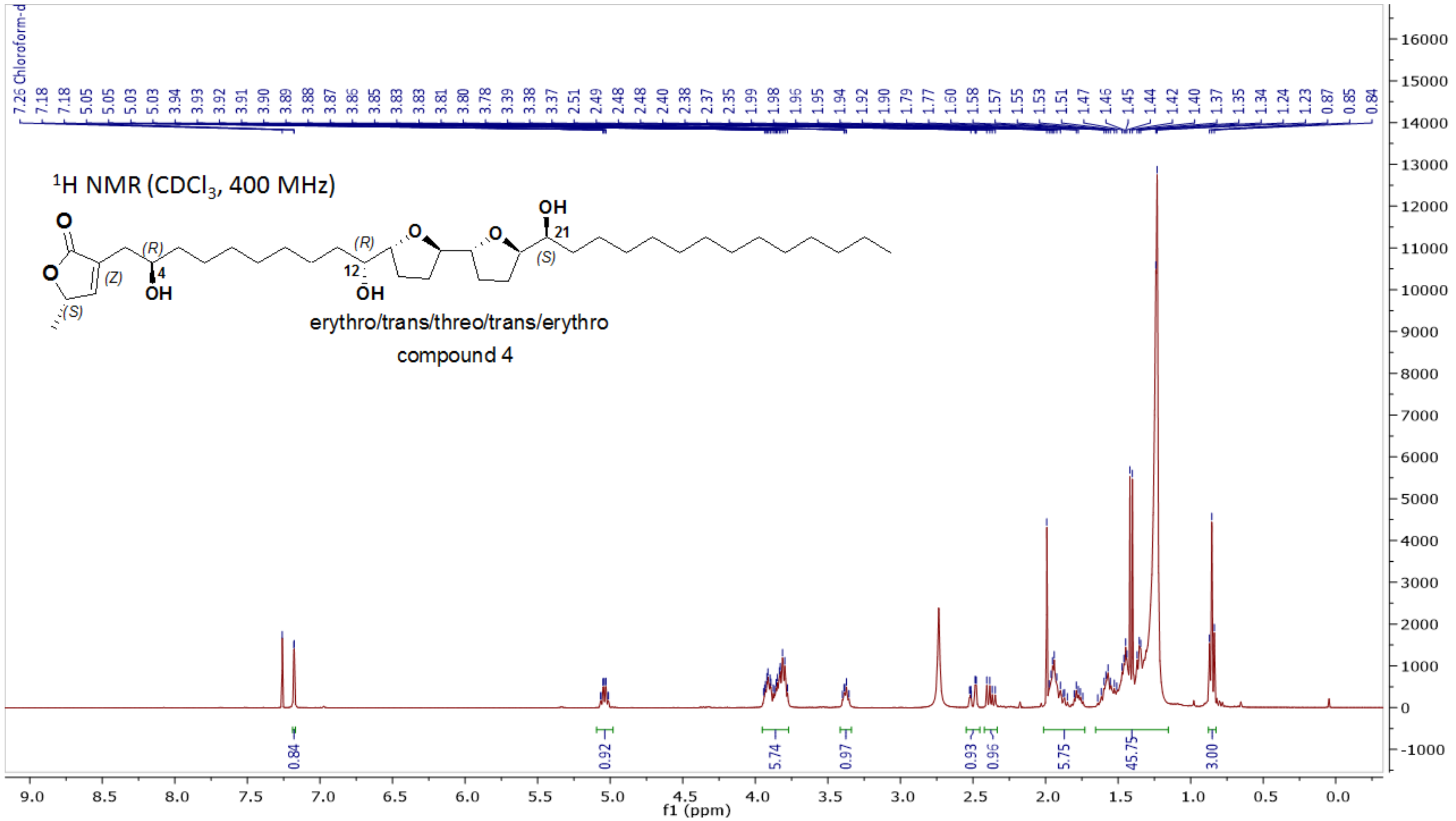

S40 ^{1}H NMR (400 MHz, $CDCl_3$) spectrum of compound 4

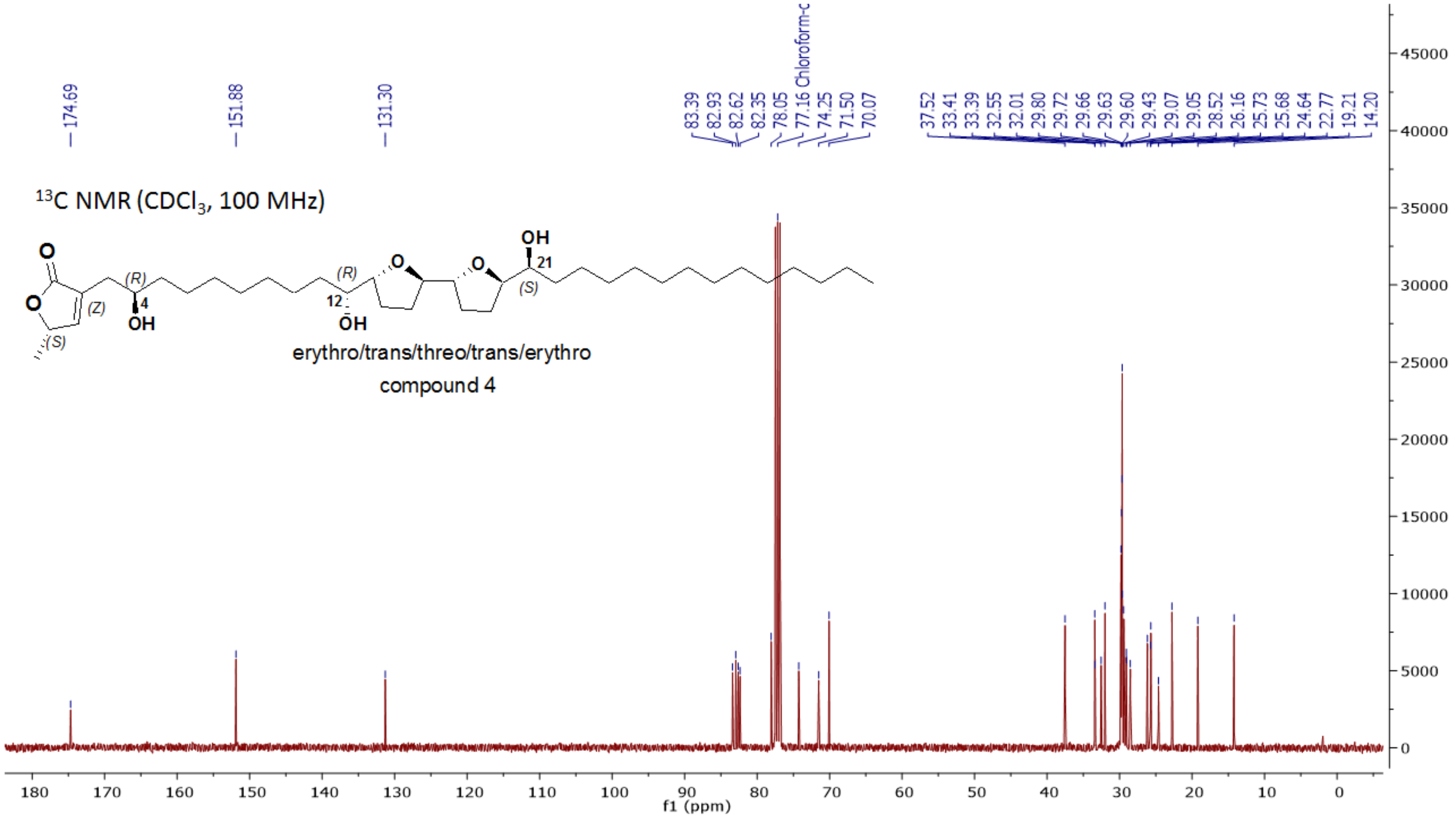

S41 ^{13}C NMR (100 MHz, $CDCl_3$) spectrum of compound 4

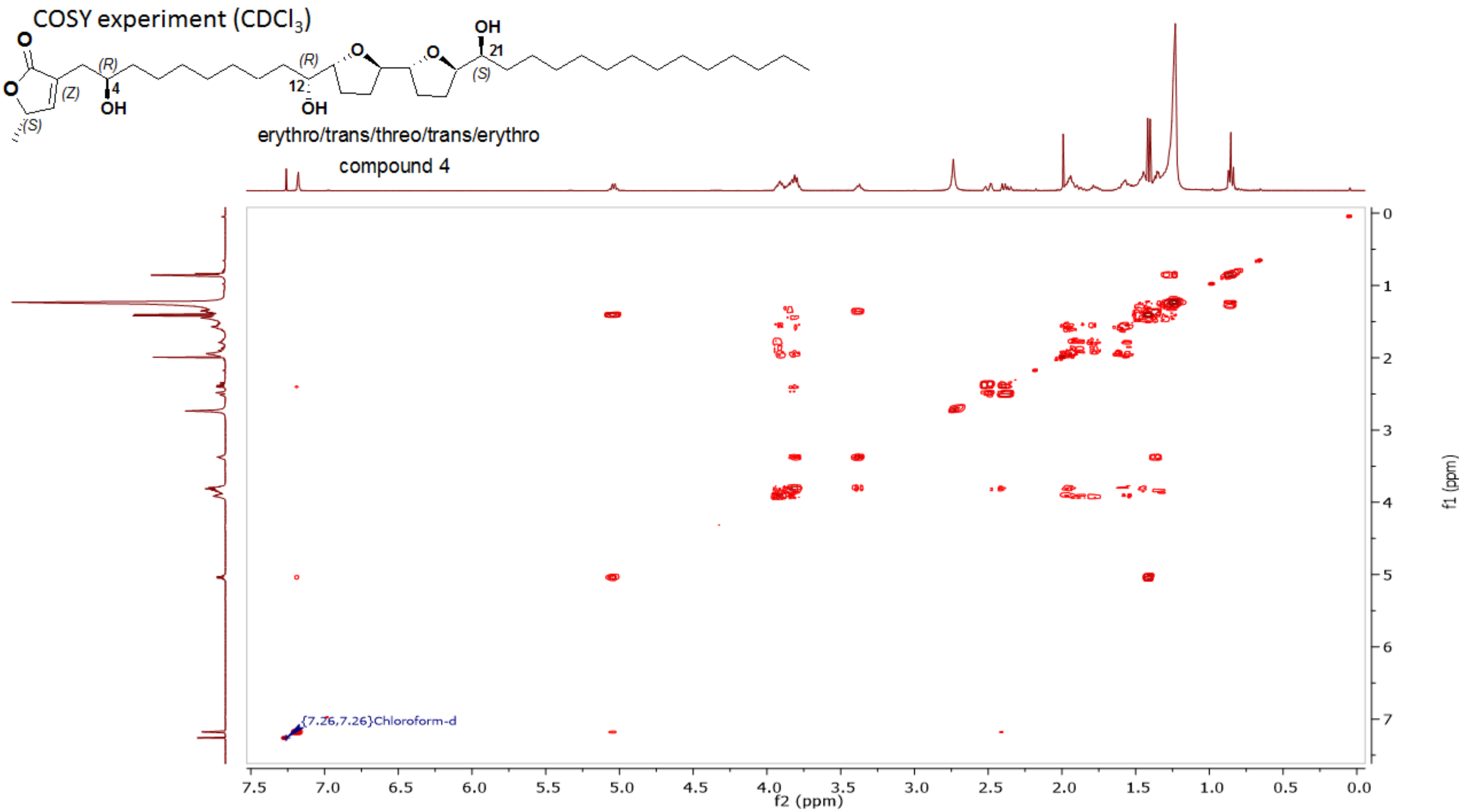

S42 COSY experiment (CDCl$_3$) of 4

HSQC experiment ($CDCl_3$)

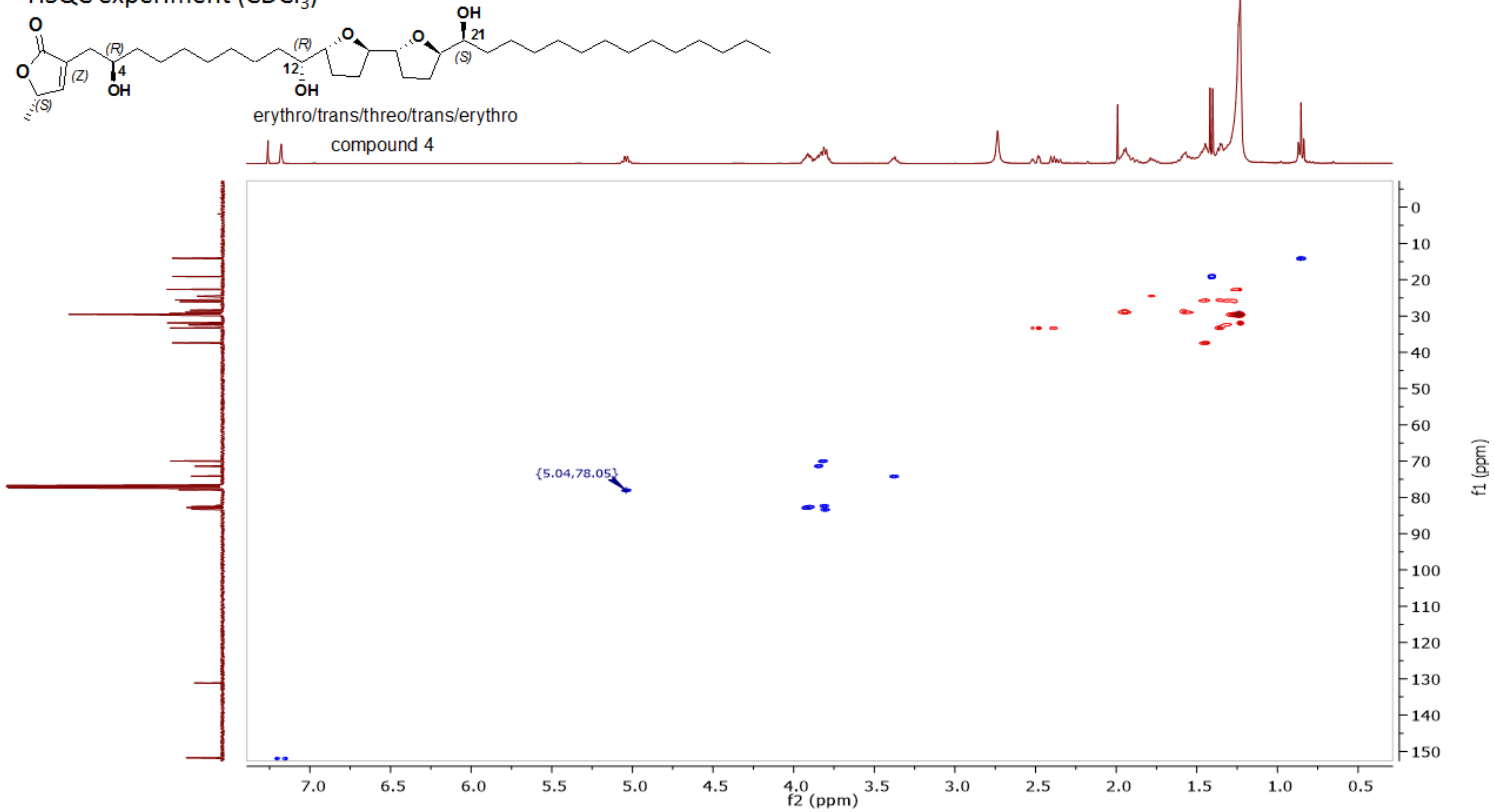

S43 HSQC experiment ($CDCl_3$) of 4

HMBC experiment ($CDCl_3$)

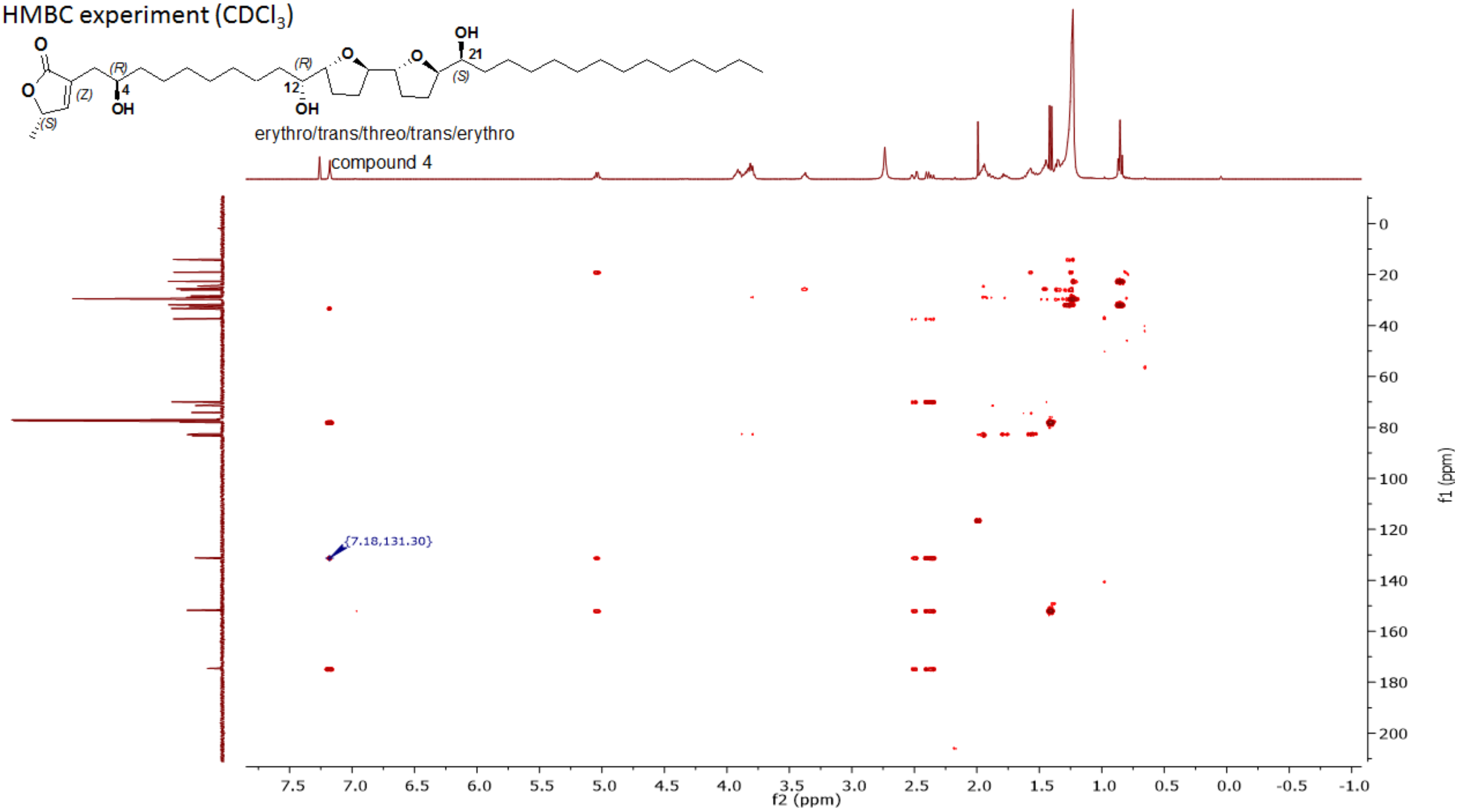

S44 HMBC experiment ($CDCl_3$) of 4

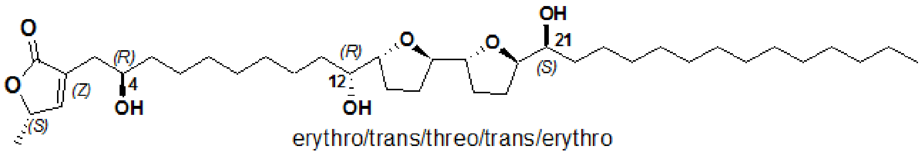

erythro/trans/threo/trans/erythro

compound 4

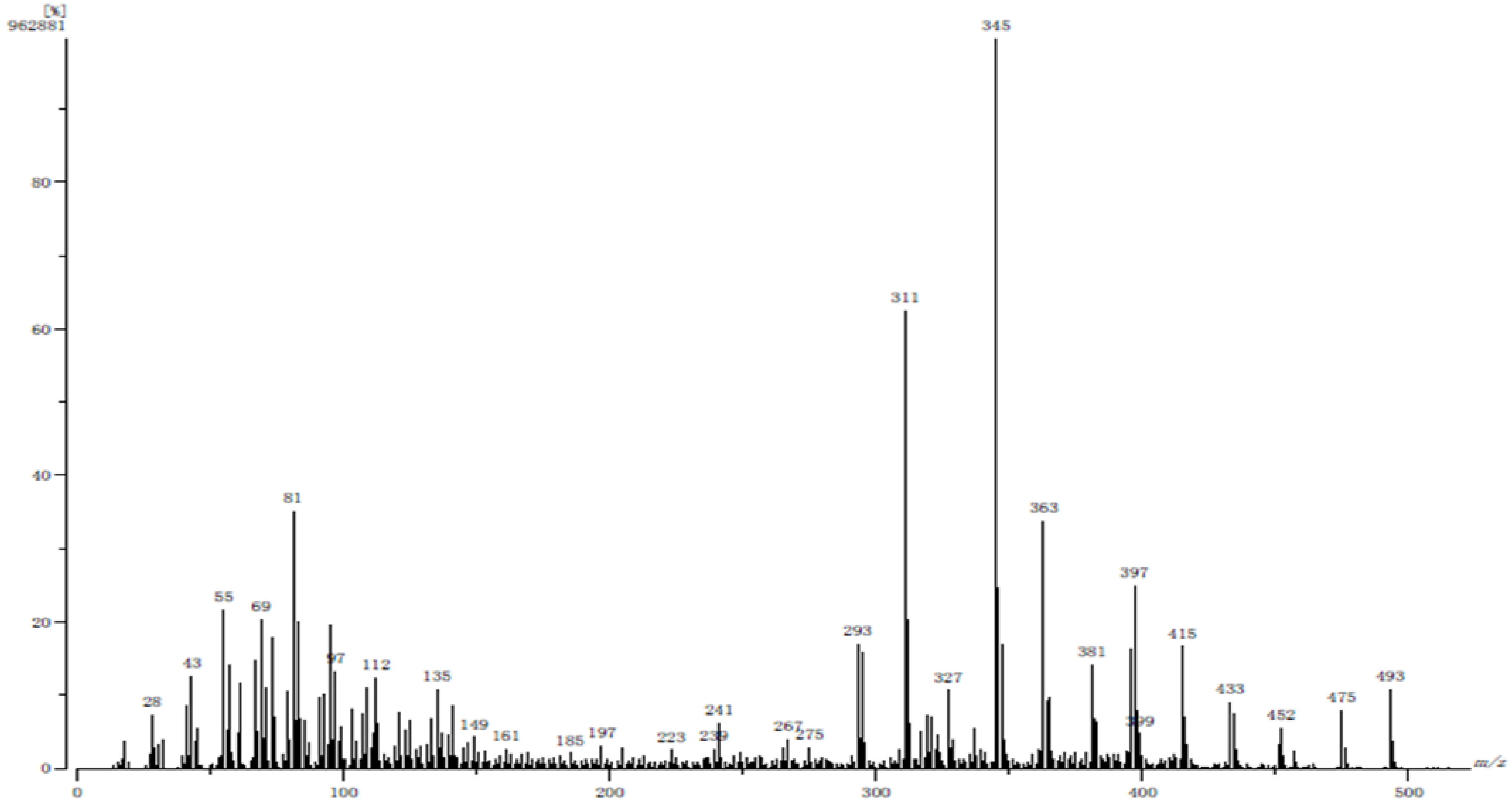

S45 Mass spectrum (IE) of 4

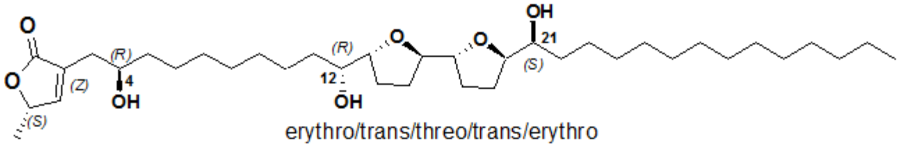

erythro/trans/threo/trans/erythro
compound 4

Elemental composition calculator

```
Target m/z:        +645.4706     amu
Tolerance:           +3.0000     ppm
Result type:       Elemental
Max num of results:      1000
Min DBE:     -0.5000     Max DBE:        +100.0000
Electron state:    Even
Num of charges:    1
Add water:         N/A
Add proton:        N/A
File Name:         J-210618-Ar-1-12-01.wiff
```

	Elements	Min Number	Max Number
1	C	0	50
2	H	0	70
3	N	0	2
4	O	0	10
5	Na	0	2

	Formula	Calculated m/z (amu)	mDa Error	PPM Error	DBE
1	C37 H66 O7 Na	645.470076	0.513759	0.795945	4.5

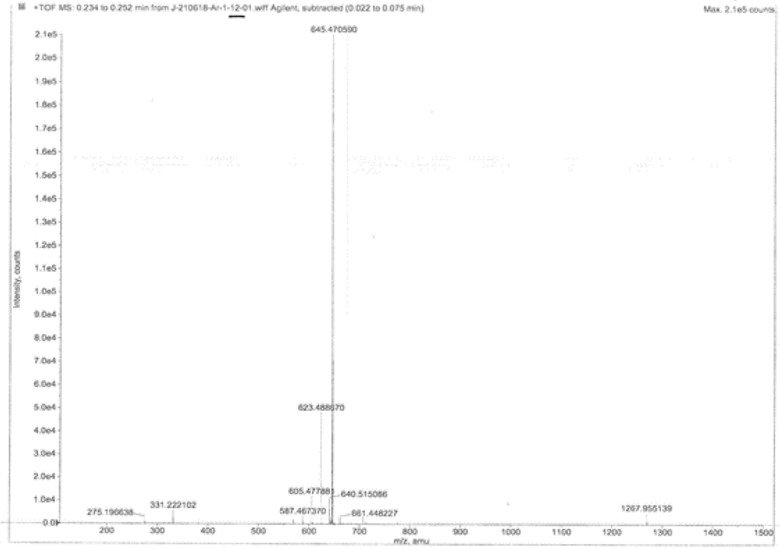

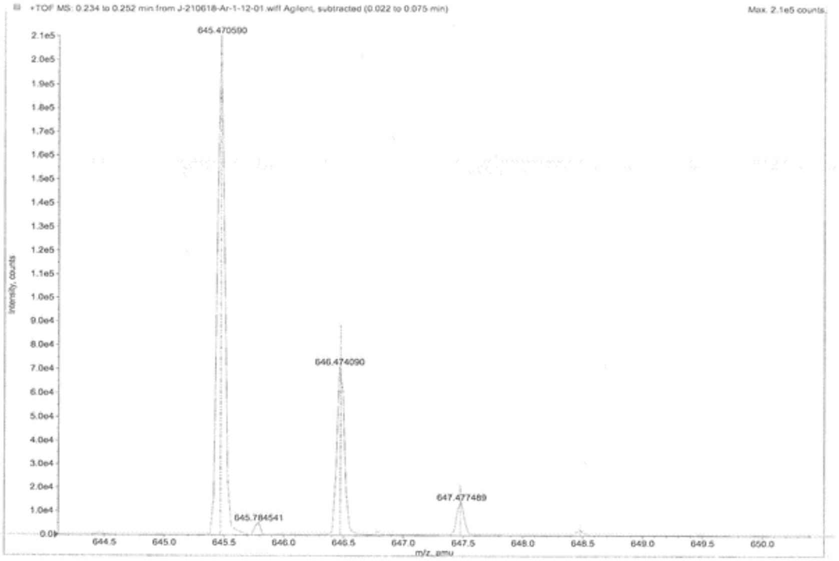

S46 HRMS (ESI-TOF) of compound 4

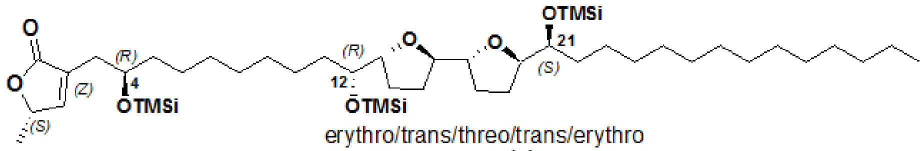

erythro/trans/threo/trans/erythro
compound 4

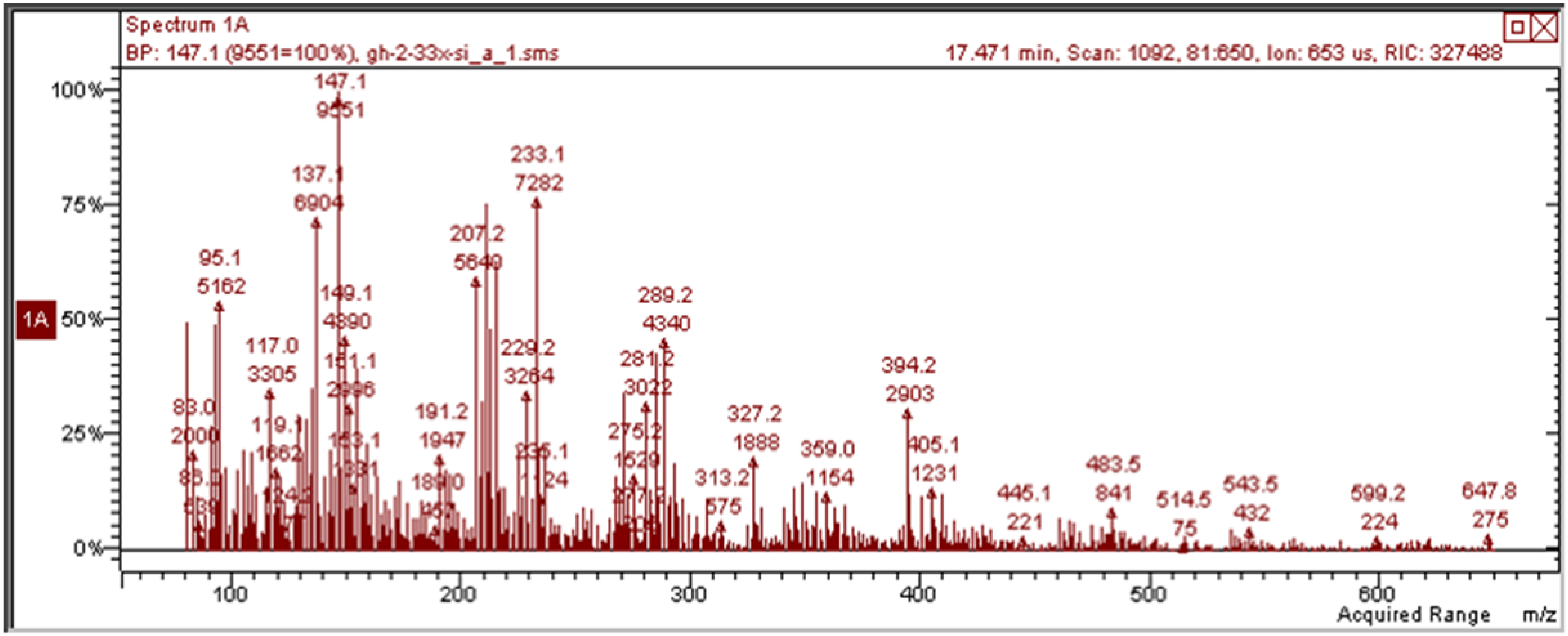

S47 Mass spectrum of the TMSi derivative of 4

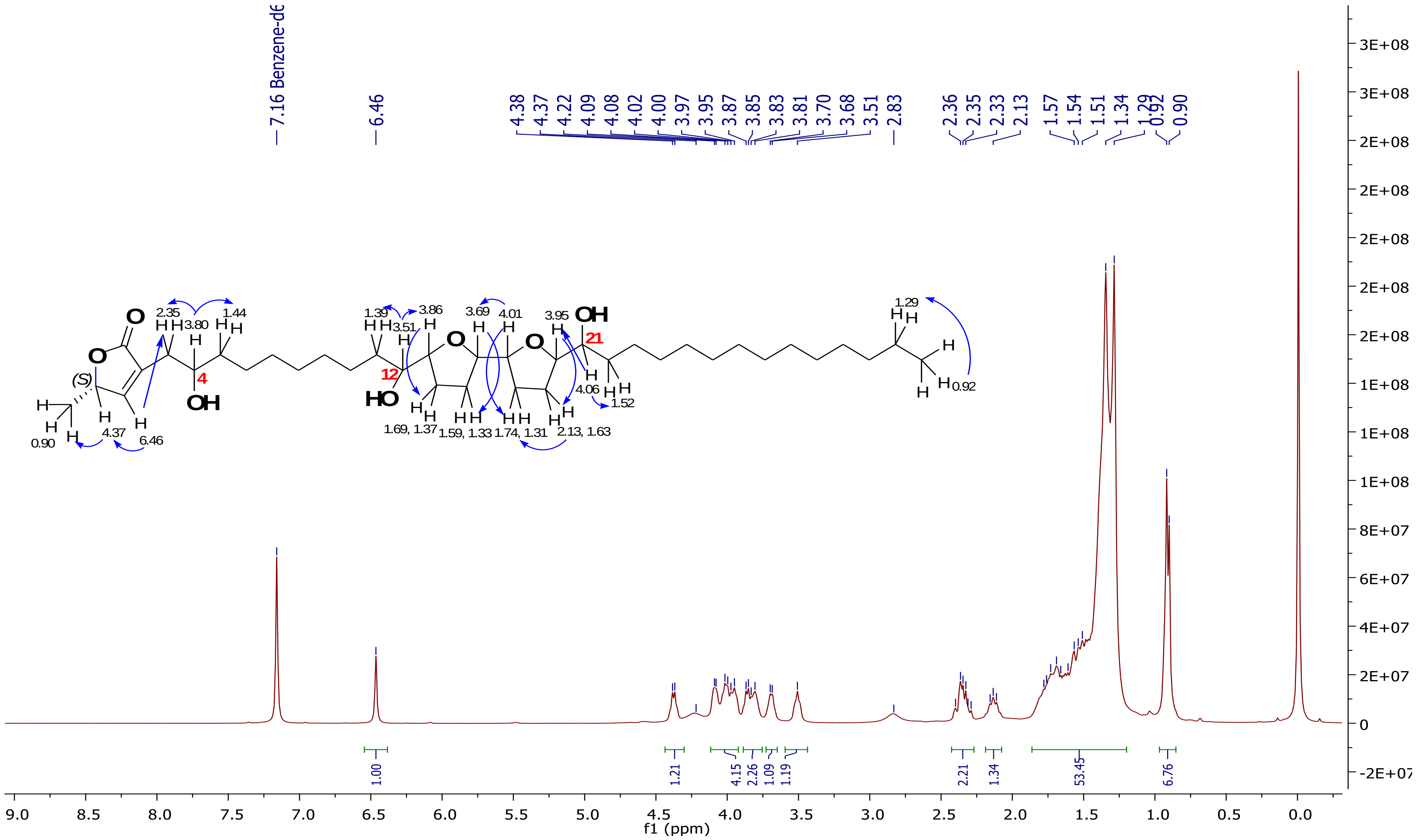

S48 Correlations in COSY and ^{1}H NMR spectrum (C_6D_6) of 4

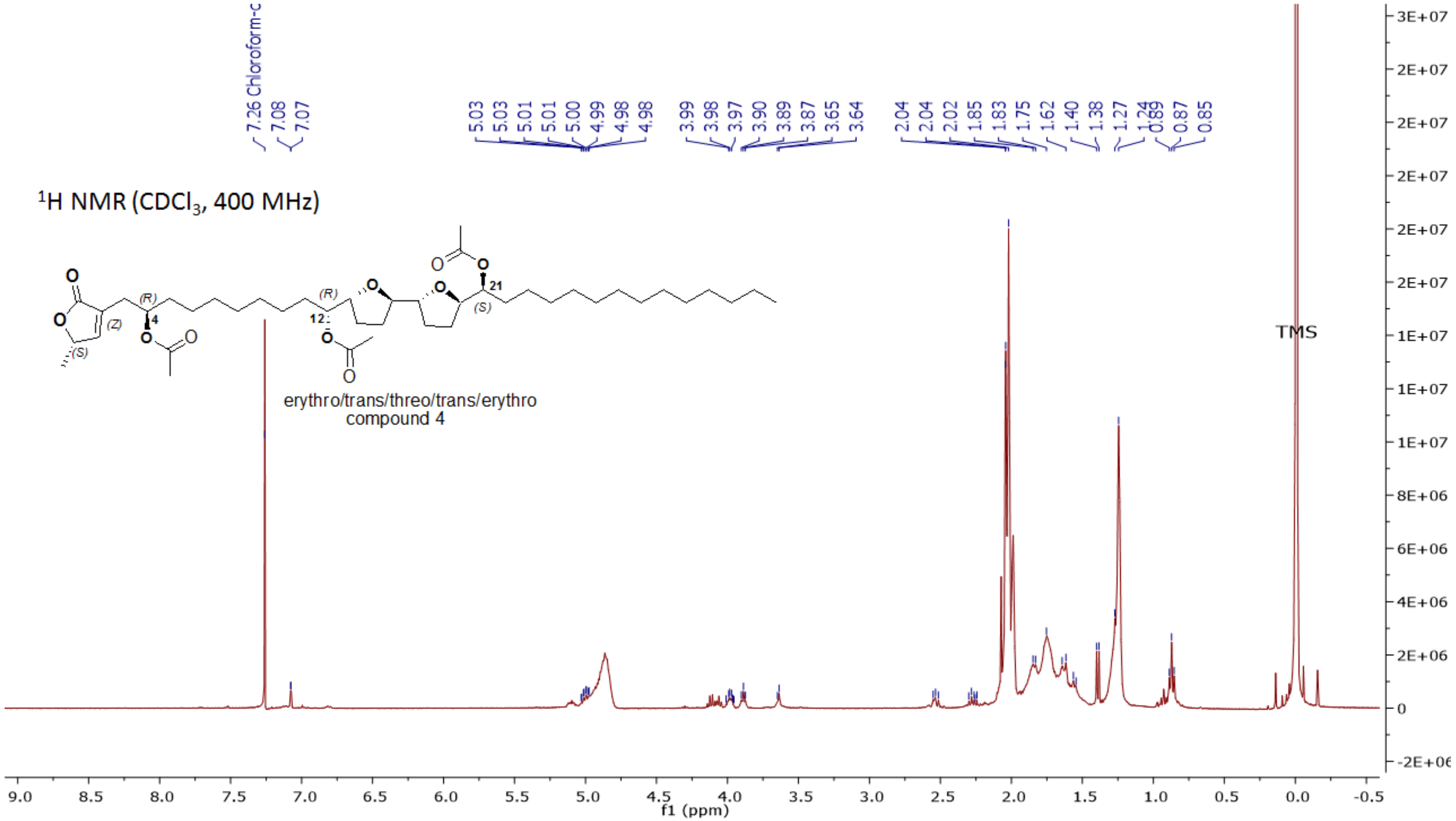

S49 ^{1}H NMR (400 MHz, $CDCl_3$) spectrum of compound 4a

COSY experiment, Mosher ester *R* ($CDCl_3$)

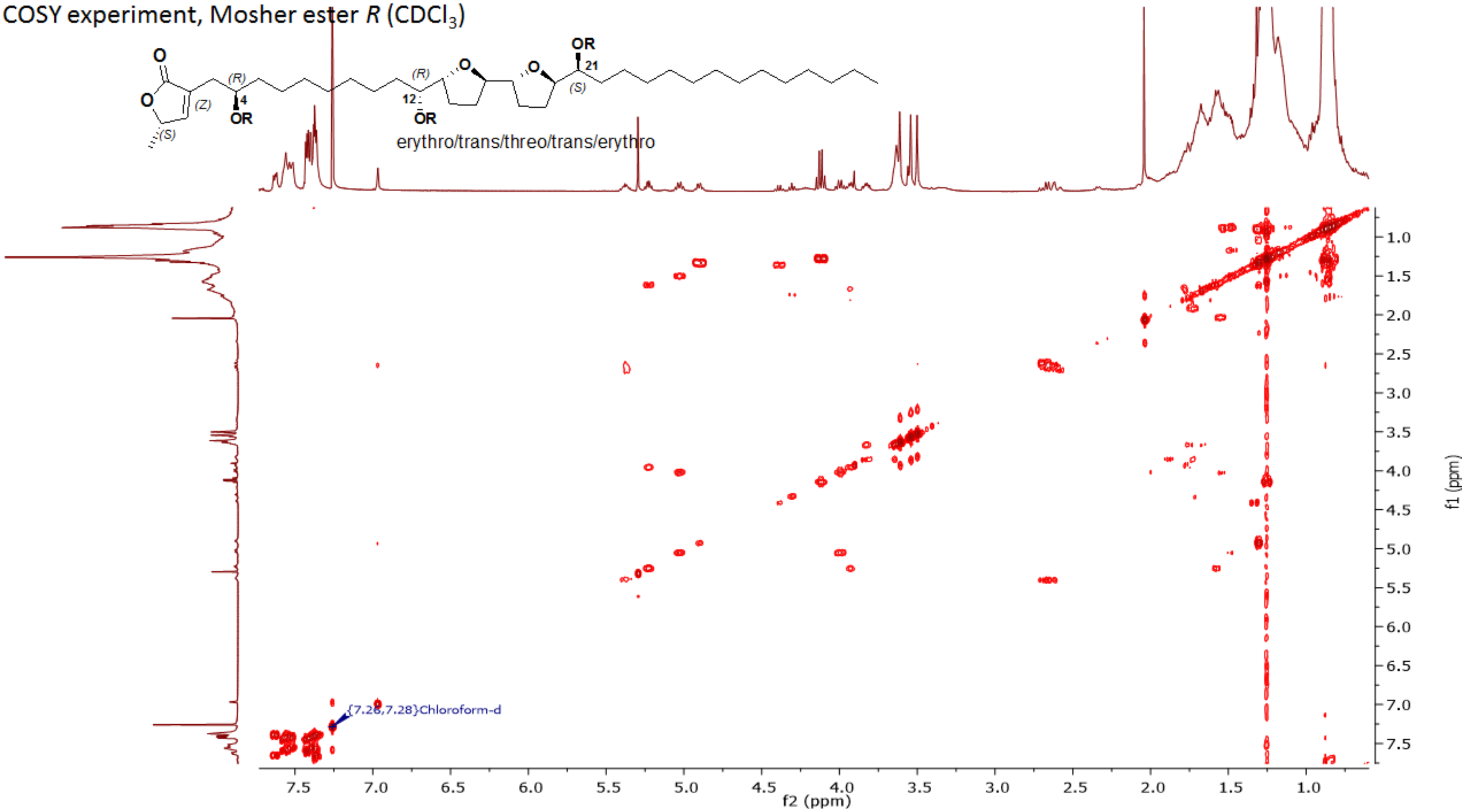

S50 COSY experiment ($CDCl_3$), Mosher ester of 4, R= (*R*)-MTPA

COSY experiment, Mosher ester *S* ($CDCl_3$)

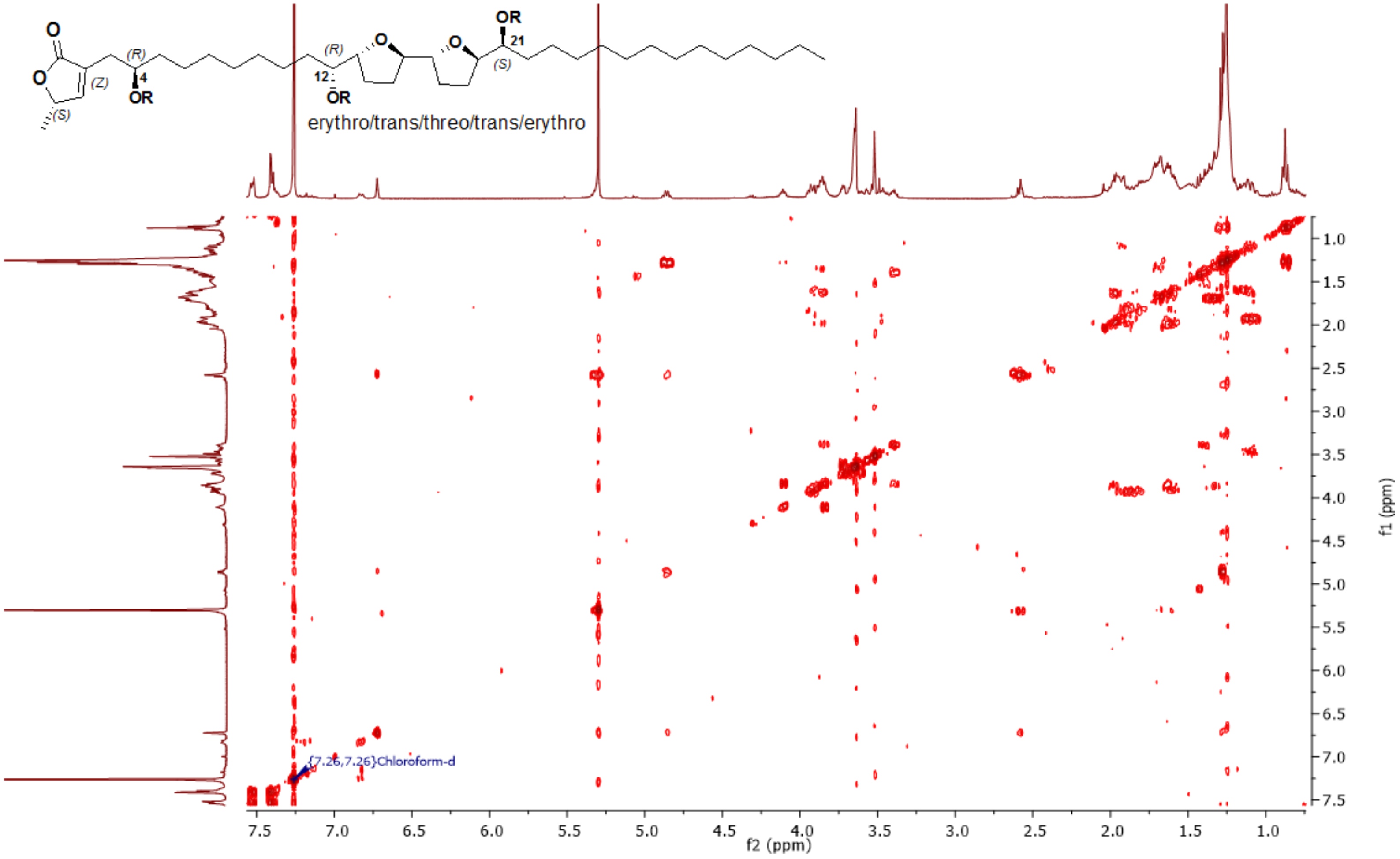

S51 COSY experiment ($CDCl_3$), Mosher ester of 4, R= (*S*)-MTPA

Annopurpuricin D		
Experimental	6-311G(d,p)/ B3LYP[a]	6-311G(d,p)/ωB97XD[b]
3416.88	3664.88	3773.30
3367.70	3662.04	3730.17
2953.01	2954.30	2953.75
2917.32	2920.79	2920.97
2849.81	2857.83	2872.94
1747.50	1752.92	1789.45
1652.99	1636.44	1675.74
1464.93	1460.32	1466.60
1399.35	1417.90	1399.07
1374.27	1373.76	1376.77
1319.30	1318.70	1320.91
1202.61	1202.51	1201.12
1118.71	1114.02	1111.31
1074.34	1074.07	1076.38
1025.16	1025.42	1034.49
960.54	961.00	961.66
912.32	920.32	910.19
857.37	862.66	858.16
789.84	788.59	787.52
720.41	728.20	727.37
630.72	632.84	636.38
602.75	596.97	600.17

[a] Scaling factor of 0.9614
[b] Scaling factor of 0.957

S52 IR theoretical calculations of 4

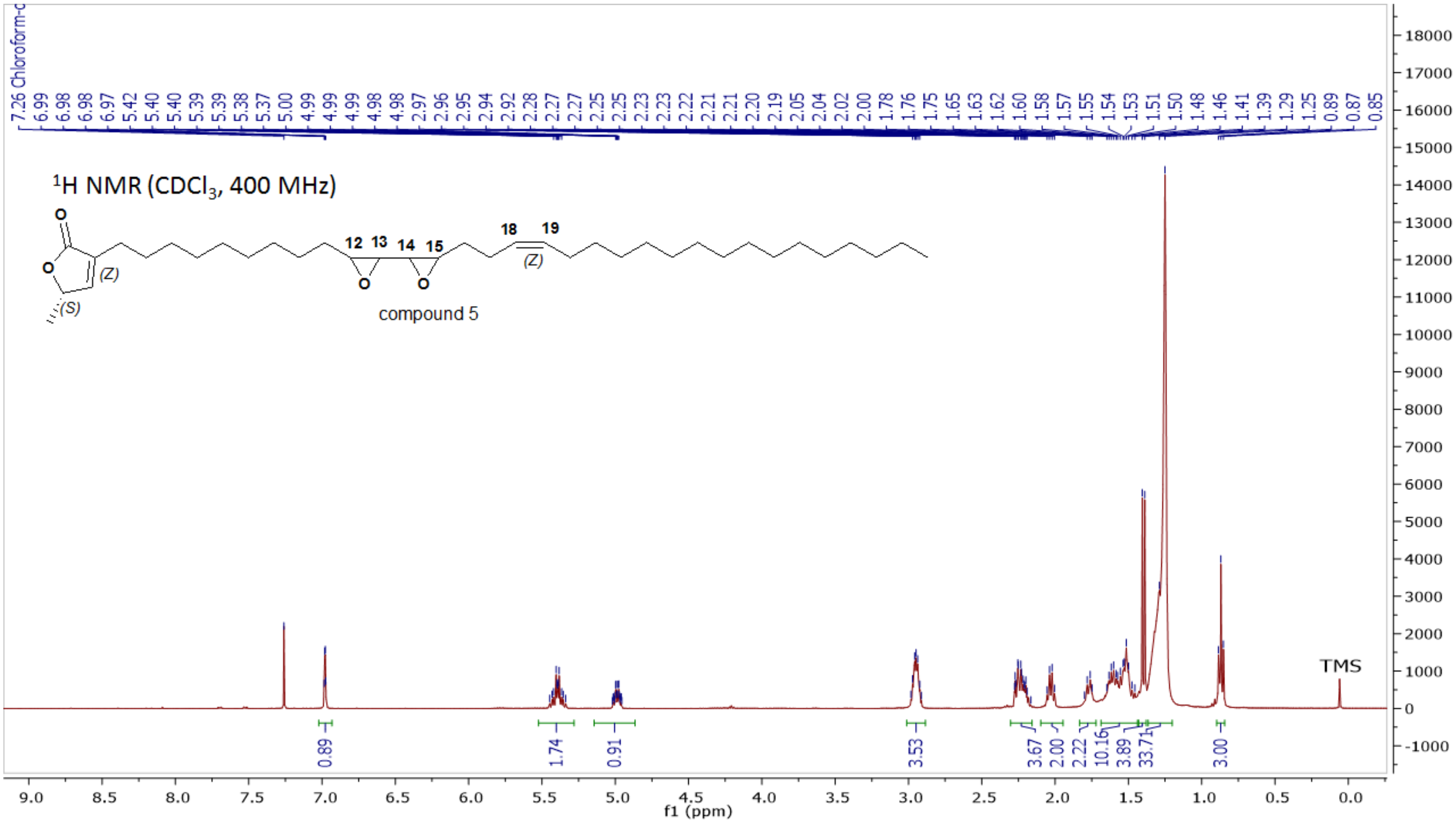

S53 ^{1}H NMR (400 MHz, $CDCl_3$) spectrum of compound 5

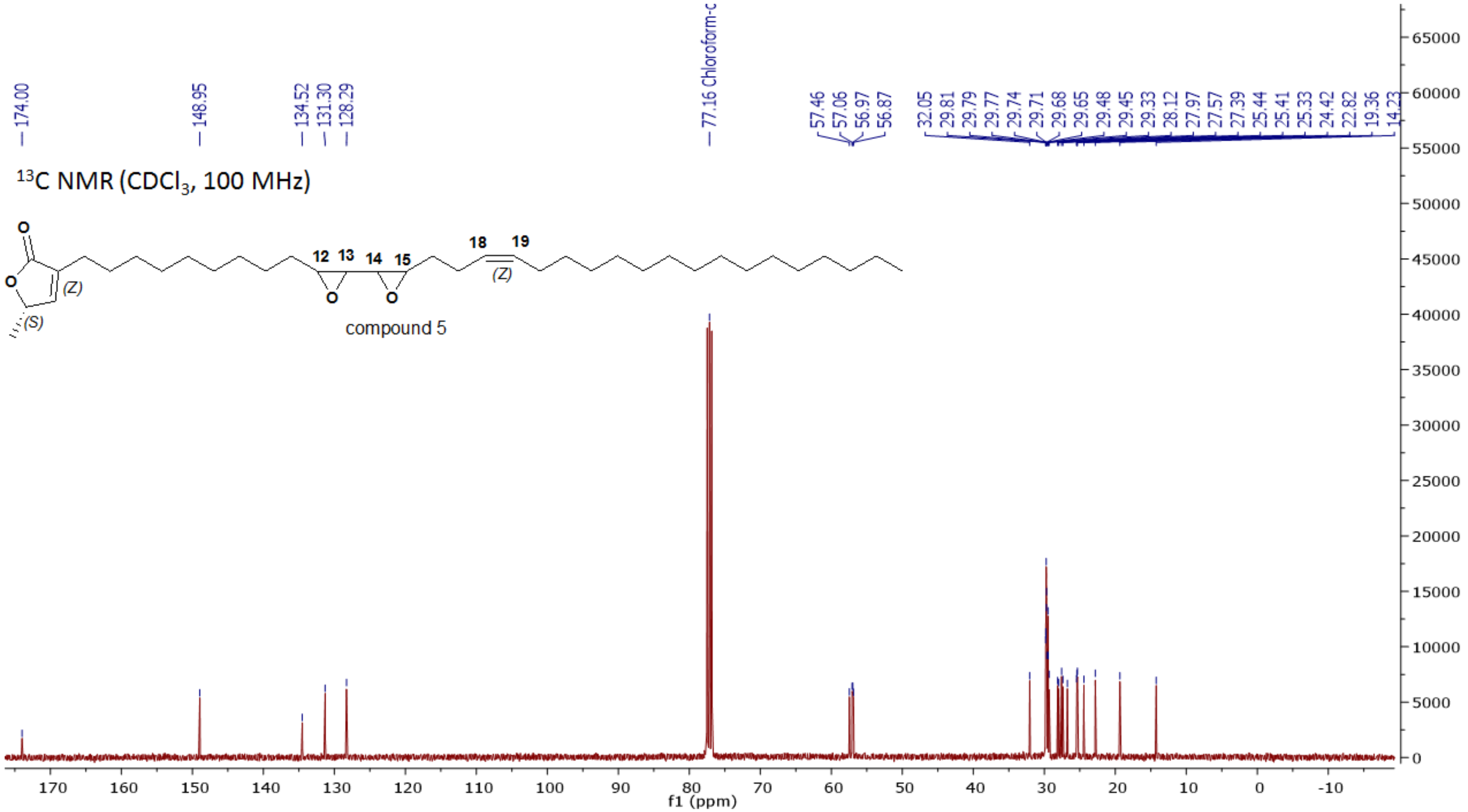

S54 ^{13}C NMR (100 MHz, $CDCl_3$) spectrum of compound 5

COSY experiment ($CDCl_3$)

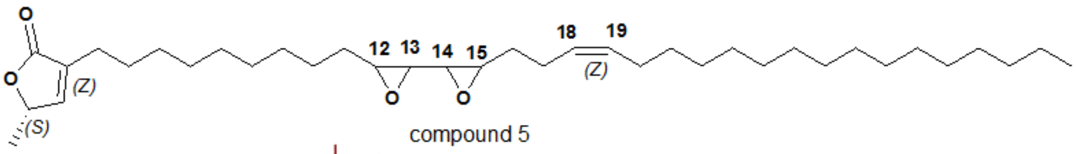

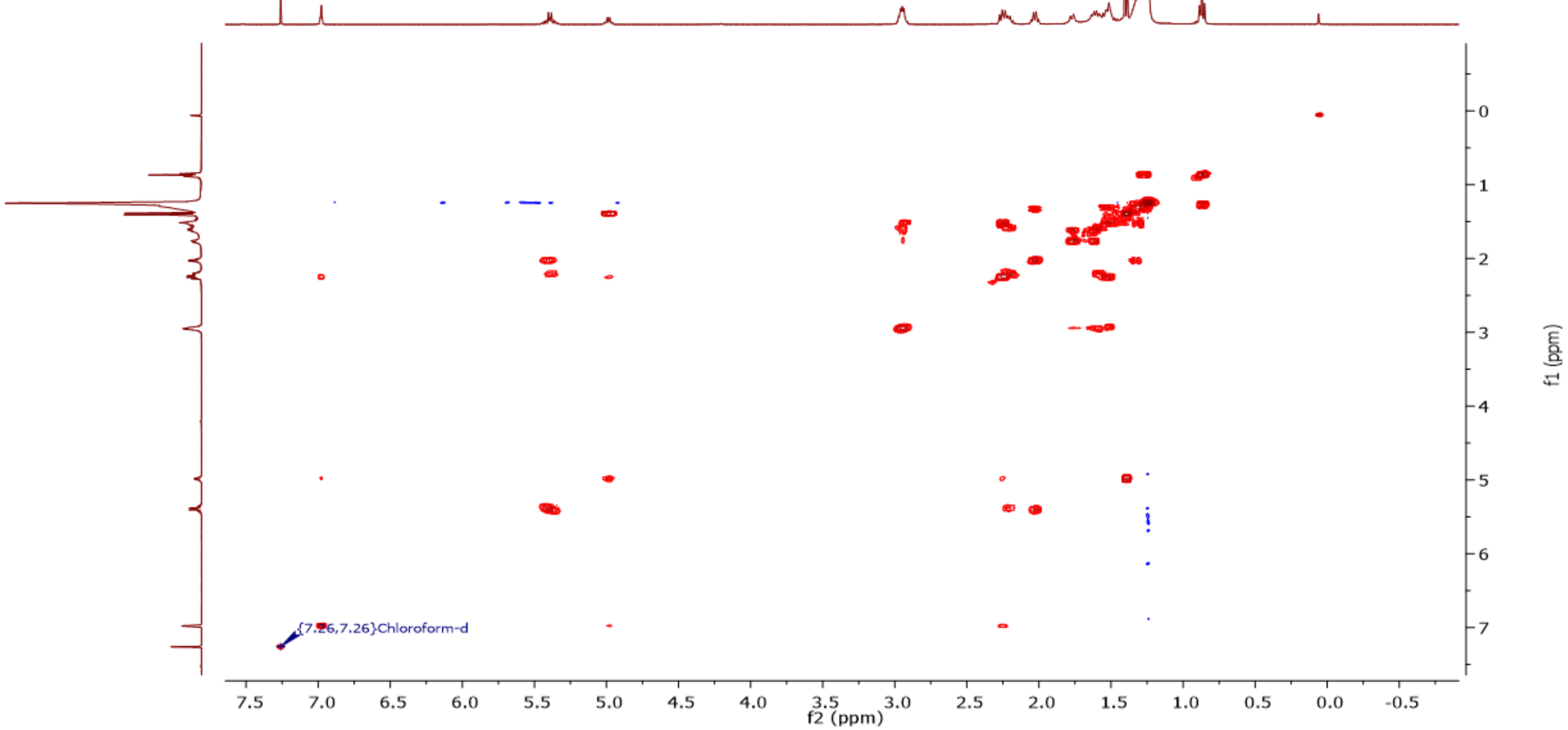

S55 COSY experiment ($CDCl_3$) of 5

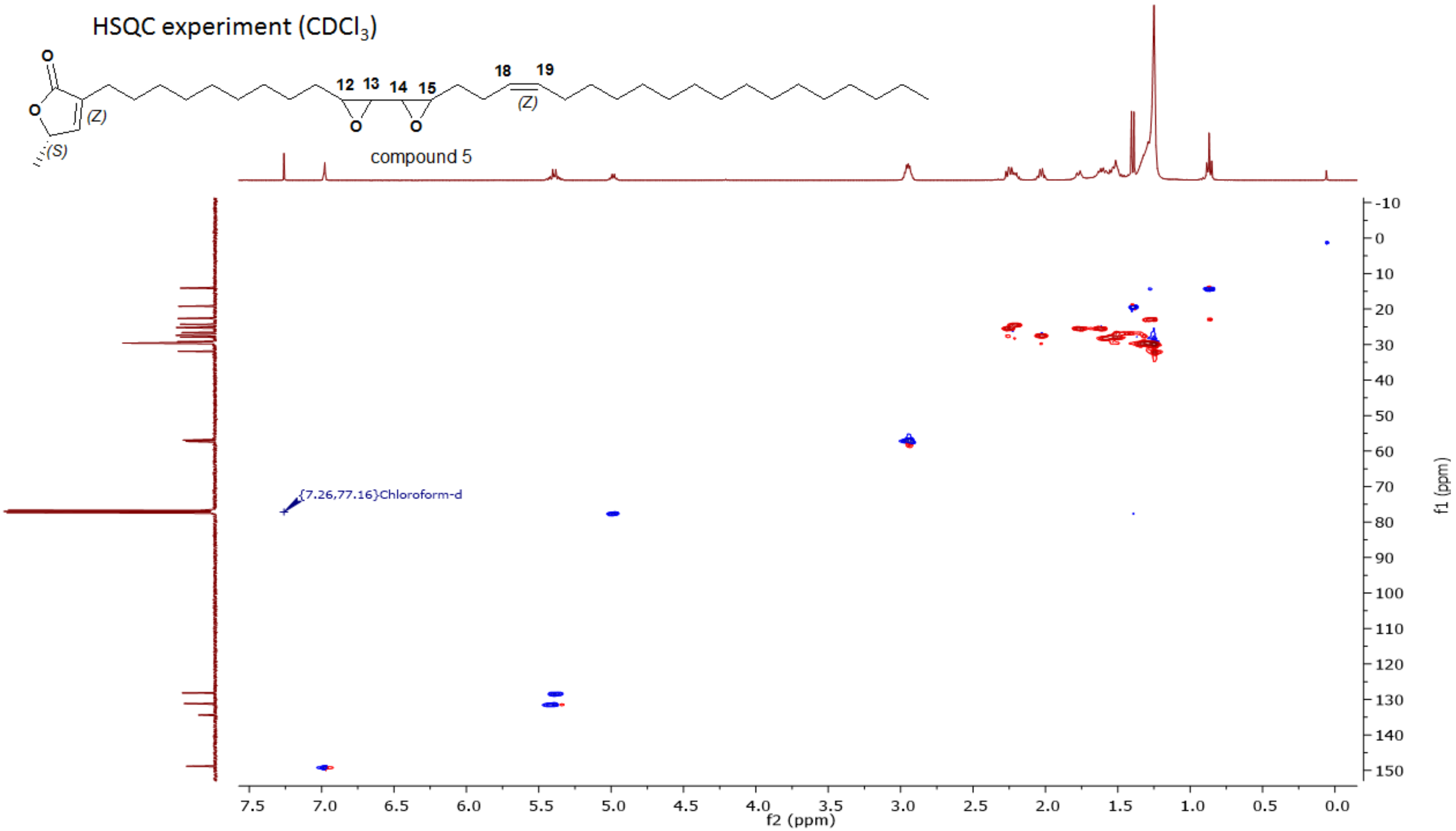

S56 HSQC experiment ($CDCl_3$) of 5

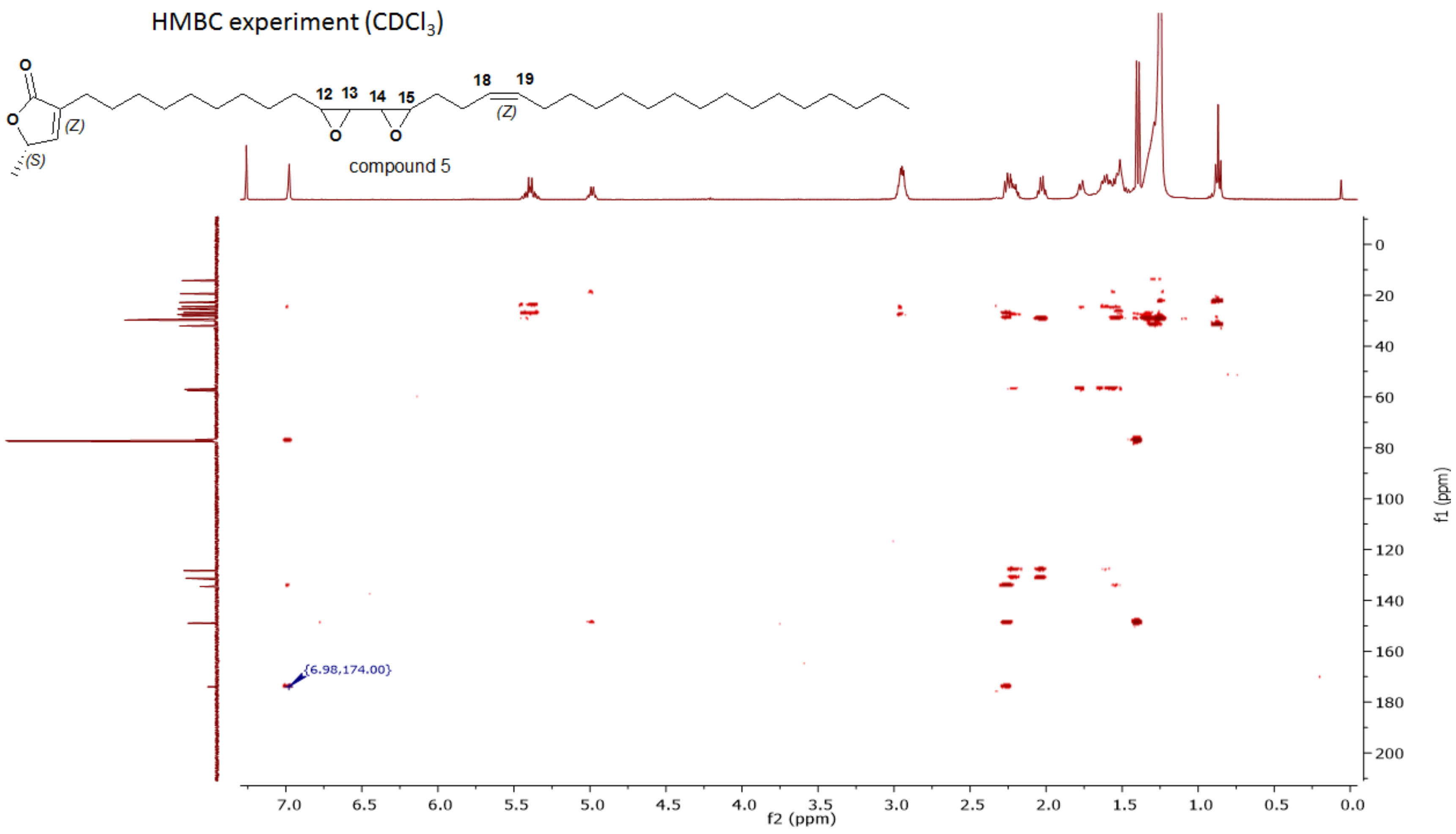

S57 HMBC experiment ($CDCl_3$) of 5

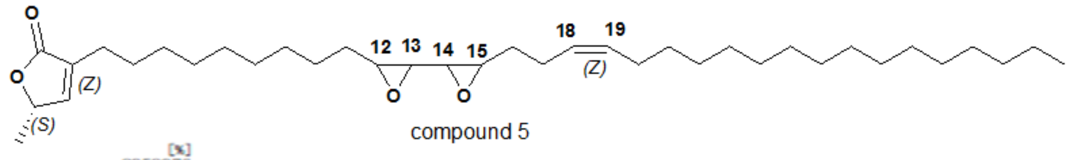

compound 5

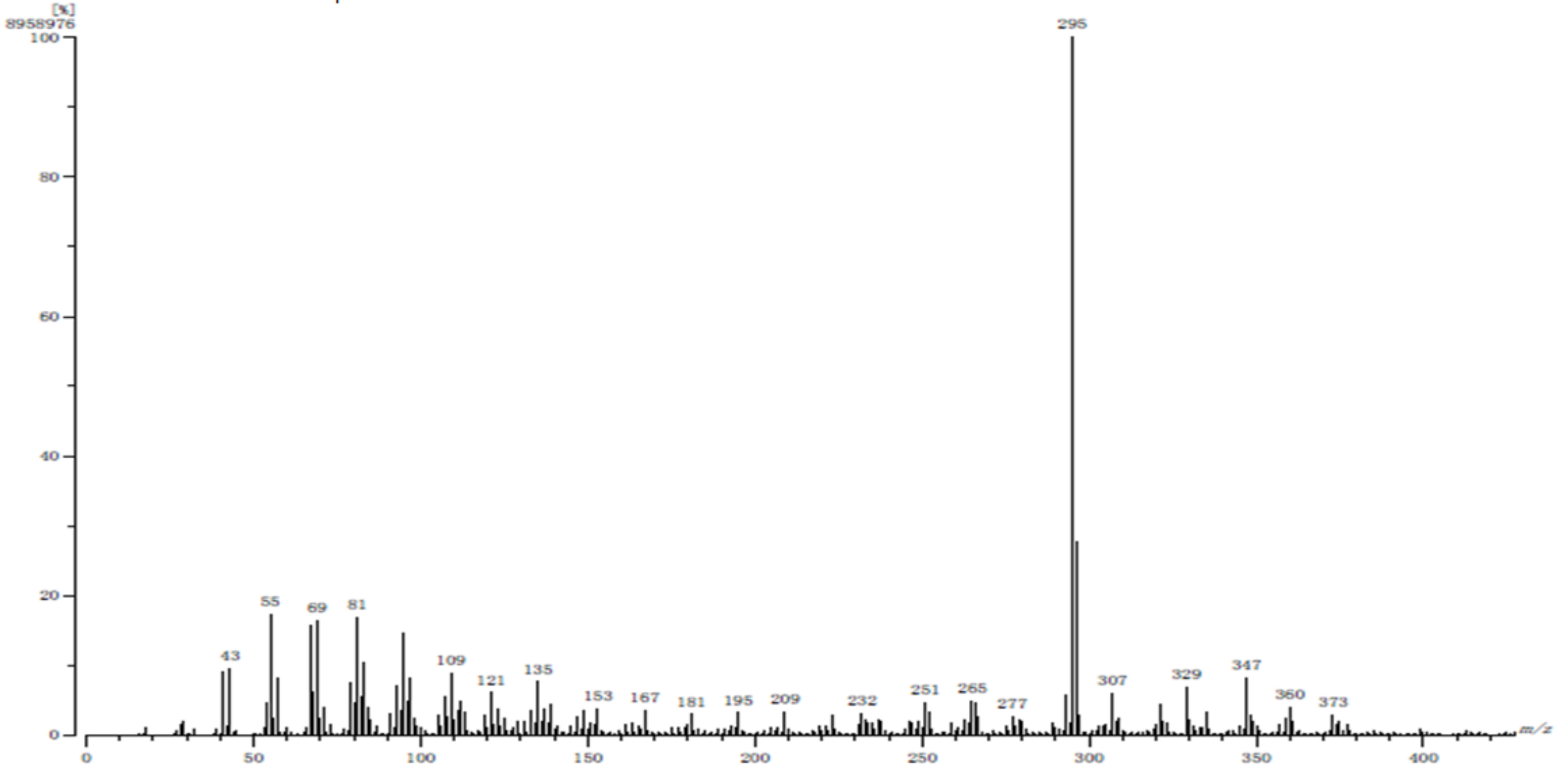

S58 Mass spectrum (IE) of 5

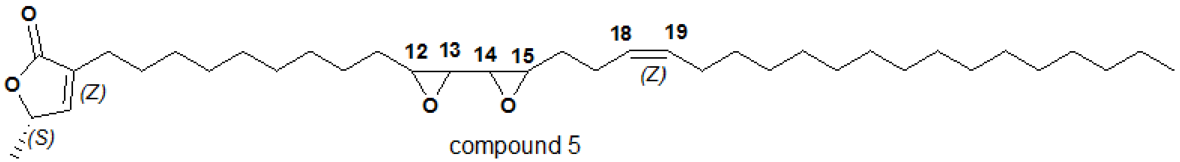

compound 5

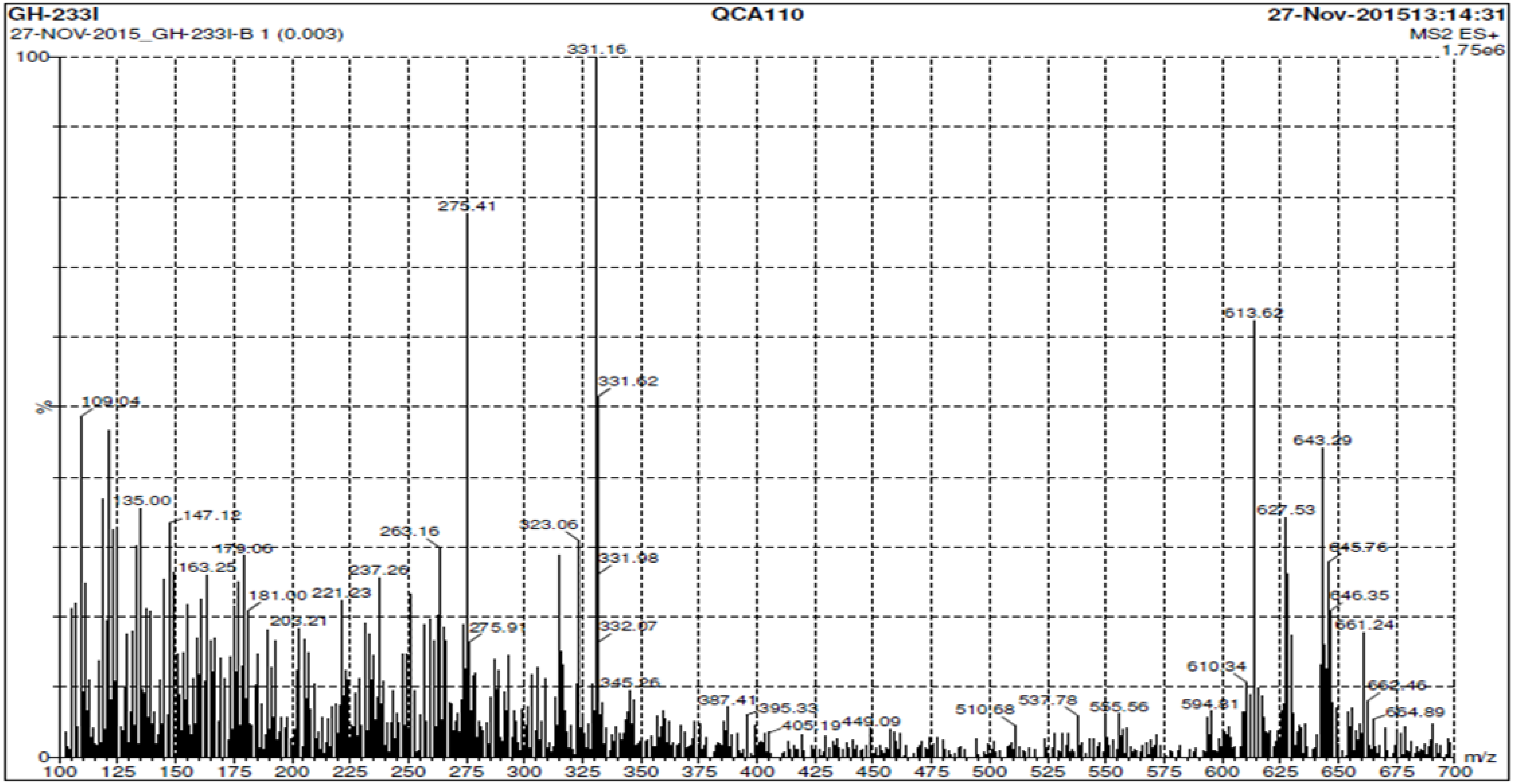

S59 Mass spectrum (ESI$^+$) of 5

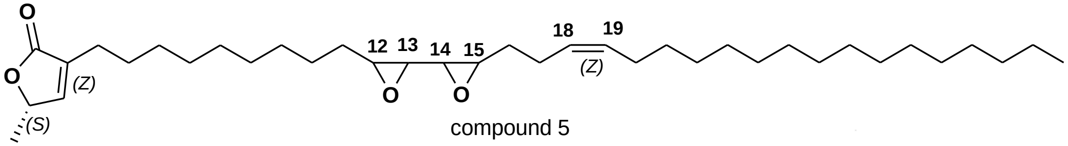

compound 5

Elemental composition calculator

```
Target m/z:        +595.4693      amu
Tolerance:           +3.0000      ppm
Result type:       Elemental
Max num of results:       1000
Min DBE:      -0.5000     Max DBE:        +100.0000
Electron state:   Even
Num of charges:   1
Add water:        N/A
Add proton:       N/A
File Name:        J-210618-Ar-1-13-01.wiff
```

	Elements	Min Number	Max Number
1	C	0	50
2	H	0	70
3	N	0	5
4	O	0	5
5	Na	0	2

	Formula	Calculated m/z (amu)	mDa Error	PPM Error	DBE
1	C37 H64 O4 Na	595.469682	-0.416240	-0.699011	5.5

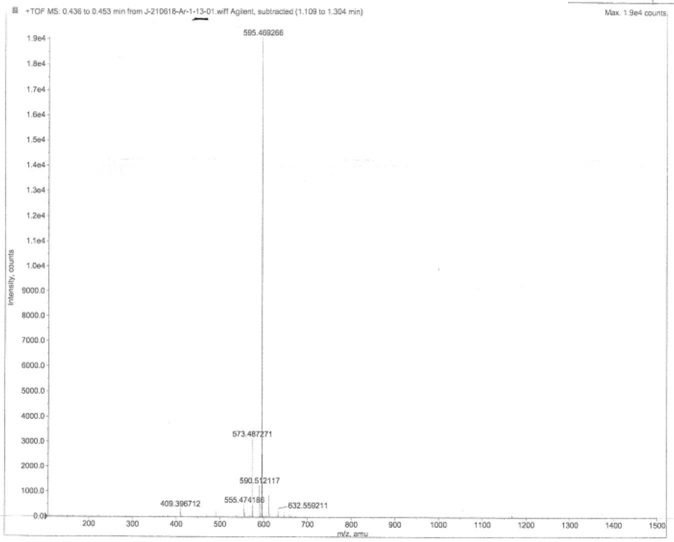

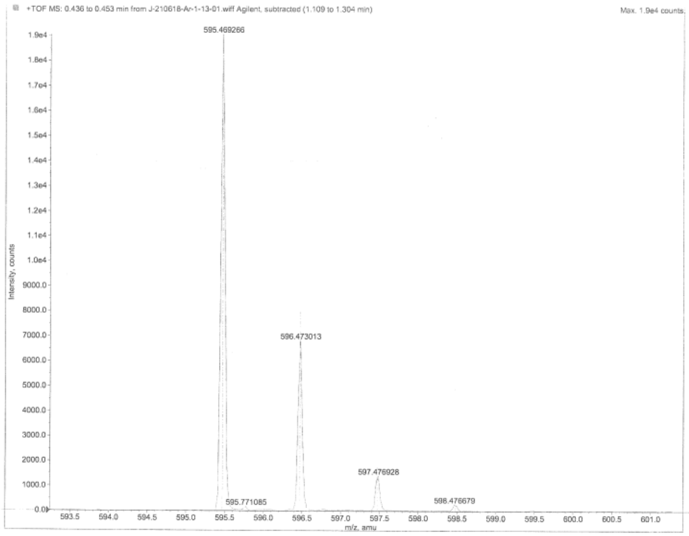

S60 HRMS (ESI-TOF) of compound 5

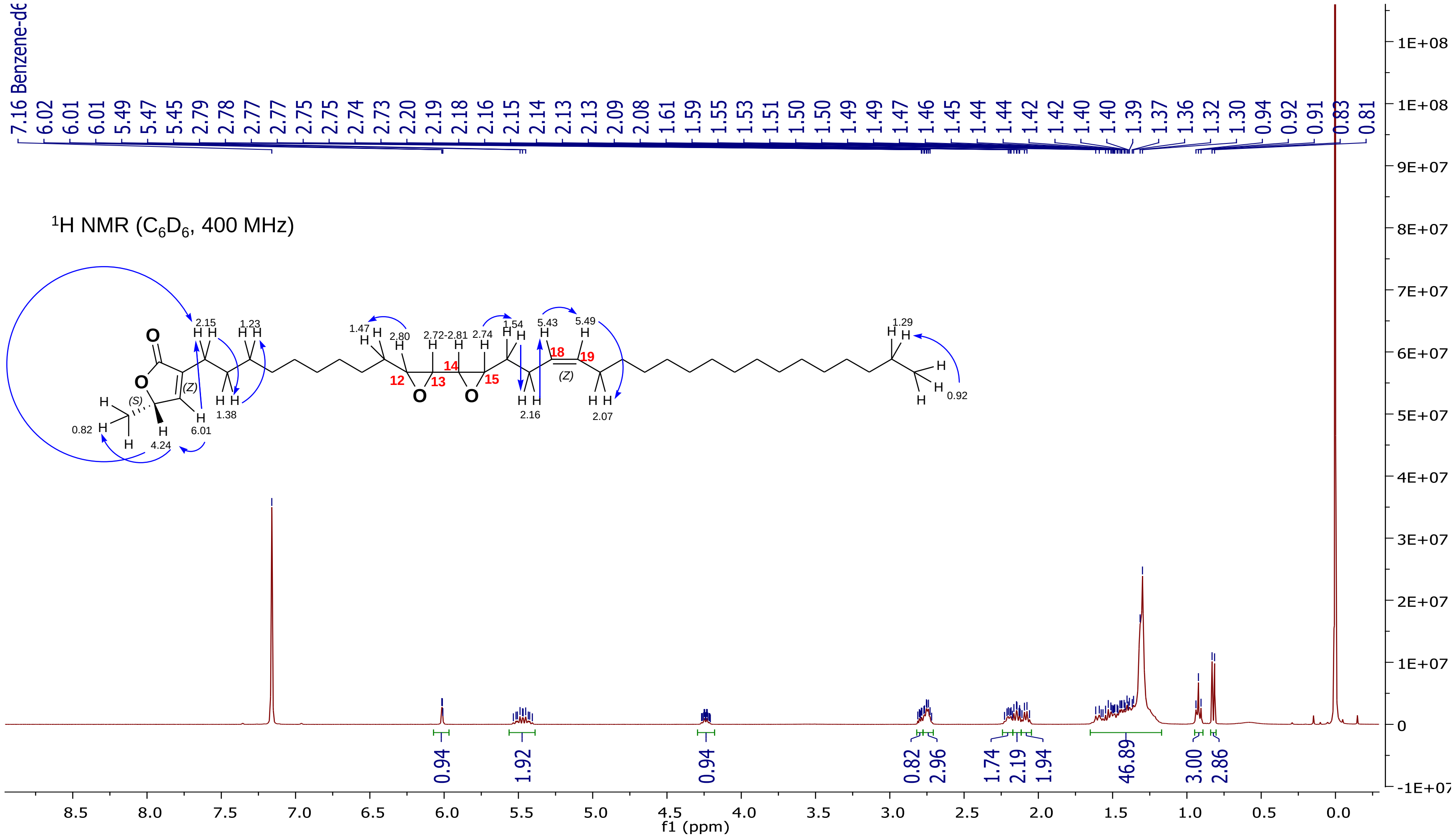

S61 Correlations in COSY and ^{1}H NMR spectrum (C_6D_6) of 5

Annopurpuricin E		
Experimental	6-311G(d,p)/B3LYP[a]	6-311G(d,p)/ωB97XD[b]
3013.76	3074.60	3019.94
2980.01	2981.73	2983.53
2951.08	2950.63	2952.11
2915.38	2919.36	2915.83
2848.85	2875.84	2887.62
1748.86	1767.87	1806.92
1655.92	1655.71	1676.93
1474.57	1460.26	1469.09
1462.03	1459.29	1460.94
1390.67	1383.38	1418.87
1310.07	1314.84	1312.40
1258.54	1260.01	1256.49
1207.43	1206.71	1214.16
1112.92	1126.46	1111.95
1069.52	1072.59	1072.78
1023.23	1024.15	1024.42
955.72	955.83	954.35
915.22	918.49	925.69
878.52	875.34	879.21
831.31	839.55	844.90
800.45	795.46	806.31
729.09	733.90	734.66
662.54	674.42	637.94

[a] Scaling factor of 0.9614
[b] Scaling factor of 0.957

S62 IR theoretical calculations of 5
